# Synthesis of Novel Chloro-Benzo [*d*]imidazole Regioisomers as Selective CB_2_ Receptor Agonists: Indirect Functional Evaluation and Molecular Insights

**DOI:** 10.3390/ph18111599

**Published:** 2025-10-22

**Authors:** Valeria Zuñiga Salazar, Renato Burgos Ravanal, Jonathan Soto-Flores, Gianfranco Sabadini, José Vicente González, Jaime Mella, Javier Romero-Parra

**Affiliations:** 1 Departamento de Química Orgánica y Fisicoquímicas, Facultad de Ciencias Químicas y Farmacéuticas, Universidad de Chile, Olivos 1007, Santiago 8380544, Chile; vale-paz45@hotmail.com (V.Z.S.); jonathan.soto@uchile.cl (J.S.-F.); 2 School of Pharmacy, Faculty of Chemistry and Pharmacy, Pontificia Universidad Católica de Chile, Vicuña Mackenna 4860, Santiago 7820436, Chile; renatoburgosravanal@gmail.com (R.B.R.); jvgonzal@uc.cl (J.V.G.); 3 Instituto de Química, Facultad de Ciencias, Universidad de Valparaíso, Av. Gran Bretaña 1111, Valparaíso 2360102, Chile; gianfranco.sabadini@postgrado.uv.cl; 4 Centro de Investigacion, Desarrollo e Innovacion de Productos Bioactivos (CInBIO), Universidad de Valparaiso, Av. Gran Bretaña 1111, Valparaíso 2360102, Chile

**Keywords:** cannabinoids, benzo [*d*]imidazole, cytotoxic studies, flow cytometry, indirect agonism, docking, molecular dynamics

## Abstract

**Background/Objectives:** The cannabinoid type 2 receptor (CB_2_ receptor) has been extensively studied in recent years due to the benefits associated with its modulation, including the regulation of the inflammatory response, neuroimmunomodulatory properties, and antitumor effects, all with the advantage of lacking significant psychoactive effects. Herein, we report the design, synthesis, characterization, biological assays, and molecular modelling analyses of novel (5/6-chloro-2-aryl-1*H*-benzo [*d*]imidazol-1-yl)(4-methoxyphenyl)methanone and 5/6-chloro-1-(4-methoxybenzyl)-2-aryl-1*H*-benzo [*d*]imidazole regioisomers as potential cannabinoid type 2 receptor ligands. **Methods:** The compounds were evaluated for their presumed CB_2_ agonist activity using an indirect receptor-dependent apoptotic cell death assay exerted by cannabinoids, using the cell lines HEK293 (low CB_1_/CB_2_ expression), U-87 MG (high CB_1_ expression), and HL-60 (exclusive CB_2_ expression), and including the known cannabinoid ligands WIN-55,212-2 and AM630 as reference ligands. Flow cytometry was performed to assess apoptosis. Molecular docking and molecular dynamics simulations were used to explore ligand-receptor interactions at the CB_2_ active site. **Results:** Compounds **3a**, **3b’**, **3c**, and **4b** selectively reduced HL-60 cell viability, similar to WIN-55,212-2, while showing no toxicity toward HEK293 or U-87 MG cells. Flow cytometry indicated that compounds **3a** and **3c** induced apoptosis in HL-60 cells comparable to WIN-55,212-2. Computational studies suggested that both compounds bind within the CB_2_ receptor active site predominantly through π–π and hydrophobic interactions involving their benzo [*d*]imidazole cores, 2-aryl moieties, and 4-methoxybenzoyl scaffolds, resembling the binding patterns of established CB_2_ ligands. **Conclusions:** Compounds **3a** and **3c** exert selective cytotoxicity against HL-60 cells, likely via a CB_2_ agonist-mediated apoptotic mechanism. The applied combined experimental and computational approach provides a rapid, informative strategy for preliminary evaluation of CB_2_ ligands and guides subsequent detailed pharmacological studies.

## 1. Introduction

The endocannabinoid system (ECS) is a neuromodulatory network widely distributed throughout the body, playing important roles in the central nervous system, synaptic plasticity, and the response to environmental stimuli. The ECS is primarily composed of endogenous biomolecules known as endocannabinoids, along with proteins and enzymes responsible for regulating their levels (i.e., NAPE-PLD, DAGL, FAAH, and MAGL) [1]. Two different G protein-coupled receptors (GPCRs) are the main proteins in this system: the cannabinoid receptor type 1 (CB_1_ receptor) [2], which is highly expressed in the brain and mediates the psychotropic effects of cannabinoids, and the cannabinoid receptor type 2 (CB_2_ receptor), which is primarily expressed in the immune system [3], although a low amount of this receptor has been recently reported in the brain [4]. Drug development targeting these receptors is of significant interest due to their involvement in many pathological conditions [5,6]. Modulating CB_1_ receptor provides several therapeutic and pharmacological options, including analgesia, tumor growth inhibition and treatment for epilepsy, and neuropathic pain, among others [7,8]. On the other hand, CB_2_ modulation has been studied for the treatment of autoimmune disorders, Huntington’s disease, and diverse cancer types [9,10]. It is also important to consider that CB_2_ receptor activation by agonists does not produce the psychoactive effects associated with CB_1_ receptor modulation [11,12]. Moreover, it is believed that CB_2_ receptors play a role in delaying the progression of neurodegenerative disorders [13]. Therefore, it is undeniable that CB_2_ receptor activation through agonists represents significant therapeutic potential for various pathologies and/or disorders, making it a highly attractive therapeutic target. In this sense, CB_2_ receptor management through synthetic compounds has garnered significant interest. Many authors have developed a wide range of synthetic compounds with high potency and selectivity for CB_2_ receptor [14,15,16]. Furthermore, these compounds have paved the way for enhancing the pharmacotherapeutic profile through studies that correlate the structure with the activity of the developed ligands, most of them being lipophilic molecules with aromatic heterocycles attached to bulky alkyl or aryl residues [17].

**Figure 1:**
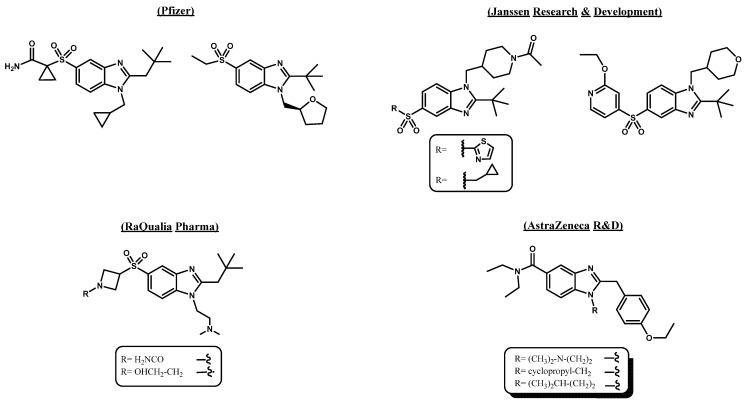
Cannabinoid ligands selective for the CB_2_ receptor containing the benzo [*d*]imidazole nucleus as the main core.

Within the synthetic ligands that exhibit selectivity for the CB_2_ receptor and act as agonists are the *classical cannabinoids* (CCs), JWH-051 [18] and L-759633 [19], as well as the *non-classical cannabinoids* (NCCs) HU-308 [20] and CP-55,940 [21], among others. Likewise, some *aminoalkylindoles* (AAIs), whose essential structural feature is to bear an indole ring, also exhibit selectivity for the CB_2_ receptor. Examples include JWH-015, which is nearly 28-fold more selective for CB_2_ over CB_1_ receptor [22], and AM-1241, which is 80-fold more selective for CB_2_ over CB_1_ [23,24,25]. In this regard, the benzo [*d*]imidazole, another important heterocycle, represents a crucial framework in drug discovery [26,27,28,29]. Therefore, benzo [*d*]imidazole-based compounds with affinity and pharmacological activity at both CB_1_ and CB_2_ receptors have been reported [30,31]. In particular, several selective CB_2_ benzo [*d*]imidazole agonists have been identified [32,33,34,35,36]. Figure 1 presents examples of selective CB_2_ benzo [*d*]imidazoles that have already been developed and reported.

Our research group has been dedicated to designing and synthesizing small molecules as chemical modulators of the endocannabinoid system (ECS). In particular, we have concentrated on the rational development of benzo [*d*]imidazole-based compounds that interact with the orthosteric active site of cannabinoid receptors. Additionally, we conducted molecular docking studies and quantitative structure–activity relationship (QSAR) analyses on the derivatives we obtained and reported [25,37,38,39,40]. Thus, this study presents the synthesis, biological assessment, docking studies and molecular dynamics (MD) of novel (5/6-chloro-2-aryl-1*H*-benzo [*d*]imidazol-1-yl)(4-methoxyphenyl)methanone and 5/6-chloro-1-(4-methoxybenzyl)-2-aryl-1*H*-benzo [*d*]imidazole regioisomers as CB_2_ ligands.

To evaluate the potential agonist activity of the synthesized compounds, cell viability assays were conducted using three distinct cell lines: human embryonic kidney cells (HEK293), characterized by low expression of both cannabinoid receptors [41]; glioblastoma cells (U-87 MG), which strongly express CB_1_ receptors [42]; and promyelocytic leukemia cells (HL-60), which exclusively express CB_2_ receptors [3]. This indirect approach was proposed based on several studies reporting that cannabinoid agonists induce apoptotic cell death [43,44,45,46,47,48,49]. Therefore, our synthesized compounds, along with the CB_1_/CB_2_ agonist WIN-55,212-2 [50], were tested on the three previously mentioned cell lines to evaluate their potential cytotoxicity. Consequently, the proposed indirect methodology aims to correlate the decrease in cell viability observed in cell lines expressing CB_1_ and CB_2_ receptors (U-87 MG and HL-60, respectively) upon treatment with WIN-55,212-2, as well as the decrease in viability in HL-60 cells treated with the synthesized regioisomers, given that these were developed as CB_2_-selective compounds. Likewise, the regioisomers should exhibit no toxicity in HEK293 cells. The latter observation suggests that selective cell death may be mediated by an agonist-induced cannabinoid receptor–dependent mechanism. Results showed that WIN-55,212-2 was toxic to U-87 MG and HL-60 cells, but not for HEK293; whereas AM630 [19] was non-toxic for HL-60 cells. Since WIN-55,212-2 induced toxicity in HL-60 cells, its effect was reversed when co-administered with increasing concentrations of the CB_2_ antagonist AM630, reinforcing the hypothesis that the cytotoxic effect is driven by cannabinoid receptor agonism. The synthesized regioisomers were mostly non-toxic to HEK293 and U-87 MG cells; nevertheless, compounds **3a**, **3b’**, **3c**, and **4b** exhibited selective toxicity toward the HL-60 cell line. Flow cytometry experiments (FC) exhibit that **3a** and **3c** were cytotoxic through an early apoptosis path, suggesting that these derivatives reduce HL-60 viability by an agonist-induced cannabinoid CB_2_ receptor.

## 2. Results and Discussion

### 2.1. Subsection Design Criteria to Develop CB_2_ Ligands

For the design of the sixteen proposed compounds as CB_2_ receptor ligands, the *aminoalkylindole* (AAI) cannabinoid ligands—such as WIN-55,212-2 and AM630—were used as reference. In this approach, the indole core was replaced with benzo [*d*]imidazole rings substituted with a chlorine atom, aiming to increase lipophilicity and mimic the iodine atom of AM630. Substitutions at the nitrogen atom 1 (-N1) and position 2 of the benzo [*d*]imidazole rings were selected based on the analysis of interactions between WIN-55,212-2 and the CB_2_ receptor active site, as reported by Xing et al. in their cryo-EM/WIN-55,212-2 complex study [51]. Therefore, the substitution at N1 in our design plan was consistently a 4-methoxyphenyl group attached to the benzo [*d*]imidazole ring linked either through a carbonyl group—introducing conformational restriction due to resonance and partial double bond character—or via a methylene linker, which permits greater conformational flexibility, aiming to evaluate the impact of these structural differences on biological activity. If interactions of our derivatives within the CB_2_ receptor active site are similar to those of WIN-55,212-2, the 4-methoxyphenyl moiety is expected to establish π–π interactions with Phe91 and Phe94, as well as hydrophobic contacts with Phe87, His95, Pro184, and Phe281.

Position 2 of benzo [*d*]imidazole scaffolds was substituted with the aromatic rings 3-methoxybenzene, 3-pyridine, furan, and isoxazole, which are bulky, planar, π-conjugated, and contain electronegative oxygen and/or nitrogen atoms. Based on the findings reported by Xing et al., these aromatic moieties may engage in hydrophobic interactions with residues Phe183, Ile186, and Trp194 of the CB_2_ receptor.

**Figure 2:**
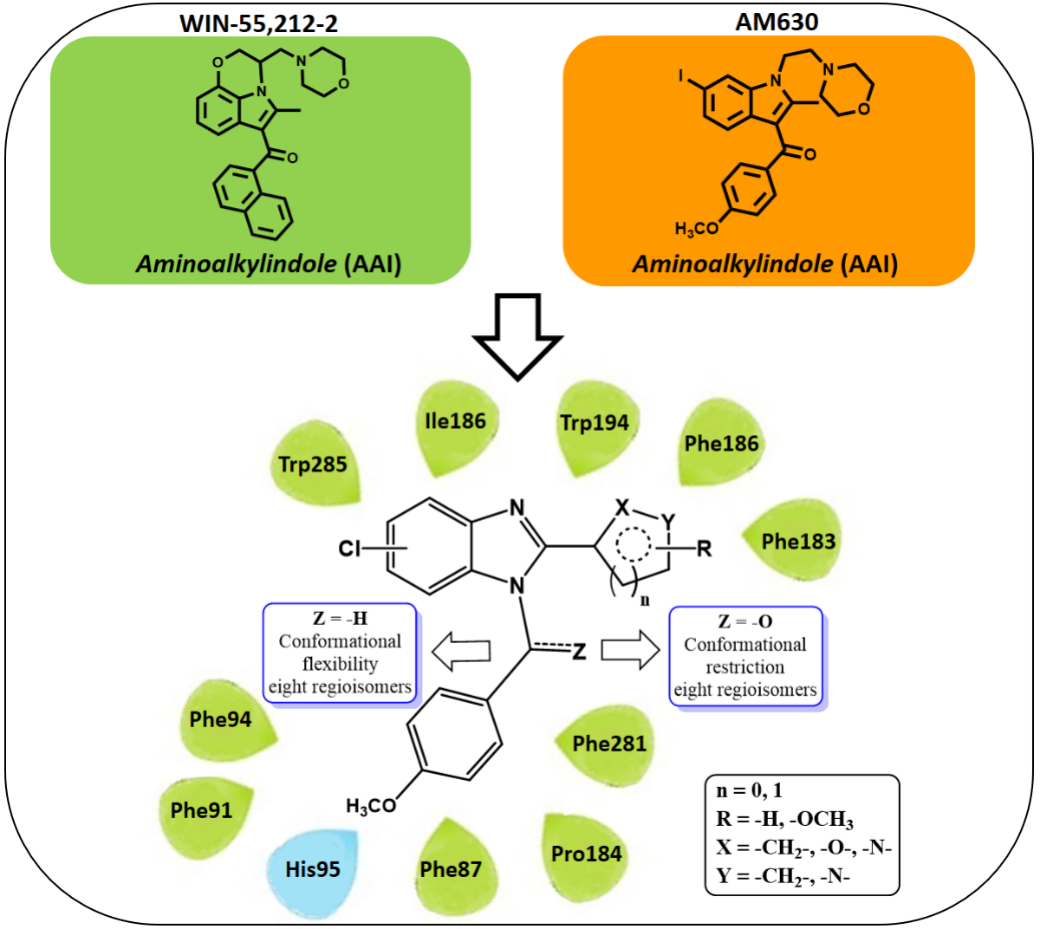
Design strategy for obtaining (5/6-chloro-2-aryl-1*H*-benzo [*d*]imidazol-1-yl)(4-methoxyphenyl)methanone and 5/6-chloro-1-(4-methoxybenzyl)-2-aryl-1*H*-benzo [*d*]imidazole regioisomers as CB_2_ ligands based on the structures of the *aminoalkylindoles* WIN-55,212-2, AM630 and the CB_2_ receptor active site reported by Xing et al.

Although WIN-55,212-2 and AM630 contain a morpholine moiety, our development plan did not include the incorporation of aliphatic or heterocyclic aliphatic groups. Instead, we focused on aromatic scaffolds as substituents within the benzo [*d*]imidazole core, particularly at the 2-position. The selected aromatic rings, as previously discussed, are also expected to interact with the same amino acid residues targeted by the morpholine nucleus of AAIs while providing the advantages of relative synthetic accessibility and ease of modification. Finally, the benzo [*d*]imidazole frameworks may also engage in π–π stacking interactions with Phe117, Trp194, and Trp258, as well as hydrophobic contacts with some residues such as Ile110, Val113, and Phe183, among others. Figure 2 shows a representation of the design strategy employed for the development of the proposed (5/6-chloro-2-aryl-1*H*-benzo [*d*]imidazol-1-yl)(4-methoxyphenyl)methanone and 5/6-chloro-1-(4-methoxybenzyl)-2-Aryl-1*H*-benzo [*d*]imidazole regioisomers as CB_2_ ligands.

### 2.2. Chemistry

**Scheme 1:**
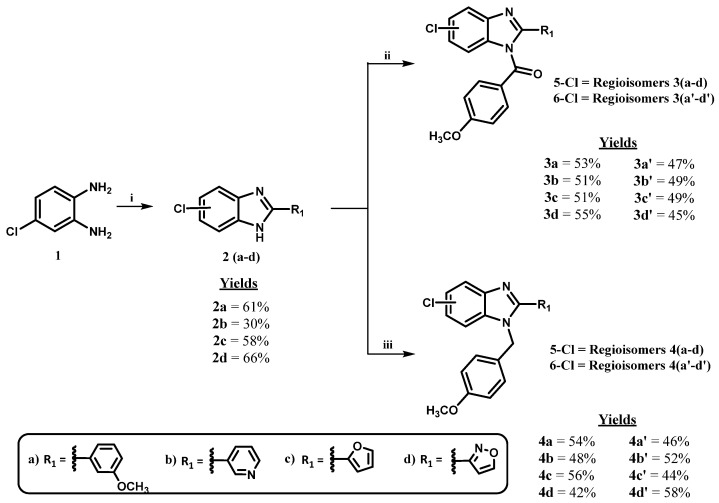
Synthesis of (5-chloro-2-(aryl)-1*H*-benzo [*d*]imidazol-1-yl)(4-methoxyphenyl)methanone **3(a-d)**, its regioisomers **3(a’-d’)** and 5-chloro-1-(4-methoxybenzyl)-2-aryl-1*H*-benzo [*d*]imidazole **4(a-d)** and its regioisomers 6-chloro-1-(4-methoxybenzyl)-2-aryl-1*H*-benzo [*d*]imidazole **4(a’-d’)**. Reagents and conditions: (i) for aldehydes: DMF, MgCl_2_·6H_2_O, rt, 24 h; for carboxylic acids: polyphosphoric acid (PPA), 180 °C, 3 h. (ii) 4-methoxybenzoyl chloride, NaH, N_2_, dry THF, rt, 30 min. (iii) 4-methoxybenzyl bromide, NaH, N_2_, dry THF, rt, 24 h.

To obtain (5-chloro-2-(aryl)-1*H*-benzo [*d*]imidazol-1-yl)(4-methoxyphenyl)methanone (**3a-d**) and its regioisomers (6-chloro-2-(aryl)-1*H*-benzo [*d*]imidazol-1-yl)(4-methoxyphenyl)methanone (**3a’-d’**), as well as the 5-chloro-1-(4-methoxybenzyl)-2-aryl-1*H*-benzo [*d*]imidazole **4(a-d)** and its regioisomers 6-chloro-1-(4-methoxybenzyl)-2-aryl-1*H*-benzo [*d*]imidazole **4(3a’-d’)** the following strategy was employed, as shown in Scheme 1. All regioisomers feature the aromatic rings 3-methoxybenzene, 3-pyridine, furan, and isoxazole at position 2 of the benzo [*d*]imidazole framework. Additionally, they bear a ketone or a methylene bridge attached to the nitrogen atom at position 1 of the main heterocycle, which connects to a 4-methoxyphenyl moiety.

The first synthetic step depicted in Scheme 1 illustrated the formation of 2-aryl-1*H*-benzo [*d*]imidazole rings, affording chlorinated intermediates **2**(**a-d**) via the condensation of 4-chloro-*o*-phenylenediamine (**1**) with various aldehyde derivatives in the presence of magnesium chloride hexahydrate (MgCl_2_·6H_2_O) [52]. MgCl_2_·6H_2_O neither promotes byproduct formation nor impedes the progression of intermediate species [53]. To synthesize the intermediate 3-((5 or 6)-chloro-1*H*-benzo [*d*]imidazol-2-yl)-4,5-dihydroisoxazole (**2d**), isoxazole-3-carboxylic acid was condensed with 4-chloro-o-phenylenediamine (**1**) in the presence of polyphosphoric acid (PPA), as described by Hein et al. [54].

**Figure 3:**
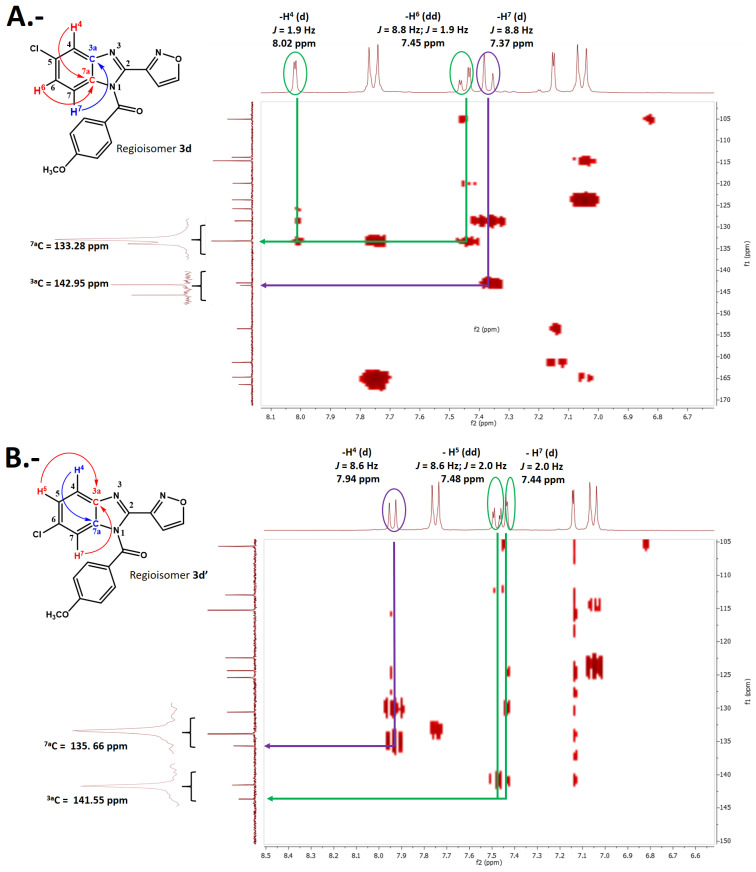
Heteronuclear Multiple Bond Correlation spectrum (HMBC) of regioisomers. (**A**) Compound 3d: Hydrogen atom multiplicity: –H^4^, doublet (d), *J*_1_ = 1.9 Hz at 8.02 ppm; –H^6^, doublet of doublets (dd), *J*_1_ = 8.8 Hz, *J*_2_ = 1.9 Hz at 7.45 ppm; –H^7^, doublet (d), *J*_1_ = 8.8 Hz at 7.37 ppm. (**B**) Compound 3d’: Hydrogen atom multiplicity: –H^4^, doublet (d), *J*_1_ = 8.6 Hz at 7.94 ppm; –H^5^, doublet of doublets (dd), *J*_1_ = 8.6 Hz, *J*_2_ = 2.0 Hz at 7.48 ppm; –H^7^, doublet (d), *J*_1_ = 2.0 Hz at 7.44 ppm. The main interactions among quaternary carbons and hydrogen atoms of benzo [*d*]imidazole rings in both regioisomers **3d** and **3d’** are highlighted, corroborating the position of the chlorine atom on both compounds.

In the second step of reactions depicted in Scheme 1, the final regioisomeric compounds, **3(a-d)**, **3(a’-d’)**, **4(a-d),** and **4(a’-d’)** were obtained using sodium hydride (NaH). This reagent generated the corresponding 2-aryl-1*H*-benzo [*d*]imidazole anions in situ. Each benzo [*d*]imidazole anion underwent nucleophilic attack on either 4-methoxybenzoyl chloride or 4-methoxybenzyl bromide, yielding the regioisomers mentioned above, in which the chlorine atom is located at the 5- or 6-position of the benzo [*d*]imidazole moieties. Once the regioisomers were separated by silica gel column chromatography, the exact structure of each regioisomer was determined through characterization and identification, based on their nuclear magnetic resonance (NMR) spectral data in one dimension and two dimensions (^1^H, ^13^C, DEPT-135, COSY, HSQC, and HMBC). As an example, spectral analyses of compound **3d** and its regioisomer **3d’** are shown in Figure 3 to clarify their precise structures and determine the correct position of the chlorine atom.

The precise position of the chlorine atom on the benzo [*d*]imidazole frameworks of regioisomers **3d** and **3d’** was established through Heteronuclear Multiple Bond Correlation (HMBC) spectroscopy by identifying the hydrogen atoms in both benzo [*d*]imidazole cores, their chemical shifts (ppm), and their multiplicities (hydrogens highlighted in Figure 3A,B). The benzo [*d*]imidazoles exhibit three distinct aromatic protons, with different multiplicities appearing as doublets (d) and doublets of doublets (dd), with different coupling constants (*J*) depending on their positions on the benzene ring. In this sense, only the hydrogen at position 7- (–H^7^) of regioisomer **3d** could exhibit a doublet (d) multiplicity with a high coupling constant (*J_1_* = 8.8 Hz, see Figure 3A), interacting with quaternary carbon atom 3a at three bonds of distance, as it would be adjacent to the hydrogen at position 6-(–H^6^). Carbon 3a appears more deshielded at 142.95 ppm within the benzo [*d*]imidazole heterocycle compared to the quaternary carbon 7a at 133.28 ppm (Figure 3A). On the other hand, the same hydrogen atom (–H^7^) in regioisomer **3d’**, which also interacts with carbon 3a, can only exhibit a doublet (d) with a small coupling constant (*J_1_* = 2.0 Hz; see Figure 3B) due to its adjacency to carbon 6-, which bears the chlorine atom, and consequently interacts with hydrogen –H^5^ via a *meta* coupling. Hydrogen atoms (–H^4^, –H^5^, and –H^6^, as appropriate) showed different multiplicity patterns, as shown in Figure 3A,B.

The same assessments for regioisomers **3d** and **3d’** were conducted to determine regioisomers **3(a-c)** and **3(a’-c’)**, corresponding to derivatives substituted with 3-methoxybenzene, 3-pyridine, and 2-furan at position 2 of the benzo [*d*]imidazole scaffolds. Regioisomers **3(a-d)** bear chlorine atoms at position 5 of the main heterocycle, whereas regioisomers **3(a’-d’)** possess this atom at position 6 of the central heterocyclic core.

**Scheme 2:**
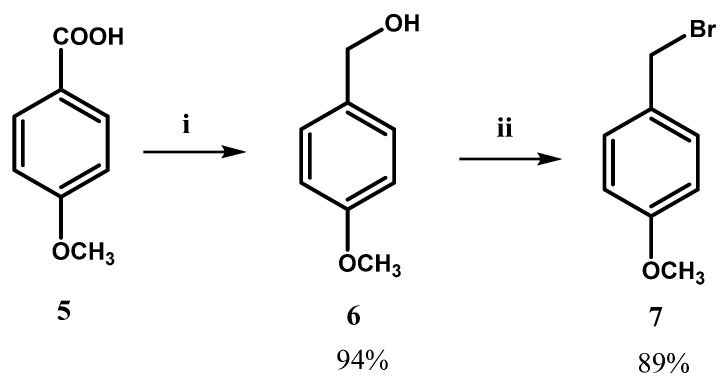
Synthesis of 4-methoxybenzyl bromide (**7**), which was used as reagent to obtain regioisomers **4(a-d)** and **4(a’-d’)**. Reagents and conditions: (i) LiAlH_4_, dry THF, 1 h. Then, HCl, 1N. (ii) PBr_3_, diethyl ether, 24 h.

On the other hand, regioisomers 5-chloro-1-(4-methoxybenzyl)-2-aryl-1*H*-benzo [*d*]imidazole **4(a-d)** and 6-chloro-1-(4-methoxybenzyl)-2-aryl-1*H*-benzo [*d*]imidazole **4(a’-d’)** were also synthesized under reaction conditions described in step **iii** of Scheme 1. Analogous to the synthesis of compounds **3(a-c)** and **3(a’-c’)**, the anions of benzo [*d*]imidazoles **2(a-d)** were generated in situ using NaH. Subsequently, each anion was reacted with the previously synthesized 1-(bromomethyl)-4-methoxybenzene (**7**). Synthesis of compound **7** from 4-methoxybenzoic acid (**5**) is shown in Scheme 2.

Analogous to isomers **3d** and **3d’**, regioisomers **4(a-d)** and **4(a’-d’)** were separated and subjected to one- and two-dimensional spectral analyses (2D-NMR analysis) to determine the position of the chlorine atom and elucidate the correct structure of each derivative. Once again, regioisomers **4(a-d)** exhibit the chlorine atoms at position 5 of the main benzo [*d*]imidazole heterocycle, whereas regioisomers **4(a’-d’)** bear them at position 6.

Finally, the strategy led to the successful synthesis of sixteen distinct benzo [*d*]imidazole-based regioisomers. Eight of them contain a carbonyl linker between the 4-methoxyphenyl and 2-arylbenzo [*d*]imidazole rings, while the other eight derivatives feature a methylene linker separating the aforementioned aromatic frameworks.

Once the structure of each compound was reliably determined, they were subjected to biological assays, including cell viability assays (MTT, (3-(4,5-dimethylthiazol-2-yl)-2,5-diphenyltetrazolium bromide) over three distinct and representative cell lines as models of the cannabinoid system. Therefore, human embryonic kidney cells (HEK293), representing a healthy cell line with low expression of both cannabinoid receptors [41]; glioblastoma cells (U-87 MG), characterized by high CB_1_ receptor expression [42]; and acute promyelocytic leukemia cells (HL-60), which selectively express CB_2_ receptors [3], were chosen. Subsequently, flow cytometry (FC) experiments were conducted to determine how compounds exert cytotoxicity—whether by promoting necrosis or apoptosis—in those compounds found to be cytotoxic toward the previously mentioned cell lines. This approach is based on the established role of CB_1_ and CB_2_ agonism in promoting apoptosis and suppressing tumor cell growth [43,44,45,46,47,48]. Regioisomers **4a** and **4a’** were excluded from biological assays due to their poor solubility in the media required for these experiments.

### 2.3. Biological Evaluations: Cell Viability (MTT) and Flow Cytometry (FC) Experiments

Various studies have demonstrated that the activation of CB_1_ and CB_2_ receptors by agonists leads to cell death and suppresses the proliferation of tumor cells. Namely, activation of CB_1_ and CB_2_ receptors (cannabinoid agonism) induces apoptosis and inhibits tumor cell growth [43,44,45,46,47,48]. Against the aforementioned background, cell viability experiments were conducted on different cell lines, some expressing cannabinoid receptors and others not. If an agonist selectively induces cytotoxicity in a cell line that expresses high levels of CB_1_ or CB_2_ receptors but not in a cell line lacking these receptors, the effect could be attributed to its activity as a cannabinoid agonist. As was mentioned, the cell lines used were HEK293 (human embryonic kidney cells), known for their low expression of both CB_1_ and CB_2_ receptors [41]; U-87 MG (glioblastoma cells), which exhibit high levels of CB_1_ receptor expression [42]; and HL-60 (promyelocytic leukemia cells), which exclusively express CB_2_ receptors [3].

**Figure 4:**
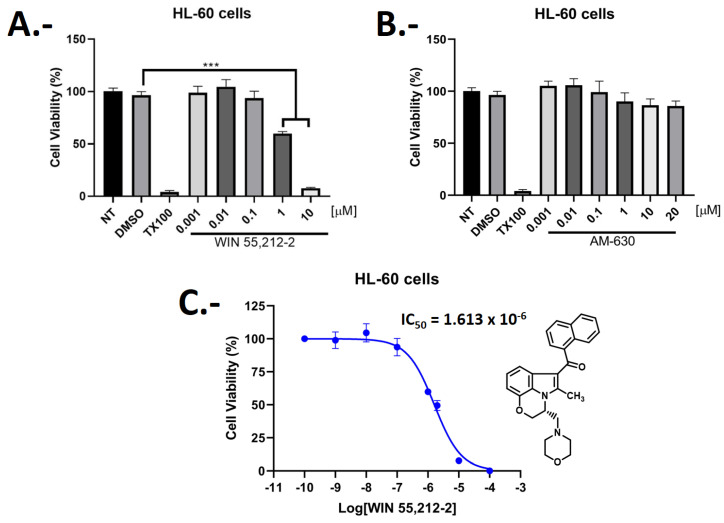
Cell viability [MTT-(3-(4,5-dimethylthiazol-2-yl)-2,5-diphenyltetrazolium bromide)] assays of synthetic cannabinoids. (**A**) Effect of WIN-55,212-2 (CB_1_/CB_2_ agonist) at increasing concentrations on HL-60 cells. (**B**) Effect of AM630 (CB_2_ inverse agonist/antagonist) at increasing concentrations on HL-60 cells. NT = untreated cell control; DMSO = solvent vehicle control; TX100 = known cell death control agent—Triton X-100. (**C**) Dose-response curve of WIN-55,212-2 showing the half-maximal inhibitory concentration (IC_50_) in HL-60 cell line, indicative of its cytotoxic effect. Data were normalized relative to the untreated cells and analyzed using One-Way ANOVA parametric test. The graphs show the averages of results from three independent experiments (mean ± standard error of the mean). Significant differences are indicated by *** *p* < 0.001.

Given the aforementioned, we conducted cytotoxic experiments over HL-60 cells of the CB_1_/CB_2_ agonist WIN-55,212-2 and the CB_2_ inverse agonist/antagonist AM630. Figure 4 shows that the CB_2_ inverse agonist/antagonist AM630 is non-toxic to HL-60 cells. However, WIN-55,212-2 exhibits cytotoxicity in these cell cultures. Our obtained cytotoxic half-maximal inhibitory concentration (IC_50_) of WIN-55,212-2 over HL-60 cells showed a value of 1.61 μM. Therefore, it is suggested that the observed cytotoxicity may result from a mechanism involving cannabinoid receptor activation, given that WIN-55,212-2 is a well-characterized agonist previously linked to the induction of cell death.

To further support our CB_2_-mediated agonist-induced cell death mechanism proposal, WIN-55,212-2 was tested in the presence of increasing concentrations of the CB_2_ inverse agonist/antagonist AM630, using a fixed concentration of 2.0 μM, corresponding to a near measured value of the IC_50_ previously determined for WIN-55,212-2 in our HL-60 cell cultures. The selected increasing concentrations (2 μM, 10 μM, and 20 μM) were intended to potentially enable the displacement of WIN-55,212-2, considering that this compound has been reported to exhibit K_i_ values ranging from 0.280 to 16.2 nM for the CB_2_ receptor [55,56], while AM630 has a K_i_ of 32.1 nM for this receptor [19]. Thus, using concentrations approximately 60-, 310-, and 620-fold higher than the K_i_ value of AM630 was expected to favor competitive displacement under the experimental conditions.

**Figure 5:**
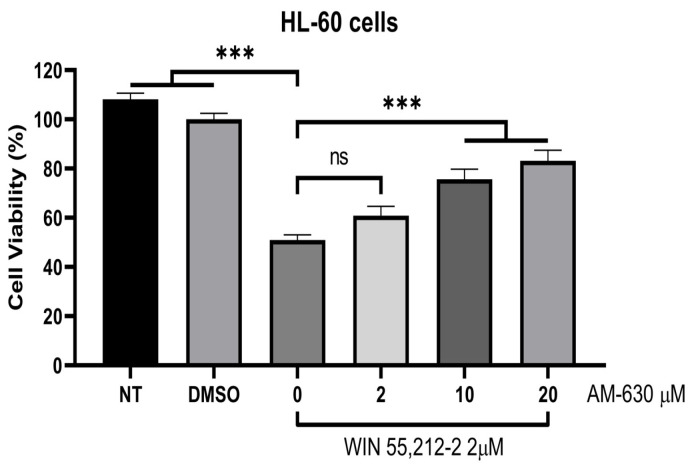
Cell viability experiments over human acute promyelocytic leukemia cells (HL-60) between WIN-55,212-2, at a predetermined concentration of 2.0 μM, and increasing concentrations of the CB_2_ inverse agonist/antagonist AM630. WIN-55,212-2-induced toxicity decreases in a concentration-dependent manner with increasing concentrations of the inverse agonist/antagonist AM630. NT = untreated cell control; DMSO = solvent vehicle control. Data were normalized relative to the vehicle used (DMSO) and analyzed using the one-way ANOVA parametric test. The graphs show the averages of results from three independent experiments (mean ± standard error of the mean). Significant differences are indicated by *** *p* < 0.001. ns = not significant.

Figure 5 presents the results of the assay involving WIN-55,212-2 and AM630, showing that as the concentration of AM630 increased, HL-60 cell viability also increased and WIN-55,212-2 became less toxic to these cells. These findings support the possibility that the agonist and antagonist may be competing for the same binding site and that the cytotoxic effect observed for the *aminoalkylindole* WIN-55,212-2 could be related to its agonistic activity at the CB_2_ receptor.

**Figure 6:**
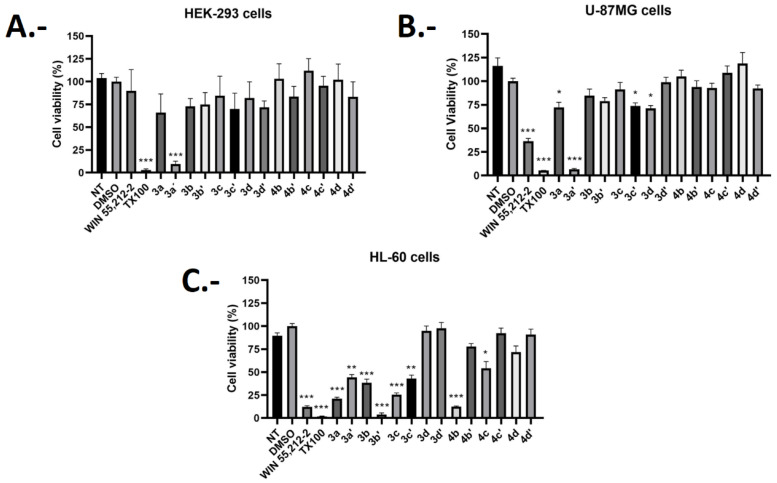
Cell viability [MTT, (3-(4,5-dimethylthiazol-2-yl)-2,5-diphenyltetrazolium bromide)] assays of fourteen obtained regioisomers at a concentration of 10 µM, as well as the known synthetic cannabinoid agonist WIN-55,212-2. (**A**) Cell viability of regioisomers in HEK293 cells. (**B**) Cell viability of regioisomers in U-87 MG cells. (**C**) Cell viability of regioisomers in HL-60 cells. NT = untreated cell control; DMSO = vehicle control; TX100 = known cell death control agent—Triton X-100 (0.2%). The graphs show the averages results from three independent experiments (mean ± SEM). Data were normalized relative to the vehicle used (DMSO) and analyzed using one-way ANOVA parametric test. Statistically significant differences are indicated * *p* < 0.05, ** *p* < 0.01 and *** *p* < 0.001.

Given the above, to assess the potential agonist behavior of our developed synthesized compounds, fourteen derivatives were subjected to cell viability experiments (as **4a** and **4a’** were excluded due to poor solubility) over HEK293, U-87 MG, and the HL-60 cells. Figure 6 shows the cell viability percentage values of the obtained fourteen regioisomers at a high concentration of 10 µM over the cell lines mentioned above. Assays were performed after 72 h of incubation, including an untreated cell control (NT), a solvent vehicle control (DMSO), a known cell death control, Triton X-100 (TX100), and the CB_1_/CB_2_ synthetic cannabinoid agonist WIN-55,212-2 at a concentration of 5 µM.

Figure 6 shows that, in HEK293 cells, most compounds do not significantly reduce cell viability at a concentration of 10 µM. In particular, compounds **3c**, **4b**, **4d**, **4c,** and **4c’** exhibit no cytotoxicity and would therefore be harmless to this healthy kidney cell line. Moreover, compounds **3b**, **3b’**, **3d**, **3d’**, **4b’,** and **4d’** reduce cell viability by approximately 30%, while compounds **3a** and **3c’** decrease the viability by 40%. However, given the high concentration used (10 µM), it is possible to affirm, as previously mentioned, that these compounds exhibit low toxicity in the HEK293 cell line. Similarly, WIN-55,212-2 reduces cell viability by approximately 10%, also demonstrating very low toxicity toward these cells. The only compound that shows a significant decrease in cell viability compared to the others is derivative **3a’**, with only 5.38% of the cells remaining viable after treatment with 10 µM of this compound, thus being considered a toxic agent for this cell type.

On the other hand, Figure 6 also shows the viability of each derivative in the U-87 MG cell line. Compounds **3c**, **3d’**, **4b**, **4b’**, **4c**, **4c’**, **4d,** and **4d’** exhibit minimal to no reduction in viability in U-87 MG cells. Additionally, compound **3b’** decreases cell viability by approximately 20%, while derivatives **3a**, **3c’,** and **3d** cause a nearly 30% reduction. Overall the regioisomers, similar to their effects on the HEK293 cell line, do not significantly reduce cell viability at a concentration of 10 µM. Regarding WIN-55,212-2, this agonist exhibits an average viability of 36.21%. When compared to results obtained for the HEK293 cell line, WIN-55,212-2 significantly reduces the number of viable U-87 MG cells. This outcome could be attributed to the agonist effects of WIN-55,212-2, considering the previously discussed characteristics of the U-87 MG cell line, which exhibits substantial CB_1_ receptor expression and, consequently, has the potential to promote a cannabinoid-mediated agonist-induced cell death mechanism [42].

Finally, significant changes in cell viability induced by synthesized derivatives in HL-60 cells can be observed. Derivatives **3d**, **3d’**, **4c’,** and **4d’** induce a decrease in viability of approximately 5% to 12%. Additionally, ligands **4b’** and **4d** cause a reduction in cell viability of 22.1% and 31.2%, respectively. In contrast, compounds **3a** and **3c** exhibit an average cell viability of 21.14% and 25.42%, respectively. Likewise, compounds **3a’**, **3b**, **3c’**, and **4c** also show a significant decrease in HL-60 cell viability compared to the other cell lines, with an average survival rate ranging from 54% to 40% approximately. Similarly, the synthetic isomer **4b** and the cannabinoid agonist WIN-55,212-2 display an average viability of approximately 12%, which is significantly lower than the previously mentioned values. In contrast, the regioisomer **3b’** shows a cell viability value of 3.8%, making it considerably more toxic. Again, it is plausible to suggest that the most cytotoxic compounds for HL-60 cells (derivatives **3a**, **3b’**, **3c**, and **4b**) may exert their effects through cannabinoid receptor agonism, specifically via CB_2_ activation, given the significant expression of this receptor in the cell line [3].

Considering the cell viability assays results, a cytotoxic selectivity index (% viability HL-60/% viability U-87 MG) was determined. This index ratio between the viability percentage parameters would provide some insight into the selectivity of the synthesized compounds for CB_2_ receptor over CB_1_ receptor, thereby allowing the selection of suitable compounds for further studies. Therefore, lower selectivity index values indicate a greater cytotoxic effect on HL-60 cells and, consequently, may suggest selectivity for CB_2_ receptor, while higher values indicate a lower cytotoxic effect on HL-60 cells (and/or higher cytotoxicity on U-87 MG cells), suggesting low selectivity for CB_2_ receptor. Table 1 summarizes the cell viability percentage across the three tested cell lines for each synthesized regioisomer, as well as the cytotoxic selectivity index (% viability HL-60/% viability U-87 MG) previously discussed.

Compounds **3a**, **3b’**, **3c,** and **4b** exhibited the lowest cytotoxic selectivity index values. Moreover, low cytotoxicity percentages in human embryonic kidney cells (HEK293) were observed for these derivatives. The latter corresponds to a favorable result, as the aim was to achieve selective cytotoxicity only for the HL-60 cell line. Although compound **3a** showed a relatively high reduction in HEK293 cell viability, reaching 39.16%, nevertheless, this toxicity level remains within a reasonable range considering the high concentration of 10 μM used.

**Figure 7:**
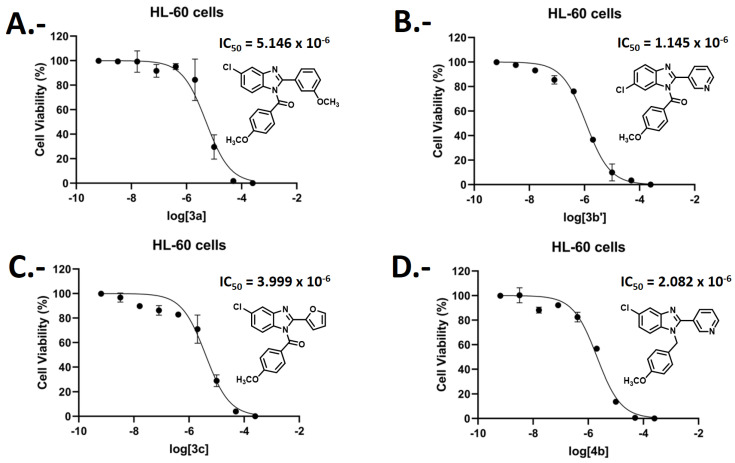
Dose–response curves and pharmacological toxicity half-maximal inhibitory concentration (IC_50_) of (**A**) regioisomer **3a**. (**B**) regioisomer **3b’**. (**C**) regioisomer **3c**, and (**D**) regioisomer **4b,** in HL-60 cells.

Figure 7 shows the dose–response curves of the compounds that exhibited the most favorable cytotoxic profile (regioisomers **3a**, **3b’**, **3c**, and **4b**). Increasing concentrations of each derivative were tested in order to reliably determine their potencies (IC_50_ values). A clear agreement was observed between the cell viability data presented in Table 1 and the IC_50_ values obtained. Compound **3a**, with 21.14% viability in HL-60 cells and an IC_50_ of 5.14 μM, together with compound **3c** (viability 25.42%, IC_50_ 4.0 μM), were the least potent derivatives. In contrast, compounds **3b’** and **4b** showed much lower viability (3.88% and 12.34%) and lower IC_50_ values (1.14 μM and 2.08 μM), indicating higher cytotoxicity. Nevertheless, all compounds are within the same order of magnitude in the micromolar range, suggesting that these differences are pharmacologically negligible.

Given the above, to evaluate the cell death mechanism responsible for the decreased viability of the HL-60 cell line, compounds **3a**, **3b’**, **3c**, and **4b** were selected. Therefore, flow cytometry (FC) assays were performed on the HL-60 cell line after treatment with the four aforementioned compounds. FC allows the detection of apoptotic or necrotic markers expressed by cells, depending on whether apoptosis or necrosis occurs. As was mentioned, activation of CB_1_ and CB_2_ receptors (cannabinoid agonism) promotes apoptosis and suppresses tumor cell growth [43,44,45,46,47,48]. Therefore, if FC analysis indicates that any of the tested compounds induce cell death through apoptosis, the result would more strongly suggest that the evaluated compound could exert its cytotoxic effect via an apoptotic cannabinoid-mediated mechanism. Annexin V/PI staining was employed to discriminate between apoptotic and necrotic cells, based on the externalization of phosphatidylserine and loss of membrane integrity, respectively, as previously described [57]. Double-positive labeling indicates cells in late-stage apoptosis or necroptosis.

Viable cells are negative for both Annexin V and PI fluorescence (Q3 population). In the early stages of apoptosis, cells stain with Annexin V but still exclude PI and therefore show a significant increase in Annexin V fluorescence (Q1 population). Likewise, in the late stages of apoptosis, cells display high Annexin V and PI fluorescence (Q2 population). Finally, cells with damaged membranes and/or in very late stages of apoptosis rapidly incorporate PI (Q4 population) [58].

**Figure 8:**
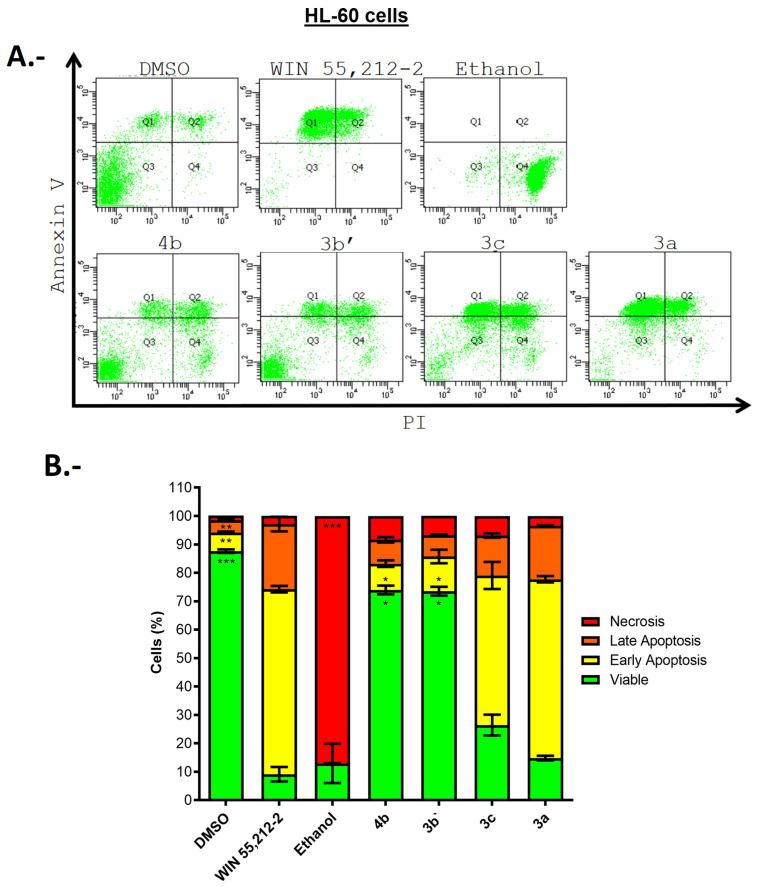
(**A**) Flow Cytometry (FC) dot plots of HL-60 cells after 72 h of incubation with DMSO, WIN-55,212-2 (5 µM), ethanol and compounds **3a**, **3b’**, **3c,** and **4b** (10 µM), which exhibited the best cytotoxic profile, as well as the lowest cytotoxic selectivity index values. (**B**) Bars graph show the results of selected compounds, the vehicle control (DMSO), the cannabinoid agonist WIN-55,212-2, and ethanol as a necrotic agent. Graph shows the average of results from three independent experiments (N = 3; mean ± SEM) and were analyzed using the non-parametric Kruskal–Wallis test for multiple comparisons. Significant differences in comparison to WIN-55,212-2 are indicated * *p* < 0.05, ** *p* < 0.01 and *** *p* < 0.001.

FC results for compounds **3a**, **3b’**, **3c**, and **4b**, together with the vehicle (DMSO) and the controls WIN-55,212-2 and ethanol, are presented in Figure 8A as dot plots and quantitatively summarized in Figure 8B as a bar graph. The dot plots allow discrimination of cell populations according to their apoptotic or necrotic status, while the bar graph quantifies the distribution of cells across viable, early apoptotic, and late apoptotic/necrotic categories.

From the analysis of the data presented in Figure 8, it can be observed that the vehicle solvent DMSO, used as a co-solvent to dissolve each compound (vehicle control), results in 87.6% viable cell population. Cells treated with ethanol were predominantly observed as a non-viable necrotic population, reaching 87%, making it a suitable necrotic control for the assay.

On the other hand, the well-known CB_1_/CB_2_ receptor agonist WIN-55,212-2 was evaluated at a concentration of 5 µM and showed a predominant early apoptosis population of 65%. This finding supports the suggestion that WIN-55,212-2 serves as a pro-apoptotic agent and reinforces the hypothesis that this compound could induce cell death through its cannabinoid agonist mechanism in CB_2_ receptor. The latter is based on all results, including reduced HL-60 viability and the observation that co-administration of increasing concentrations of the CB_2_ inverse agonist/antagonist AM630 decreases the toxicity of WIN-55,212-2 in these cells. Compounds **4b** and **3b’** resulted in a 73% viable cell population, indicating a low cytotoxic effect of these derivatives on the HL-60 cell line (bars graph, Figure 8B). Similarly, compound **3c** shows 52.6% non-viable cells due to early apoptosis, 14.1% due to late apoptosis, and a 26% viable cell population, suggesting a strong tendency toward cell death via early apoptosis. Furthermore, derivative **3a** induces 62.9% cell death by early apoptosis, 13.6% by late apoptosis and 11.2% of viable cells. Based on the above data, it can be determined that compounds **3a** and **3c** are similar to the well-known gold standard cannabinoid ligand WIN-55,212-2 in their apoptosis-inducing effect. Likewise, both regioisomers exhibit viability percentages comparable to WIN-55,212-2 but display better cytotoxic selectivity indexes, suggesting a potentially higher selectivity of these compounds for the CB_2_ receptor.

Results obtained for compounds **3a** and **3c** (Figure 8) indicate that early apoptosis is the predominant cell death mechanism that they induce in HL-60 cells. This could suggest, as previously mentioned, that the tested ligands would exert their cytotoxic effect through cannabinoid agonism at CB_2_ receptor. These findings are also consistent with our previously obtained results for the same derivatives in the cytotoxicity assay (MTT) on HL-60 cells (Figure 6 and Figure 7 and Table 1), where compounds **3a** and **3c** showed cell viabilities of 21.14% and 25.42% and IC_50_ values of 4.0 μM and 5.14 μM, respectively.

Compounds **3b’** and **4b** show that the percentage of viable cells observed by FC does not correlate with cytotoxicity assay data. In MTT experiments, these derivatives showed cell viabilities of 3.88% and 12.34% and IC_50_ values of 1.14 μM and 2.08 μM, respectively (Figure 6 and Figure 7 and Table 1). This discrepancy could be explained by a phenomenon in which both compounds may reduce viability at an extremely late stage (possibly through necrosis), which would account for the high percentage of viable cells observed in flow cytometry assays. Furthermore, the colorimetric method based on the use of tetrazolium salts cannot discriminate whether a compound has a cytotoxic or antiproliferative effect. This method depends on the metabolic capacity of intracellular NAD(P)H-dependent oxidoreductase enzymes, so there is a possibility that a compound could alter metabolic activity and, therefore, reduce the dye without affecting cell viability [59]. For instance, oxidative stress, which can be a consequence of an altered cellular redox state, can lead to reduced mitochondrial function and a subsequent decrease in formazan formation, thus underestimating cell viability [60]. Thus, based on the considerations mentioned above, it is possible that the discrepancies arise from late-stage apoptotic events or metabolic interference affecting the reduction of tetrazolium salts in the MTT assay. Therefore, further studies focusing on these aspects could provide additional insight into the precise mechanisms underlying the cytotoxic effects of compounds **3b’** and **4b**.

### 2.4. Docking Simulations

As mentioned in Section 2.1, we proposed a rational design to achieve CB_2_ cannabinoid activity. Accordingly, each scaffold borne by the main benzo [*d*]imidazole, such as the 4-methoxyphenyl core with a carbonyl or methylene group attached to the N1 atom; the aromatic rings (3-methoxybenzene, 3-pyridine, furan, and isoxazole) at position 2; and the chlorine atom, was designated according to a plan intended to enable specific interactions with particular amino acids.

Given that compounds **3a**, **3c**, **3b’**, and **4b** exhibited cytotoxic activity—where **3a** and **3c** were selectively cytotoxic according to cell viability assays in HL-60 cells, which overexpress the CB_2_ receptor, and showed significant induction of apoptosis as determined by FC analyses—these derivatives, together with WIN-55,212-2 and AM630 (used as controls for the simulations), as well as **3b’** and **4b** (cytotoxic according to cell viability assays but not by FC), were subjected to molecular docking studies in the CB_2_ receptor active site to analyze their molecular interactions and binding energies in the context of our obtained experimental background as potential CB_2_ agonists.

The best docking binding energies expressed in kcal/mol of each selected compound are shown in Table 2. Cryo-EM structure of the human CB_2_ receptor (PDB ID: 6PT0 [51]) was used. Calculations were done in the orthosteric binding site.

**Figure 9:**
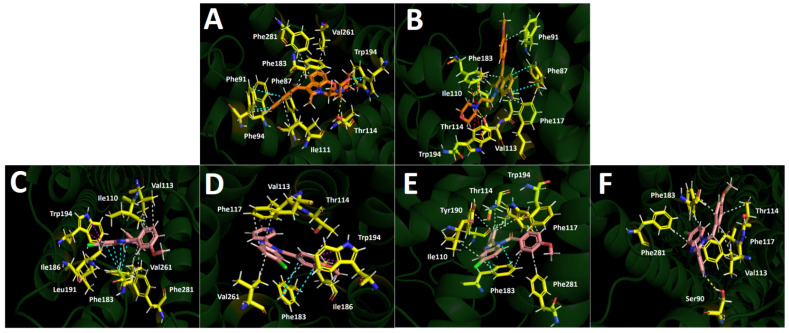
Predicted binding mode and predicted intermolecular interactions among the amino acids of the CB_2_ active site receptor and selected synthesized derivatives. Yellow dotted lines indicates hydrogen bond interactions, cyan dotted lines represents π-π interactions, magenta dotted lines represents T-shaped interactions, red dotted lines represents π-cation interactions. (**A**). *Aminoalkylindole* WIN-55,212-2; (**B**). *Aminoalkylindole* AM630; (**C**). Compound **3a**; (**D**). Compound **3b’**; (**E**). Compound **3c**; and (**F**). Compound **4b**.

The cannabinoid ligands WIN-55,212-2 and AM630 adopt highly similar orientations within the active site, with their indole cores overlapping and their morpholine moieties, as well as the naphthalene and 4-methoxyphenyl frameworks, aligned in the same direction. The observation for WIN-55,212-2 is consistent with the findings reported by Xing et al., with certain interactions resembling those previously described, such as π–π interactions with Phe94 and Trp194, as well as hydrophobic interactions with Phe87, Phe91, Phe94, Ile110, Phe183, Val261, and Phe281 [51]. In addition, our docking studies also showed a π-cation interaction between Trp194 and the protonated nitrogen atom of the morpholine moiety of WIN-55,212-2, as well as a hydrogen bond interaction with Thr114 (Figure 9A). Likewise, AM630 forms a hydrogen bond with Thr114, π–cation interactions with Trp194 and Phe183, and a π–π interaction with Phe87, as well as hydrophobic interactions with Phe91, Ile110, Val113, and Phe117 (Figure 9B).

Docking assays demonstrated that WIN-55,212-2 exhibited a better binding energy of –11.081 kcal/mol compared to AM630, which showed –10.681 kcal/mol. This is consistent with the inhibition constant values for the CB_2_ receptor previously reported for both ligands, with WIN-55,212-2 displaying a K_i_ ranging from 0.280 to 16.2 nM, and AM630 showing a K_i_ of 32.1 nM [19,55,56].

The synthesized regioisomers exhibited favorable energy profiles (Table 2), comparable to the value obtained for the cannabinoid ligand AM630 (–10.681 kcal/mol), particularly highlighting compounds **3a** and **3c**, which exhibited binding energies of –10.640 kcal/mol and –10.059 kcal/mol, respectively. These results suggest that the synthesized compounds may possess CB_2_ receptor affinities comparable to that of AM630.

Compounds **3b’** and **4b** are arranged in a similar manner within the active site, with both structures superimposed—a feature that may account for their lower binding energy values compared to regioisomers **3a** and **3c**—(Table 2). Furthermore, based on their docking descriptors of position and energy, and consequently their putative moderate affinity for CB_2_ receptor, these compounds are unlikely to exert significant apoptotic effects, as evidenced by the flow cytometry analyses (Figure 8). Compound **3b’** shows a π–π interaction with Phe183, a T-shape with Trp194, and several hydrophobic interactions with Val113, Thr114, Phe117, Phe183, Ile186, and Val261 (Figure 9D). In turn, compound **4b** exhibits a hydrogen bond interaction with Ser90, along with hydrophobic interactions involving Val113, Thr114, Phe117, Phe183, and Phe281 (Figure 9F).

**Figure 10:**
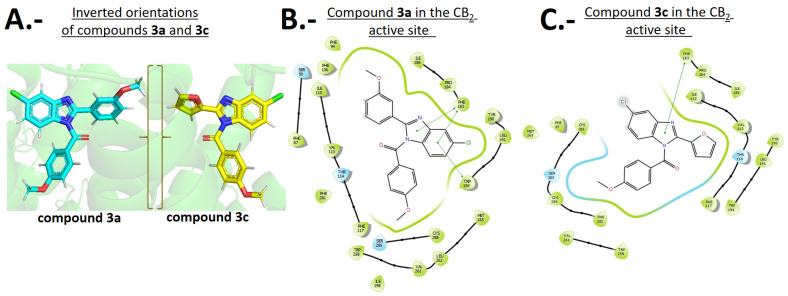
(**A**). Representation of compounds **3a** and **3c** with their respective benzo [*d*]imidazole cores inverted but sharing the same orientation within the CB_2_ receptor active site. (**B**). Two-dimensional diagram of CB_2_ active site and the main interactions with derivative **3a**. (**C**). Two-dimensional diagram of CB_2_ active site and the main interactions with derivative **3c**. The two-dimensional diagrams depict π–π and T-shaped interactions.

Derivatives **3a** and **3c**, which exhibit favorable binding energies (Table 2) and demonstrate cytotoxicity toward HL-60 cells as evidenced by viability assays and flow cytometry experiments, also display a certain similarity in their poses within the CB_2_ receptor active pocket. However, in this case, both compounds are also superimposed but exhibit inverted orientations in their benzo [*d*]imidazole cores, adopting opposite orientations as though they were ‘facing each other’. Figure 10A shows how compounds **3a** and **3c**, individually within the CB_2_ receptor active site, adopt the mentioned inverted orientations of their benzo [*d*]imidazole cores.

As mentioned in Section 2.1 (*Design Criteria for the Development of CB_2_ Ligands*), the benzo [*d*]imidazole moiety should establish interactions similar to those observed by Xing et al. with WIN-55,212-2 [51]. Accordingly, compound **3a** forms a π–π interaction between its benzo [*d*]imidazole core and Phe183, as well as a T-shaped interaction between the benzene ring of the benzo [*d*]imidazole and the indole framework of Trp194 (Figure 9C). Furthermore, **3a** engages in hydrophobic interactions through the 2-methoxyphenyl core with Ile110 and Val113, through its 4-methoxybenzoyl group with Val261 and Phe281, and also through the benzo [*d*]imidazole with Ile186 and Leu191 (Figure 9C). Thus, some of the predicted amino acids involving few interactions are effectively established. On the other hand, compound **3c** forms a π–π interaction between the benzo [*d*]imidazole moiety and Phe183; also engages in hydrophobic interactions through its benzo [*d*]imidazole with Ile110 and Phe183 and through the furan core with Thr114, Tyr190, and Trp194. Finally, it forms hydrophobic interactions through the 4-methoxybenzoyl group with Phe117, Phe183, Trp194, and Phe281 (Figure 9E). Hence, compound **3c** also exhibited interactions consistent with our design, such as those with Phe193, Trp194, and Phe117 residues.

Figure 10A shows compounds **3a** and **3c** with their respective benzo [*d*]imidazole cores adopting opposite orientations, as was mentioned, whereas Figure 10B,C depict the same derivatives in a two-dimensional diagram illustrating the main interactions and thereby summarizing key docking interactions.

Considering the docking data above, a comparative analysis shows that the synthesized compounds **3a** and **3c** adopt poses within the CB_2_ receptor active site that, although differing in orientation from the control ligands WIN-55,212-2 and AM630, maintain key interactions with residues such as Phe183 and Trp194. Moreover, compounds **3a** and **3c** exhibit favorable binding energies comparable to AM630, which correlates with their selective cytotoxicity and apoptotic activity in HL-60 cells. In contrast, compounds **3b’** and **4b**, although occupying similar positions within the binding pocket, display lower binding energies and fewer stabilizing interactions, consistent with their moderate cytotoxicity but lack of apoptosis induction. These results highlight how structural differences among the synthesized ligands translate into distinct receptor interactions and biological effects.

### 2.5. Molecular Dynamics (MD)

To corroborate the docking outcomes obtained for compounds **3a** and **3c** and to rigorously evaluate their potential as hits, molecular dynamics (MD) simulations were performed for both derivatives within the reported CB_2_ orthosteric binding site embedded in the cell membrane, for a simulation timescale of 500 ns.

**Figure 11:**
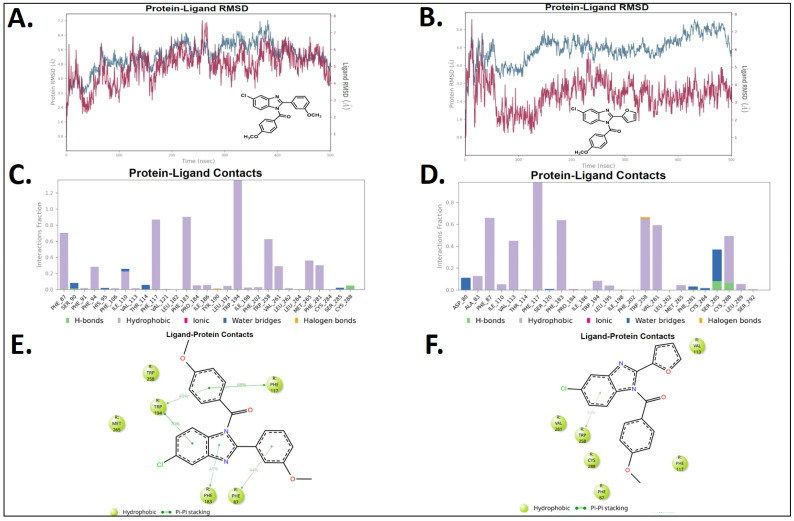
Results from molecular dynamics simulations (ensemble NPT, 1 atm, 310 K, 500 ns) for regioisomer **3a** (left) and regioisomer **3c** (right). (**A**,**B**). RMSD plots for CB_2_R in blue, and for **3a**–CB_2_R and **3c**–CB_2_R complexes in red. (**C**,**D**). Fraction of interactions per residue for **3a** and **3c**, respectively. Purple bars: hydrophobic interactions, green bars: H-bonds, magenta bars: ionic, blue bars: water-mediated H-bonds, orange bars: halogen bonds. Both compounds exhibit higher proportion of hydrophobic fraction of interactions. (**E**,**F**). Dwell times of the most relevant interactions for **3a** (left) and **3c** (right). Interactions per residue that persist for more than 30% of the total simulation time are displayed. π–π interactions and the extent of hydrophobic contacts of **3a** and **3c** potentially offer better stabilization within the CB_2_ receptor.

Figure 11A,B (blue traces) depict the Root Mean Square Deviation (RMSD) profiles of CB_2_ receptor in the assays conducted for compounds **3a** and **3c,** respectively.

The RMSD of the CB_2_ receptor during the MD simulation of **3a** remained within an acceptable fluctuation range over the 500 ns trajectory. However, for compound **3c**, the RMSD profile exhibits stability only during the initial ~50 ns, after which the protein undergoes considerable fluctuations until the end of the 500 ns simulation. The RMSD of the **3a**–CB_2_R complex (red trace, Figure 11A) remains consistent within an acceptable range, maintaining stability throughout the simulation, resulting in a more sustained dynamic behavior. In contrast, the **3c**–CB_2_R complex (red trace, Figure 11B) remains stable only during the initial nanoseconds, after which the RMSD increases significantly relative to the protein, reflecting substantial fluctuations.

Regarding the interaction profile per residue, regioisomer **3a** is stabilized primarily through hydrophobic interactions (purple bars, Figure 11C). The protein–ligand contact bars indicate that the key amino acids engaged during the MD simulations are Phe87, Phe94, Ile110, Phe117, Phe183, Trp194, Trp258, Val261, Met265, and Phe281. This is consistent with the previously discussed docking results, in which isomer **3a** forms a π–π contact with Phe183, a T-shaped arrangement with Trp194 (both involving the benzo [*d*]imidazole ring), and strong hydrophobic contacts with Ile110, Val261, and Phe281. Figure 11E also exhibits contacts with Phe87, Phe117, and Phe183 with favorable retention times of 44%, 68%, and 45%, respectively. Trp194 shows retention times of 49% and 70% due to its interactions with the 4-methoxybenzoyl group and the benzene ring of the benzo [*d*]imidazole core. Figure 11E Phe87 residue reveals a π–π stacking interaction with the 2-methoxyphenyl framework; this contact was not identified as contributing in the docking studies but is observed in the MD simulation. This contact is also represented in the bars shown in Figure 11C, albeit as a hydrophobic interaction.

On the other hand, Figure 11D shows the interaction fractions of compound **3c**, which, like derivative **3a**, is primarily stabilized through hydrophobic contacts (purple bars). The residues contributing most significantly are Phe87, Phe117, Phe183, Trp258, Val261, Ser285, and Cys288, which is consistent with our docking results (Figure 9), where several of these residues are directly involved in interactions with **3c**.

According to the MD dwell time results shown in Figure 11F, only residue Trp258 participates in a π–π stacking interaction with the benzo [*d*]imidazole scaffold (33% of the time). Moreover, in the bar graph shown in Figure 11D, a halogen bond can also be observed at a specific time point. This differs somewhat from the docking results, in which the only π–π interaction observed involved the amino acid Phe183, while all other contacts were predominantly hydrophobic. In this regard, docking and MD results show partial agreement; however, the RMSD profile of the **3c**–CB_2_R complex (Figure 11B) indicates that, although **3c** may retain some biological activity, it is relatively unstable and engages in unfavorable interactions within the CB_2_ active site over time.

The interactions observed in docking assays and MD simulations are in agreement with the well-established notion that hydrophobic interactions are essential for cannabinoid affinity [61]. In this context, both isomers exhibit stable binding profiles, supporting their potential as effective ligands for the CB_2_ receptor.

In order to estimate the relative affinities of compounds **3a** and **3c**, Molecular Mechanics–Generalized Born Surface Area (MM-GBSA) free energy calculations were carried out for each compound. To this end, we selected the most stable portion of the MD simulation, from 180 to 380 ns, extracting poses every 20 ns. The results are presented in Table 3. Compound **3a** showed a binding free energy value of –81.21 kcal/mol, while **3c** displayed –75.74 kcal/mol. This represents a 6.73% difference in the energy value, which could be attributed to the higher percentage of hydrophobic interactions achieved by **3a** as well as to its lower degree of solvation.

Finally, to systematize the available information, we present a structure–activity relationship (SAR) analysis for the series. Overall, comparison of active versus inactive analogues indicates that derivatives bearing a carbonyl at the nitrogen atom 1 (-N1) of the benzo [*d*]imidazole rings consistently outperform their methylene counterparts in inducing selective cytotoxicity and apoptosis in HL-60 cells (a CB_2_-high line), with **3a** and **3c** standing out. By contrast, analogues such as **4b** reduce the cell viability in MTT assays without confirming apoptosis at the evaluated time point. Likewise, 5-chloro substitution on the benzo [*d*]imidazole core is associated with more consistent biological profiles than 6-chloro substitution: compounds such as **3b’** (6-Cl) retain potency in HL-60 cytotoxicity assays, yet do not fully reproduce the apoptotic signal. At position 2 of the benzo [*d*]imidazole frameworks, π-rich, planar fragments (e.g., 3-methoxyphenyl in **3a** and furan in **3c**) maximize π–π stacking and hydrophobic contacts within the CB_2_ cavity, correlating with greater HL-60/U-87 MG selectivity and apoptosis induction. In contrast, more polar heteroaryl systems (e.g., isoxazole) are associated with attenuated activity. These trends agree with docking and molecular dynamics models, which for the pro-apoptotic actives reveal dense networks of hydrophobic interactions and π–π stacking with aromatic residues in the CB_2_ binding pocket. In sum, the ability to establish hydrophobic and π–π interactions with the CB_2_ receptor, together with C-5 chlorination, N-1 acylation, and aryl substituents at C-2 of the benzo [*d*]imidazole core, emerges as a strong predictor of pro-apoptotic activity across this series.

## 3. Materials and Methods

### 3.1. Procedure for the Synthesis of 6-Chloro-2-aryl-1H-benzo [d]imidazoles ***2***(***a-c***)

To a solution of 4-chlorobenzene-1,2-diamine (**1**) (330 mg, 2.1 mmol) in *N*,*N*-dimethylformamide (DMF) (10 mL) in a reaction flask under aerobic conditions and magnetic stirring, magnesium chloride hexahydrate (MgCl_2_·6H_2_O) (47.0 mg, 0.23 mmol, 10 mol%) was added, and the mixture was heated to 50 °C. Subsequently, 3.5 mmol of the corresponding aldehydes: 3-methoxybenzaldehyde, 3-pyridinecarboxaldehyde or 2-furaldehyde were slowly added dropwise. After the addition, the reaction was stirred at room temperature for 24 h. Later, the reaction mixture was poured into water and extracted with three portions of ethyl acetate. The organic portions were dried with anhydrous Na_2_SO_4_, filtered and then removed using a rotary evaporator. The solid residue was purified by column chromatography on silica gel with a mixture of ethyl acetate/ether as eluents in varying proportions as appropriate for each reaction. The procedure yield compounds **2a**, **2b,** and **2c** [62,63,64].

### 3.2. Procedure for the Synthesis of 3-(5-Chloro-1H-benzo [d]imidazol-2-yl)isoxazole (***2d***)

To synthesize intermediate 3-(5-chloro-1*H*-benzo [*d*]imidazol-2-yl)isoxazole (**2d**), isoxazole-3-carboxylic acid was used. 4-Chlorobenzene-1,2-diamine (**1**) (290 mg, 2.09 mmol) was added to a reaction flask containing polyphosphoric acid (PPA) under aerobic conditions. Reaction mixture was heated to 150 °C and stirred for 10 min. Subsequently, isoxazole-3-carboxylic acid (290 mg, 2.09 mmol) was also added, and the reaction was left for 3 h. Later, 30 mL of 1 M sodium hydroxide (NaOH) solution were added, followed by sodium bicarbonate to completely neutralize the PPA, reaching approximately pH = 8. Finally, the aqueous solution was extracted with three portions of ethyl acetate, dried with anhydrous Na_2_SO_4_ filtered and then removed using a rotary evaporator. The solid residue was purified by column chromatography on silica gel with a mixture of ethyl acetate/ether (40%/60%) as eluent.

### 3.3. Procedure for the Synthesis of (5 or 6)-(Chloro)-2-(aryl)-1H-benzo [d]imidazol-1-yl)(4-methoxyphenyl)methanone ***3***(***a-d***) and ***3***(***a’-d’***)

To a solution of the corresponding 6-chloro-2-aryl-1*H*-benzo [*d*]imidazoles **2**(**a-d**) (125 mg, 0.57 mmol) and sodium hydride (NaH) (1.4 mmol) in dry THF (20 mL) under anaerobic conditions (N_2_) and magnetic stirring was added dropwise 4-methoxybenzoyl chloride (194 mg; 154 µL; 1,14 mmol). After addition, the reaction was stirred at room temperature for 30 min. Then, the remaining NaH was removed by filtration, and the filtrate was poured over 100 mL of water and subsequently extracted with ethyl acetate. The organic portions were dried with anhydrous Na_2_SO_4_, filtered and concentrated under reduced pressure in a rotary evaporator. The residue was purified by thin-layer chromatography with 20% ethyl acetate and 80% hexane as eluent to yield target regioisomers **3**(**a-d**) and **3**(**a’-d’**).

### 3.4. Cell Cultures

All cell lines employed in our study are fully documented in the American Type Culture Collection (ATCC) database, including details on origin, morphology, culture conditions, recommended medium, markers and other relevant characteristics. The ATCC catalog numbers for the cell lines used are as follows: HL60 [ATCC CCL-240], U87MG [ATCC HTB-14], HEK 293 [ATCC CRL-1573]. Human embryonic kidney 293 (HEK293), neoplastic glioblastoma cells (U87MG), and acute promyelocytic leukemia cells (HL-60) were maintained at 5% CO_2_ and 37 °C in DMEM, EMEM and RPMI medium, respectively, containing 10% Fetal Bovine Serum and 100 U/mL penicillin and 100 mg/mL streptomycin.

### 3.5. Cell Viability: [MTT-(3-(4,5-dimethylthiazol-2-yl)-2,5-diphenyltetrazolium bromide)-formazan]

HEK293, U87MG, and HL-60 cells were first quantified to determine the exact number to be used in the MTT cell viability assays, ensuring that absorbance values remained below 1 unit in accordance with Beer-Lambert [65].

HEK293, U87MG and HL-60 cell lines were seed in a flat-bottom 96-wells plate at a density of 10,000 cells per well. Cells were then incubated with WIN-55,212-2 and AM630 at various concentrations (0.001 μM to 10 μM or 20 μM in case of HL-60 cells) in 200 μL of respective culture medium supplemented with 10% fetal bovine serum (FBS), at 37 °C for 72 h. Subsequently, 20 μL of 5 mg/mL MTT solution was added to each well, and cells were incubated at 37 °C for 4 h. The resulting formazan crystals were then solubilized with 10% sodium dodecyl sulfate (SDS) in 0.1 mM HCl, followed by overnight incubation at 37 °C. Untreated cells were used as the control, 0.1% of dimethyl sulfoxide (DMSO) as the vehicle control, and 0.2% of Triton X-100 as a positive control for cell death. IC_50_ toxicity value of WIN-55,212-2 in HL-60 cell line were determined.

Competition assay between WIN-55,212-2 and AM630 in HL-60 cells was conducted under the same conditions described in the previous paragraph. Therefore, WIN-55,212-2 was assayed in the presence of increasing concentrations of the CB_2_ inverse agonist/antagonist AM630 (0 μM to 20 μM), while WIN-55,212-2 was kept at a fixed concentration of 2.0 μM, based on the previously determined IC_50_. The stimulation with both CB ligands was performed simultaneously.

Cell viability assays on HEK293, U87MG and HL-60 cell lines of fourteen synthesized regioisomers were performed (Table 1). All compounds were incubated at 10 μM in 200 μL of culture medium supplemented with 10% FBS, in a flat-bottom 96-well plate at a density of 10,000 cells per well, at 37 °C for 72 h. Next, 20 μL of a 5 mg/mL MTT solution was added, and the cells were incubated at 37 °C for 4 h. Then, they were solubilized with 10% sodium dodecyl sulfate (SDS) in 0.1 mM HCl and incubated overnight at 37 °C. Formazan absorbance of each well was measured using the EPOCH microplate reader (Biotek Instruments Inc., Winooski, VT, USA) at a wavelength of 570 nm.

### 3.6. Flow Cytometry (FC) Experiments

Flow cytometry analysis was carried out to determine the mechanism of cell death associated with synthesized regioisomers. 3.0 × 10^5^ HL-60 cells were seeded in 24-well tissue culture plates and treated with 0.1% of dimethyl sulfoxide (DMSO) as vehicle control, WIN-55,212-2 as apoptosis positive control, the 4 selected synthesized regioisomers or untreated for 72 h. Then the cells were collected, one sample was treated with cold ethanol for 2 min. to permeabilize the membrane (positive control for necrosis), and centrifuged 300× *g* for 5 min. Next, cells were labeled using the Annexin V-FITC apoptosis staining (Thermo Fisher Scientific, Waltham, MA, USA) and propidium iodide (Ambeed Inc., Buffalo Grove, IL, USA) according to the manufacturer’s instructions. This was done by resuspending the pellets of cells in 100 μL of binding buffer with 10 μL of Annexin V-FITC and 0.1 μ/mL of propidium iodide in the dark for 15 min at room temperature. Then 400 μL of binding buffer were added to dilute the staining. Flow cytometry analysis was performed using a BD FACSCanto II (Becton, Dickinson and Company (BD), Franklin Lakes, NJ, USA) flow cytometer within one hour.

### 3.7. Molecular Docking Experiments

Docking simulations were performed for regioisomers **3a**, **3b’**, **3c**, **4b**, WIN-55,212-2 and AM630. Energetic minimization of each molecule was carried out using the LigPrep tool in the program Maestro Schrodinger suite v.11.8 (Schrödinger, LLC, New York, NY, USA) [66]. Cannabinoid receptor type 2 structure obtained from cryo-electron microscopy [CB_2_ receptor PDBID: 6PT0] [51], were obtained from the Protein Data Bank RCSB-PDB [67]. Receptor optimization was performed using the Protein Preparation Wizard available in Maestro Schrodinger suite v.11.8 (Schrödinger, LLC, New York, NY, USA) software. Water molecules (if applicable) were removed from the protein active site (orthosteric site). Appropriate ionization states for acid and basic amino acid residues, as well as polar hydrogen atoms, were considered at physiological pH = 7.4. The enclosing box was configured as a cube with 26 Å length, and the OPLS3e force field was employed for protein energy minimization. The centroid of the selected residue was determined based on the putative orthosteric active site of the CB_2_ receptor and its known catalytic amino acids, where the orthosteric ligand WIN-55,212-2 is positioned. The Glide Induced Fit Docking protocol has been used for the final couplings [68]. Compounds were punctuated by the Glide scoring function in the extra-precision mode (Glide XP; Schrödinger, LLC, New York, NY, USA) [69,70] and were filtered on the basis of the best scores and best RMS values (less than 1 unit as a cutting criterion) in order to obtain the potential intermolecular interactions between compounds and the receptor, as well as the binding mode and docking descriptors.

### 3.8. Molecular Dynamic (MD) Experiments

Molecular dynamics (MD) simulations of compounds **3a** and **3c** were performed using the Desmond module of the Schrödinger Maestro Suite v.11.8 (Schrödinger, LLC, New York, NY, USA). The starting point for each simulation was the best docking pose of each compound. Simulations were carried out for 500 ns in an NPT ensemble within a POPE membrane and TIP3P solvent environment, employing the OPLS3e force field. The analyses included interaction residence times, root-mean-square deviation (RMSD), total ligand–protein contacts, and solvent-accessible surface area (SASA).

## 4. Conclusions

We reported the synthesis of novel (5/6-chloro-2-aryl-1*H*-benzo [*d*]imidazol-1-yl)(4-methoxyphenyl)methanone and 5/6-chloro-1-(4-methoxybenzyl)-2-aryl-1*H*-benzo [*d*]imidazole regioisomers as CB_2_ ligands. Each molecule was exhaustively characterized by nuclear magnetic resonance spectroscopy to determine the exact position of the chlorine atom within the benzo [*d*]imidazole core. Subsequently, in order to evaluate their potential as CB_2_ agonists, based on multiple studies demonstrating that cannabinoid agonists induce cell death via an apoptotic mechanism, cell viability assays were conducted using three distinct cell lines: HEK293 cells, which express both cannabinoid receptors at negligible levels; U-87 MG cells, which highly express the CB_1_ receptor; and HL-60 cells, which exclusively express CB_2_ receptors. Compounds **3a**, **3b’**, **3c**, and **4b** selectively reduce HL-60 cell viability compared to HEK293 and U-87 MG cells. This observation may suggest agonist activity of these compounds at the CB_2_ receptor, given that the known agonist WIN-55,212-2 also induces toxicity in HL-60 cells. Moreover, the cytotoxic effect of WIN-55,212-2 was reversed when co-administered with increasing concentrations of the CB_2_ antagonist AM630, further supporting the hypothesis that the cytotoxic effect is mediated by cannabinoid receptor agonism. Furthermore, these benzo [*d*]imidazole isomers were subjected to flow cytometry experiments, in which compounds **3a** and **3c** were found to induce apoptosis in HL-60 cells, comparable to the effect of WIN-55,212-2. This finding once again suggests that both compounds could act as CB_2_ agonists according to our proposed indirect functional determination standards for cannabinoid agonism. In order to gain insight into the possible binding modes and to determine whether these modes are similar in energy and interaction descriptors to those of the well-known cannabinoid ligands WIN-55,212-2 and AM630, docking assays and molecular dynamics simulations were performed. The analyses revealed that their binding to the CB2 receptor is predominantly driven by π–π interactions and, most notably, by hydrophobic contacts, a pattern commonly observed for cannabinoid ligands in this type of system. Furthermore, MD results indicate greater stability for **3a** compared to **3c**. Since **3a** and **3c** possess a carbonyl group, whereas only derivative **4b** has a methylene group between the benzo [*d*]imidazole ring and the 4-methoxyphenyl group, it is suggested that the carbonyl group provides better results for this sort of compounds. Therefore, based on the biological background, docking studies, and MD results, our data support that compounds **3a** and **3c** exert selective cytotoxicity against HL-60 cells via a cannabinoid agonist-mediated apoptotic mechanism, as determined by our plausible indirect methodological approach.

Finally, although further experiments are required to confirm the agonistic nature of regioisomers **3a** and **3c**, in order to obtain accurate and precise results, we strongly believe that our proposed method provides a straightforward approach for rapidly assessing the cannabinoid agonist activity of new compounds, with the added advantage of yielding information that can guide the subsequent implementation of conventional assays for precise agonist activity determination.

## Figures and Tables

**Table 1:** Average cell viability percentage of the synthesized derivatives and the cannabinoid agonist WIN-55,212-2 in HEK293, U-87 MG, and HL-60 cells, used as models for cannabinoid-mediated cytotoxicity. The cytotoxic selectivity index values % HL-60/% U-87MG (CB_2_/CB_1_) are presented. The synthesized regioisomers were tested at 10 µM, while WIN-55,212-2 was tested at a concentration of 5 µM **^a^**.

Compound	Cell Viability Percentage (%) HEK293	Cell Viability Percentage (%) U-87MG	Cell Viability Percentage (%) HL-60	Selectivity Index% Cytotoxicity HL-60/% Cytotoxicity U-87MG
**3a**	60.87 ± 12	72.37 ± 20	21.14 ± 6	0.29
**3a’**	5.38 ± 13	6.71 ± 3	44.29 ± 11	6.6
**3b**	72.71 ± 24	84.78 ± 26	40.94 ± 16	0.48
**3b’**	74.71 ± 19	78.94 ± 13	3.88 ± 6	0.05
**3c**	84.44 ± 15	91.29 ± 28	25.42 ± 7	0.28
**3c’**	63.41 ± 30	73.91 ± 11	43.04 ± 14	0.58
**3d**	75.39 ± 30	68.23 ± 14	94.88 ± 20	1.39
**3d’**	71.71 ± 23	95.21 ± 23	95.58 ± 23	1.00
**4b**	103.12 ± 21	101.6 ± 27	12.34 ± 3	0.12
**4b’**	77.7 ± 34	93.93 ± 25	77.9 ± 11	0.83
**4c**	115.07 ± 22	92.94 ± 16	54.07 ± 28	0.58
**4c’**	95.5 ± 32	105.92 ± 28	92.43 ± 21	0.87
**4d**	105.7 ± 45	118.86 ± 33	68.8 ± 27	0.58
**4d’**	77.66 ± 19	92.87 ± 12	87.98 ± 23	0.95
**WIN-55,212-2**	89.6 ± 23	36.21 ± 12	12.21 ± 4	0.34

**^a^** Data are expressed as means of *n* = 3 independent experiments.

**Table 2:** Binding energies obtained from docking experiments of selected compounds, as well as the known cannabinoid ligand WIN-55,212-2 and AM630 over cannabinoid type 2 receptor (CB_2_ receptor).

Compound	Binding Energy (kcal/mol)
**3a**	−10.640
**3c**	−10.059
**3b’**	−9.632
**4b**	−9.822
**AM630**	−10.681
**WIN-55,212-2**	−11.081

**Table 3:** MM-GBSA binding free energy values for compounds **3a** and **3c**.

Compound 3a		Compound 3c	
Time (ns)	Energy (kcal/mol)	Time (ns)	Energy (kcal/mol)
**220**	−86.23	**180**	−73.89
**240**	−78.15	**200**	−75.46
**260**	−77.30	**220**	−78.51
**280**	−81.65	**240**	−74.00
**300**	−78.49	**260**	−85.65
**320**	−84.81	**280**	−74.22
**340**	−78.02	**300**	−74.70
**360**	−87.13	**320**	−74.32
**380**	−79.08	**340**	−70.93
**Average**	−81.21	**Average**	−75.74
**Std. Dev.**	3.88	**Std. Dev.**	4.20

## Data Availability

The original contributions presented in this study are included in the article/Supplementary Materials. Further inquiries can be directed to the corresponding authors.

## Supplementary Materials

The following supporting information can be downloaded at: https://www.mdpi.com/article/10.3390/ph18111599/s1. References [51,62,63,64,65,66,67,68,69,70] are cited in Supplementary Materials.

## References

[1] Lu H.-C., Mackie K. (2016). An Introduction to the Endogenous Cannabinoid System. Biol. Psychiatry.

[2] Matsuda L.A., Lolait S.J., Brownstein M.J., Young A.C., Bonner T.I. (1990). Structure of a Cannabinoid Receptor and Functional Expression of the Cloned cDNA. Nature.

[3] Munro S., Thomas K.L., Abu-Shaar M. (1993). Molecular Characterization of a Peripheral Receptor for Cannabinoids. Nature.

[4] Van Sickle M.D., Duncan M., Kingsley P.J., Mouihate A., Urbani P., Mackie K., Stella N., Makriyannis A., Piomelli D., Davison J.S. (2005). Identification and Functional Characterization of Brainstem Cannabinoid CB2 Receptors. Science.

[5] Palmer S.L., Thakur G.A., Makriyannis A. (2002). Cannabinergic Ligands. Chem. Phys. Lipids.

[6] Mackie K. (2006). Cannabinoid Receptors as Therapeutic Targets. Annu. Rev. Pharmacol. Toxicol..

[7] Lambert D.M., Fowler C.J. (2005). The Endocannabinoid System: Drug Targets, Lead Compounds, and Potential Therapeutic Applications. J. Med. Chem..

[8] Pertwee R.G. (2006). The Pharmacology of Cannabinoid Receptors and Their Ligands: An Overview. Int. J. Obes..

[9] Cassano T., Calcagnini S., Pace L., De Marco F., Romano A., Gaetani S. (2017). Cannabinoid Receptor 2 Signaling in Neurodegenerative Disorders: From Pathogenesis to a Promising Therapeutic Target. Front. Neurosci..

[10] Fonseca B., Teixeira N., Correia-da-Silva G. (2017). Cannabinoids as Modulators of Cell Death: Clinical Applications and Future Directions. Rev. Physiol. Biochem. Pharmacol..

[11] Palazuelos J., Aguado T., Egia A., Mechoulam R., Guzmán M., Galve-Roperh I. (2006). Non-Psychoactive CB2 Cannabinoid Agonists Stimulate Neural Progenitor Proliferation. FASEB J..

[12] Malan T.P., Ibrahim M.M., Lai J., Vanderah T.W., Makriyannis A., Porreca F. (2003). CB2 Cannabinoid Receptor Agonists: Pain Relief without Psychoactive Effects?. Curr. Opin. Pharmacol..

[13] Fernández-Ruiz J., Romero J., Velasco G., Tolón R.M., Ramos J.A., Guzmán M. (2007). Cannabinoid CB2 Receptor: A New Target for Controlling Neural Cell Survival?. Trends Pharmacol. Sci..

[14] Ge H., Ji B., Fang J., Wang J., Li J., Wang J. (2023). Discovery of Potent and Selective CB2 Agonists Utilizing a Function-Based Computational Screening Protocol. ACS Chem. Neurosci..

[15] Gioé-Gallo C., Ortigueira S., Prieto-Díaz R., Contino M., Azuaje J., Perrone M.G., Riganti C., Alberga D., Mangiatordi G.F., Andújar-Arias A. (2025). Conformational Restriction of Designer Drugs Reveals Subtype-Selective and Biased CB2 Agonists with Neuroprotective Effects. J. Med. Chem..

[16] Yang W., Sun H., Ji J., Chen H., Cheng J., Hu Z., Gong X., Liu Q., Peng S., Suo J. (2025). Identification of Cannabigerol-Derived Dual CB2 Receptor Agonists and TRPM8 Antagonists with Anti-Inflammatory and Analgesic Activities. J. Med. Chem..

[17] Aghazadeh Tabrizi M., Baraldi P.G., Borea P.A., Varani K. (2016). Medicinal Chemistry, Pharmacology, and Potential Therapeutic Benefits of Cannabinoid CB2 Receptor Agonists. Chem. Rev..

[18] Huffman J.W., Yu S., Showalter V., Abood M.E., Wiley J.L., Compton D.R., Martin B.R., Bramblett R.D., Reggio P.H. (1996). Synthesis and Pharmacology of a Very Potent Cannabinoid Lacking a Phenolic Hydroxyl with High Affinity for the CB2 Receptor. J. Med. Chem..

[19] Ross R.A., Brockie H.C., Stevenson L.A., Murphy V.L., Templeton F., Makriyannis A., Pertwee R.G. (1999). Agonist-Inverse Agonist Characterization at CB1 and CB2 Cannabinoid Receptors of L759633, L759656, and AM630. Br. J. Pharmacol..

[20] Smoum R., Baraghithy S., Chourasia M., Breuer A., Mussai N., Attar-Namdar M., Kogan N.M., Raphael B., Bolognini D., Cascio M.G. (2015). CB2 Cannabinoid Receptor Agonist Enantiomers HU-433 and HU-308: An Inverse Relationship between Binding Affinity and Biological Potency. Proc. Natl. Acad. Sci. USA.

[21] Marini P., Cascio M.-G., King A., Pertwee R.G., Ross R.A. (2013). Characterization of Cannabinoid Receptor Ligands in Tissues Natively Expressing Cannabinoid CB2 Receptors. Br. J. Pharmacol..

[22] Aung M.M., Griffin G., Huffman J.W., Wu M., Keel C., Yang B., Showalter V.M., Abood M.E., Martin B.R. (2000). Influence of the N-1 Alkyl Chain Length of Cannabimimetic Indoles upon CB(1) and CB(2) Receptor Binding. Drug Alcohol Depend..

[23] Ibrahim M.M., Deng H., Zvonok A., Cockayne D.A., Kwan J., Mata H.P., Vanderah T.W., Lai J., Porreca F., Makriyannis A. (2003). Activation of CB2 Cannabinoid Receptors by AM1241 Inhibits Experimental Neuropathic Pain: Pain Inhibition by Receptors Not Present in the CNS. Proc. Natl. Acad. Sci. USA.

[24] Huffman J.W., Marriott K.-S.C. (2008). Recent Advances in the Development of Selective Ligands for the Cannabinoid CB2 Receptor. Curr. Top. Med. Chem..

[25] Romero-Parra J., Mella-Raipán J., Palmieri V., Allarà M., Torres M.J., Pessoa-Mahana H., Iturriaga-Vásquez P., Escobar R., Faúndez M., Di Marzo V. (2016). Synthesis, Binding Assays, Cytotoxic Activity and Docking Studies of Benzimidazole and Benzothiophene Derivatives with Selective Affinity for the CB2 Cannabinoid Receptor. Eur. J. Med. Chem..

[26] Tebbe M.J., Spitzer W.A., Victor F., Miller S.C., Lee C.C., Sattelberg T.R., McKinney E., Tang J.C. (1997). Antirhino/Enteroviral Vinylacetylene Benzimidazoles: A Study of Their Activity and Oral Plasma Levels in Mice. J. Med. Chem..

[27] Trivedi R., De S.K., Gibbs R.A. (2006). A Convenient One-Pot Synthesis of 2-Substituted Benzimidazoles. J. Mol. Catal. A Chem..

[28] Jaya Preethi P., Karthikeyan E., Lohita M., Goutham Teja P., Subhash M., Shaheena P., Prashanth Y., Sai Nandhu K. (2015). Benzimidazole: An Important Scaffold in Drug Discovery. Asian J. Pharm. Tech..

[29] Guo Y., Hou X., Fang H. (2021). Recent Applications of Benzimidazole as a Privileged Scaffold in Drug Discovery. Mini Rev. Med. Chem..

[30] Nanda K.K., Henze D.A., Della Penna K., Desai R., Leitl M., Lemaire W., White R.B., Yeh S., Brouillette J.N., Hartman G.D. (2014). Benzimidazole CB2 Agonists: Design, Synthesis and SAR. Bioorg. Med. Chem. Lett..

[31] Watson C., Owen D.R., Harding D., Kon-I K., Lewis M.L., Mason H.J., Matsumizu M., Mukaiyama T., Rodriguez-Lens M., Shima A. (2011). Optimisation of a Novel Series of Selective CNS Penetrant CB(2) Agonists. Bioorg. Med. Chem. Lett..

[32] Pagé D., Balaux E., Boisvert L., Liu Z., Milburn C., Tremblay M., Wei Z., Woo S., Luo X., Cheng Y.-X. (2008). Novel Benzimidazole Derivatives as Selective CB2 Agonists. Bioorg. Med. Chem. Lett..

[33] Ryckmans T., Edwards M.P., Horne V.A., Correia A.M., Owen D.R., Thompson L.R., Tran I., Tutt M.F., Young T. (2009). Rapid Assessment of a Novel Series of Selective CB(2) Agonists Using Parallel Synthesis Protocols: A Lipophilic Efficiency (LipE) Analysis. Bioorg. Med. Chem. Lett..

[34] Gijsen H.J.M., De Cleyn M.A.J., Surkyn M., Van Lommen G.R.E., Verbist B.M.P., Nijsen M.J.M.A., Meert T., Wauwe J.V., Aerssens J. (2012). 5-Sulfonyl-Benzimidazoles as Selective CB2 Agonists-Part 2. Bioorg. Med. Chem. Lett..

[35] Tonelli M., Cichero E., Mahmoud A.M., Rabbito A., Tasso B., Fossa P., Ligresti A. (2018). Exploring the Effectiveness of Novel Benzimidazoles as CB2 Ligands: Synthesis, Biological Evaluation, Molecular Docking Studies and ADMET Prediction. MedChemComm.

[36] Nimczick M., Pemp D., Darras F.H., Chen X., Heilmann J., Decker M. (2014). Synthesis and Biological Evaluation of Bivalent Cannabinoid Receptor Ligands Based on hCB_2_R Selective Benzimidazoles Reveal Unexpected Intrinsic Properties. Bioorg. Med. Chem..

[37] Mella-Raipán J.A., Lagos C.F., Recabarren-Gajardo G., Espinosa-Bustos C., Romero-Parra J., Pessoa-Mahana H., Iturriaga-Vásquez P., Pessoa-Mahana C.D. (2013). Design, Synthesis, Binding and Docking-Based 3D-QSAR Studies of 2-Pyridylbenzimidazoles—A New Family of High Affinity CB1 Cannabinoid Ligands. Molecules.

[38] Romero-Parra J., Chung H., Tapia R.A., Faúndez M., Morales-Verdejo C., Lorca M., Lagos C.F., Di Marzo V., David Pessoa-Mahana C., Mella J. (2017). Combined CoMFA and CoMSIA 3D-QSAR Study of Benzimidazole and Benzothiophene Derivatives with Selective Affinity for the CB2 Cannabinoid Receptor. Eur. J. Pharm. Sci..

[39] Lorca M., Valdes Y., Chung H., Romero-Parra J., Pessoa-Mahana C.D., Mella J. (2019). Three-Dimensional Quantitative Structure-Activity Relationships (3D-QSAR) on a Series of Piperazine-Carboxamides Fatty Acid Amide Hydrolase (FAAH) Inhibitors as a Useful Tool for the Design of New Cannabinoid Ligands. Int. J. Mol. Sci..

[40] Cho A.Y.H., Chung H., Romero-Parra J., Kumar P., Allarà M., Ligresti A., Gallardo-Garrido C., Pessoa-Mahana H., Faúndez M., Pessoa-Mahana C.D. (2023). Motifs in Natural Products as Useful Scaffolds to Obtain Novel Benzo [d]Imidazole-Based Cannabinoid Type 2 (CB2) Receptor Agonists. Int. J. Mol. Sci..

[41] Balenga N.A., Martínez-Pinilla E., Kargl J., Schröder R., Peinhaupt M., Platzer W., Bálint Z., Zamarbide M., Dopeso-Reyes I.G., Ricobaraza A. (2014). Heteromerization of GPR55 and Cannabinoid CB2 Receptors Modulates Signalling. Br. J. Pharmacol..

[42] Ciaglia E., Torelli G., Pisanti S., Picardi P., D’Alessandro A., Laezza C., Malfitano A.M., Fiore D., Pagano Zottola A.C., Proto M.C. (2015). Cannabinoid Receptor CB1 Regulates STAT3 Activity and Its Expression Dictates the Responsiveness to SR141716 Treatment in Human Glioma Patients’ Cells. Oncotarget.

[43] Romero J., Lastres-Becker I., de Miguel R., Berrendero F., Ramos J.A., Fernández-Ruiz J. (2002). The Endogenous Cannabinoid System and the Basal Ganglia: Biochemical, Pharmacological, and Therapeutic Aspects. Pharmacol. Ther..

[44] Guzmán M. (2003). Cannabinoids: Potential Anticancer Agents. Nat. Rev. Cancer.

[45] Cudaback E., Marrs W., Moeller T., Stella N. (2010). The Expression Level of CB1 and CB2 Receptors Determines Their Efficacy at Inducing Apoptosis in Astrocytomas. PLoS ONE.

[46] Downer E., Boland B., Fogarty M., Campbell V. (2001). Δ9-Tetrahydrocannabinol Induces the Apoptotic Pathway in Cultured Cortical Neurones via Activation of the CB1 Receptor. Neuroreport.

[47] Maccarrone M., Finazzi-Agró A. (2003). The Endocannabinoid System, Anandamide and the Regulation of Mammalian Cell Apoptosis. Cell Death Differ..

[48] Campbell V.A. (2001). Tetrahydrocannabinol-Induced Apoptosis of Cultured Cortical Neurones Is Associated with Cytochrome *c* Release and Caspase-3 Activation. Neuropharmacology.

[49] Rieder S.A., Chauhan A., Singh U., Nagarkatti M., Nagarkatti P. (2010). Cannabinoid-Induced Apoptosis in Immune Cells as a Pathway to Immunosuppression. Immunobiology.

[50] Compton D.R., Gold L.H., Ward S.J., Balster R.L., Martin B.R. (1992). Aminoalkylindole Analogs: Cannabimimetic Activity of a Class of Compounds Structurally Distinct from Delta 9-Tetrahydrocannabinol. J. Pharmacol. Exp. Ther..

[51] Xing C., Zhuang Y., Xu T.-H., Feng Z., Zhou X.E., Chen M., Wang L., Meng X., Xue Y., Wang J. (2020). Cryo-EM Structure of the Human Cannabinoid Receptor CB2-Gi Signaling Complex. Cell.

[52] Elderfield R.C., McCarthy J.R. (1951). The Reaction of O-Phenylenediamines with Carbonyl Compounds. J. Am. Chem. Soc..

[53] Ghosh P., Subba R. (2015). MgCl_2_·6H_2_O Catalyzed Highly Efficient Synthesis of 2-Substituted-1*H*-Benzimidazoles. Tetrahedron Lett..

[54] Hein D., Alheim R.J., Leavitt J. (1957). The Use of Polyphosphoric Acid in the Synthesis of 2-Aryl-and 2-Alkyl-Substituted Benzimidazoles, Benzoxazoles and Benzothiazoles1. J. Am. Chem. Soc..

[55] Pertwee R.G. (2005). Pharmacological Actions of Cannabinoids. Cannabinoids.

[56] An D., Peigneur S., Hendrickx L.A., Tytgat J. (2020). Targeting Cannabinoid Receptors: Current Status and Prospects of Natural Products. Int. J. Mol. Sci..

[57] Darzynkiewicz Z., Bruno S., Del Bino G., Gorczyca W., Hotz M.A., Lassota P., Traganos F. (1992). Features of Apoptotic Cells Measured by Flow Cytometry. Cytometry.

[58] Pozarowski P., Grabarek J., Darzynkiewicz Z. (2004). Flow Cytometry of Apoptosis. Curr. Protoc. Cell Biol..

[59] Kepp O., Galluzzi L., Lipinski M., Yuan J., Kroemer G. (2011). Cell Death Assays for Drug Discovery. Nat. Rev. Drug Discov..

[60] Ghasemi M., Turnbull T., Sebastian S., Kempson I. (2021). The MTT Assay: Utility, Limitations, Pitfalls, and Interpretation in Bulk and Single-Cell Analysis. Int. J. Mol. Sci..

[61] Stasiulewicz A., Lesniak A., Bujalska-Zadrożny M., Pawiński T., Sulkowska J. (2023). Identification of novel CB2 ligands through virtual screening and in vitro evaluation. J. Chem. Inf. Model..

[62] Choudhary M.I. (2016). Synthesis and in Vitro α-Chymotrypsin Inhibitory Activity of 6-Chlorobenzimidazole Derivatives. Bioorg. Med. Chem..

[63] Jung M.H., Park J.M., Lee I.-Y.C., Ahn M. (2003). Synthesis of 2-(1-Methyl-1,2,5,6-Tetrahydropyridin-3-Yl)Benzimidazoles. J. Heterocycl. Chem..

[64] Pham E.C., Thi Le T.V., Truong T.N. (2022). Design, Synthesis, Bio-Evaluation, and in Silico Studies of Some N-Substituted 6-(Chloro/Nitro)-1H-Benzimidazole Derivatives as Antimicrobial and Anticancer Agents. RSC Adv..

[65] Mosorov V. (2017). The Lambert-Beer Law in Time Domain Form and Its Application. Appl. Radiat. Isot..

[66] (2018). Schrödinger Release 2018-2: Maestro.

[67] Rose P.W., Prlić A., Bi C., Bluhm W.F., Christie C.H., Dutta S., Green R.K., Goodsell D.S., Westbrook J.D., Woo J. (2015). The RCSB Protein Data Bank: Views of Structural Biology for Basic and Applied Research and Education. Nucleic Acids Res..

[68] Sherman W., Day T., Jacobson M.P., Friesner R.A., Farid R. (2006). Novel Procedure for Modeling Ligand/Receptor Induced Fit Effects. J. Med. Chem..

[69] Friesner R.A., Murphy R.B., Repasky M.P., Frye L.L., Greenwood J.R., Halgren T.A., Sanschagrin P.C., Mainz D.T. (2006). Extra Precision Glide: Docking and Scoring Incorporating a Model of Hydrophobic Enclosure for Protein-Ligand Complexes. J. Med. Chem..

[70] DeLano W. (2002). PyMol: An Open-Source Molecular Graphics Tool. https://mozart.gmb.bio.br/uploads/6/4/3/6/64362047/pymol.pdf#page=44.

