# Supplementary material for: Synthesis of Novel Chloro-Benzo [d]imidazole Regioisomers as Selective CB2 Receptor Agonists: Indirect Functional Evaluation and Molecular Insights
Source: Pharmaceuticals (Basel). 2025 Oct 22;18(11):1599. doi: 10.3390/ph18111599 (PMC12655022; doi:10.3390/ph18111599)
Supplement: Supplementary file 1 [file pharmaceuticals-18-01599-s001.zip › pharmaceuticals-3885908-supplementary.pdf]

Supplementary Material

Synthesis of Novel Chloro-Benzo[*d*]imidazole Regioisomers as Selective CB_2 Receptor Agonists: Indirect Functional Evaluation and Molecular Insights

Valeria Zuñiga Salazar[a], Renato Burgos Ravanal[b], Jonathan Soto-Flores, Gianfranco Sabadini[b], José Vicente González[b], Jaime Mella[c]*, Javier Romero-Parra [a]*.

[a] *Organic Chemistry and Physical Chemistry Department, Faculty of Chemical and Pharmaceutical Sciences, Universidad de Chile, Olivos 1007, Santiago 7820436, Chile; javier.romero@ciq.uchile.cl*

[b] *Pharmacy Department, Faculty of Chemistry, Pontificia Universidad Católica de Chile, Vicuña Mackenna 4860, Santiago 7820436, Chile;*

[c] *Institute of Chemistry and Biochemistry, Faculty of Sciences, Universidad de Valparaíso, Valparaíso 2360102, Chile; Faculty of Pharmacy, Chilean Pharmacopoeia Research Center, Universidad de Valparaíso, Valparaíso 2360102, Chile.*

Table of contents

General information……………………………………………………………………………………………….4.

Chemistry……….4.

1) Procedure for the synthesis of 6-chloro-2-aryl-1*H*-benzo[*d*]imidazoles 2(a-c)…………………………4.

2) Procedure for the synthesis of 3-(5-chloro-1*H*-benzo[*d*]imidazol-2-yl)isoxazole (2d) ……………4.

- 5(6)-chloro-2-(3-methoxyphenyl)-1*H*-benzo[*d*]imidazole **(2a)**
- 5(6)-chloro-2-(pyridin-3-yl)-1*H*-benzo[*d*]imidazole **(2b)**
- 5(6)-chloro-2-(furan-2-yl)-1*H*-benzo[*d*]imidazole **(2c)**
- 3-(5(6)-chloro-1*H*-benzo[*d*]imidazol-2-yl)isoxazole **(2d)**

3) Procedure for the synthesis of (5 or 6)-(chloro)-2-(aryl)-1*H*-benzo[*d*]imidazol-1-yl)(4-methoxyphenyl)methanone 3(a-d) and 3(a'-d')……………………………………………………………6.

- (5-chloro-2-(3-methoxyphenyl)-1*H*-benzo[*d*]imidazol-1-yl)(4-methoxyphenyl)methanone **(3a)**
- (6-chloro-2-(3-methoxyphenyl)-1*H*-benzo[*d*]imidazol-1-yl)(4-methoxyphenyl)methanone **(3a')**
- (5-chloro-2-(pyridin-3-yl)-1*H*-benzo[*d*]imidazol-1-yl)(4-methoxyphenyl)methanone **(3b)**
- (6-chloro-2-(pyridin-3-yl)-1*H*-benzo[*d*]imidazol-1-yl)(4-methoxyphenyl)methanone **(3b')**
- (5-chloro-2-(furan-2-yl)-1*H*-benzo[*d*]imidazol-1-yl)(4-methoxyphenyl)methanone **(3c)**
- (6-chloro-2-(furan-2-yl)-1*H*-benzo[*d*]imidazol-1-yl)(4-methoxyphenyl)methanone **(3c')**
- (5-chloro-2-(isoxazol-3-yl)-1*H*-benzo[*d*]imidazol-1-yl)(4-methoxyphenyl)methanone **(3d)**
- (6-chloro-2-(isoxazol-3-yl)-1*H*-benzo[*d*]imidazol-1-yl)(4-methoxyphenyl)methanone **(3d')**

4) Procedure for the synthesis of (5 or 6)-chloro-1-(4-methoxybenzyl)-2-aryl-1*H*-benzo[*d*]imidazole 4(a-d) and 4(a'-d')………………………………………………………………………10.

- 5-chloro-1-(4-methoxybenzyl)-2-(3-methoxyphenyl)-1*H*-benzo[*d*]imidazolemethanone **(4a)**
- 6-chloro-1-(4-methoxybenzyl)-2-(3-methoxyphenyl)-1*H*-benzo[*d*]imidazolemethanone **(4a')**
- 5-chloro-1-(4-methoxybenzyl)-2-(pyridin-3-yl)-1*H*-benzo[*d*]imidazole **(4b)**
- 6-chloro-1-(4-methoxybenzyl)-2-(pyridin-3-yl)-1*H*-benzo[*d*]imidazole **(4b')**
- 5-chloro-2-(furan-2-yl)-1-(4-methoxybenzyl)-1*H*-benzo[*d*]imidazole **(4c)**
- 6-chloro-2-(furan-2-yl)-1-(4-methoxybenzyl)-1*H*-benzo[*d*]imidazole **(4c')**
- 3-(5-chloro-1-(4-methoxybenzyl)-1*H*-benzo[*d*]imidazol-2-yl)isoxazole **(4d)**
- 3-(6-chloro-1-(4-methoxybenzyl)-1*H*-benzo[*d*]imidazol-2-yl)isoxazole **(4d')**

NMR spectra of compounds…………………………………………………………………………………15.

Biological experiments……………………………………………………………………………………………………..64.

- Cell cultures.
- Cell viability: ([MTT - (3-(4,5-dimethylthiazol-2-yl)-2,5-diphenyltetrazolium bromide) - formazan].
- Flow cytometry (FC) experiments.

Molecular docking experiments………………………………………………………………………………………….65.

Molecular dynamics (MD) experiments……………………………………………………………………………..66.

General information

All starting materials and solvents were purchased from commercial suppliers and used without further purification. Solvents were dried by reflux over sodium overnight and freshly distilled before use. Reactions over nitrogen atmosphere were carried out filling the reaction apparatuses through a gas flow of the corresponding, commercially available gas and afterwards closing the filled reaction system with a gas filled balloon. Thin layer chromatography (TLC) was performed on silica gel on aluminum foils (1.5 × 5 cm) pre-coated (0.25 mm) with silica gel (Merck) with fluorescent indicator at 254 nm. For column chromatography silica gel 60 (particle size: 0.063 - 0.200 mm or 0.035 - 0.070 mm) was used. Compounds were visualized by exposure to UV light. Melting points were determined on a Electrothermal 9100 apparatus and are uncorrected. Nuclear magnetic resonance spectra were recorded on a Bruker AM-300 instrument using DMSO-d6 or $CDCl_3$ solutions containing tetramethylsilane as internal standard. Chemical shifts are expressed in parts per million (ppm) downfield from TMS, coupling constant values (*J*) are given in Hertz. Multiplicity are abbreviated as, s: singlet; brs: broad singlet d: doublet; t: triplet; q: quartet; p: quintet; m: multiplet; dd: doublet of doublet; and so on.

Chemistry:

1) Procedure for the synthesis of 6-chloro-2-aryl-1*H*-benzo[*d*]imidazoles 2(a-c)

To a solution of 4-chlorobenzene-1,2-diamine (**1**) (330 mg, 2.1 mmol) in *N*,*N*-dimethylformamide (DMF) (10 mL) in a reaction flask under aerobic conditions and magnetic stirring, magnesium chloride hexahydrate ($MgCl_2{\cdot}6H_2O$) (47.0mg, 0.23 mmol, 10 mol%) was added and the mixture was heated to 50°C. Subsequently, 3.5mmol of the corresponding aldehydes: 3-methoxybenzaldehyde, 3-pyridinecarboxaldehyde or 2-furaldehyde were slowly added dropwise. After addition, the reaction was stirred at room temperature for 24 h. Later, reaction mixture was poured into water and extracted with three portions of ethyl acetate. The organic portions were dried with anhydrous Na_2SO_4, filtered and then removed using a rotary evaporator. The solid residue was purified by column chromatography on silica gel with a mixture of ethyl acetate/ether as eluents in varying proportions as appropriate for each reaction. The procedure yield compounds **2a**, **2b** and **2c**

2) Procedure for the synthesis of 3-(5-chloro-1*H*-benzo[*d*]imidazol-2-yl)isoxazole (2d)

To synthesize intermediate 3-(5-chloro-1*H*-benzo[*d*]imidazol-2-yl)isoxazole (**2d**), isoxazole-3-carboxylic acid was used. 4-Chlorobenzene-1,2-diamine (**1**) (290mg, 2.09 mmol) was added to a reaction flask containing polyphosphoric acid (PPA) under aerobic conditions. Reaction mixture was heated to 150°C and stirred for 10 minutes. Subsequently, isoxazole-3-carboxylic acid (290 mg, 2.09 mmol) was also added and reaction was left for 3 h. Later, 30 mL of 1 M sodium hydroxide (NaOH) solution were added, followed by sodium bicarbonate to completely neutralize the PPA, reaching approximately pH = 8. Finally, the aqueous solution was extracted with three portions of ethyl acetate, dried with anhydrous Na_2SO_4, filtered and then removed using a rotary evaporator. The solid residue was purified by column chromatography on silica gel with a mixture of ethyl acetate/ether (40%/60%) as eluent.

5(6)-chloro-2-(3-methoxyphenyl)-1*H*-benzo[*d*]imidazole (2a):

Following the general procedure 1.

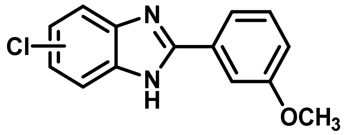

White solid. Yield = 61%.   mp: 139 -140 °C. (*)

^{1}H NMR (300 MHz, DMSO-d^6) δ ppm: 7.79 (brs, 1H), 7.76 (d, 1H, J_1 = 1.6 Hz), 7.66 (d, 1H, J_1 = 1.6 Hz), 7.60 (d, 1H, J_1 = 8.3 Hz), 7.45 (t, 1H, J_1 = 8.3 Hz), 7.22 (dd, 1H, J_1 = 8.5 Hz, J_2 = 1,6 Hz), 7.06 (dt, 1H, J_1 = 8.5 Hz, J_2 = 1.6 Hz), 3.85 (s, 3H).

^{13}C NMR (75.5 MHz, DMSO-d^6) δ ppm: 159.66, 152.64, 140.68, 137.91, 131.11, 130.13, 126.43, 122.35, 118.92, 116.18, 116.05, 114.99, 111.54, 55.29.

(*) Siddiqui, H., et al., *Synthesis and in vitro α-chymotrypsin inhibitory activity of 6 chlorobenzimidazole derivatives.* Bioorganic & Medicinal Chemistry, 2016. **24**(16): p. 3387-3395.

5(6)-chloro-2-(pyridin-3-yl)-1*H*-benzo[*d*]imidazole (2b):

Following the general procedure 1.

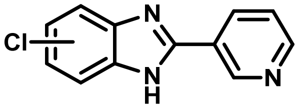

White solid. Yield = 30%.   mp: 145 – 147 °C. (*)

^{1}H NMR 13.30(brs, 1H), 9.34 (d, 1H, J_1 =1.6 Hz), 8.69 (dd, 1H, J_1 =4.8 Hz, J_2 =1.6 Hz), 8.48 (dt, 1H, J_1 = 8.0 Hz, J_2 = 1.6 Hz), 7.68 (d, 1H, J_1 = 1.8 Hz), 7.63 (d, 1H, J_1 = 8.6 Hz), 7.60 (dd, 1H, J_1 = 7.8 Hz, J_2 = 4.8 Hz), 7.23 (dd, 1H, J_1 = 8.6 Hz, J_2 = 1.98 Hz).

^{13}C NMR (75.5 MHz, DMSO-d^6) δ ppm: 150.83, 150.36, 147.66, 140.61, 137.91, 133.98, 126.83, 125.81, 124.07, 122.78, 116.38, 115.17.

* Jung, M.H., et al., *Synthesis of 2-(1-methyl-1, 2, 5, 6-tetrahydropyridin-3-yl) benzimidazoles.* Journal of heterocyclic chemistry, 2003. **40**(1): p. 37-44.

5(6)-chloro-2-(furan-2-yl)-1*H*-benzo[*d*]imidazole (2c):

Following the general procedure 1.

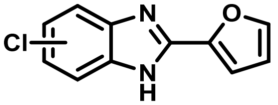

White solid. Yield = 58%.   mp: 200 – 202 °C. (*)

^{1}H NMR (300 MHz, DMSO-d^6) δ ppm: 13.12 (brs, 1H), 7.95 (d, 1H, J_1 = 2.2 Hz), 7.68-7.52, (m, 2H), 7.24-7.20 (m, 2H), 6.73 (dd, 1H, J_1 = 3.5 Hz, J_2 = 1.7 Hz).

^{13}C NMR (75.5 MHz, DMSO-d^6) δ ppm: 145.12, 144.99, 144.71, 142.50, 135.06, 126.25, 122.67, 119.99, 118.15, 112.43, 111.19.

* Pham, E.C., T.V.T. Le, and T.N. Truong, *Design, synthesis, bio-evaluation, and in silico studies of some N-substituted 6-(chloro/nitro)-1 H-benzimidazole derivatives as antimicrobial and anticancer agents.* RSC advances, 2022. **12**(33): p. 21621-21646.

3-(5(6)-chloro-1*H*-benzo[*d*]imidazol-2-yl)isoxazole (2d):

Following the general procedure 2.

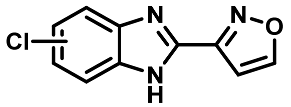

White solid. Yield = 66%. mp: 235.2 – 236.4 °C.

^{1}H NMR (300 MHz, DMSO-d^6) δ ppm: 9.17(d, 1H, J_1 = 1.7 Hz), 7.69 (d, 1H, J_1 = 2.0 Hz), 7.64 (d, 1H, J_1 = 8.6 Hz), 7.27 (dd, 1H, J_1 = 2.0 Hz), 7.22 (d, 1H, J_1 = 1.7 Hz).

^{13}C NMR (75.5 MHz, DMSO-d^6) δ ppm: 161.49, 154.59, 143.36, 140.06, 137.67, 127.38, 123.36, 116.99, 115.51, 104.04.

3) Procedure for the synthesis of (5 or 6)-(chloro)-2-(aryl)-1*H*-benzo[*d*]imidazol-1-yl)(4-methoxyphenyl)methanone 3(a-d) and 3(a'-d')

To a solution of the corresponding 6-chloro-2-aryl-1*H*-benzo[*d*]imidazoles **2(a-d)** (125 mg, 0.57 mmol) and sodium hydride (NaH) (1.4 mmol) in dry THF (20 mL) under anaerobic conditions (N_2) and magnetic stirring was added dropwise 4-methoxybenzoyl chloride (194 mg; 154 µL; 1,14 mmol). After addition, reaction was stirred at room temperature for 30 min. Then, the remaining NaH was removed by filtration and the filtrate was poured over 100 mL of water and subsequently extracted with ethyl acetate. The organic portions were dried with anhydrous Na_2SO_4, filtered and concentrated under reduced pressure in a rotary evaporator. The residue was purified by thin layer chromatography with 20% ethyl acetate and 80% hexane as eluent to yield target regioisomers **3(a-d)** and **3(a'-d')**.

(5-chloro-2-(3-methoxyphenyl)-1*H*-benzo[*d*]imidazol-1-yl)(4-methoxyphenyl)methanone (3a)

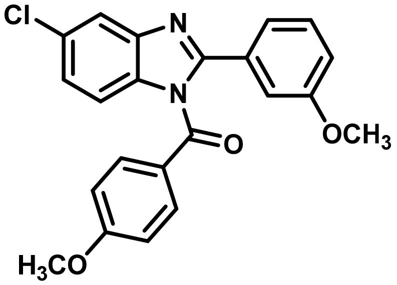

light brown solid. Yield = 53%. mp: 143.2-144.5°C.

^{1}H NMR (300 MHz, $CDCl_3$) δ ppm: 7.86 (d, 1H, J_1 = 2.0 Hz), 7.71(d, 2H, J_1 = 8.9 Hz), 7.32 (d, 1H, J_1 = 8.7 Hz), 7.28-7.22 (m, 3H), 7.20-7.25 (m, 1H), 6.93 (dd, 1H, J_1 = 2.3 Hz, J_1 = 1.4 Hz), 6.87(d, 2H, J_1 = 8.9 Hz), 3.87(s, 3H), 3.81(s, 3H).

^{13}C NMR (75.5 MHz, $CDCl_3$) δ ppm: 167.93, 164.92, 159.65, 154.47, 141.60, 135.81, 133.34, 131.30, 130.31, 129.70, 124.98, 124.77, 121.74, 121.07, 116.65, 114.49, 114.03, 113.00, 55.81, 55.52

IR: 1694 cm^{-1} (C=O), 1258 cm^{-1} (C-O)

(5-chloro-2-(pyridin-3-yl)-1*H*-benzo[*d*]imidazol-1-yl)(4-methoxyphenyl)methanone (3b)

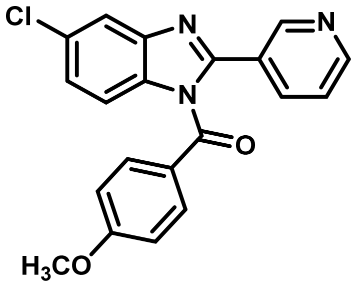

White solid. Yield = 51%. mp: 145.2 – 146.1°C.

^{1}H NMR (300 MHz, DMSO-d^{6}) δ ppm: 8.89 (d, 1H, J_1 = 1.6 Hz), 8.61 (dd, 1H, J_1 = 4.8 Hz, J_2 = 1.4 Hz), 7.98 (dt, 1H, J_1 = 8.0 Hz, J_2 = 1.9 Hz), 7.88 (t, 1H, J_1 = 2.3 Hz), 7.75 (d, 2H, J_1 = 8.9 Hz), 7.29-7.28 (m, 3H), 6.91 (d, 2H, J_1 = 8.9 Hz), 3.89 (s, 3H).

^{13}C NMR (75.5 MHz, $CDCl_3$) δ ppm: 167.43, 165.18, 152.31, 150.89, 149.84, 143.87, 136.40, 133.82, 133.51, 130.14, 126.59, 125.39, 124.29, 123.27, 120.35, 114.68, 113.94, 55.87.

IR: 1696 cm^{-1} (C=O), 1258 cm^{-1} (C-O)

(6-chloro-2-(pyridin-3-yl)-1*H*-benzo[*d*]imidazol-1-yl)(4-methoxyphenyl)methanone (3b')

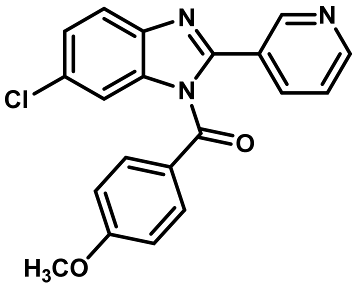

White solid. Yield = 49%. mp: 158.9-159.7°C.

^{1}H NMR (300 MHz, DMSO-d^{6}) δ ppm: 8.87 (a, 1H), 8.58 (d, 1H, J_1 = 3.9 Hz), 7.95 (dt, 1H, J_1 = 7.9 Hz, J_2 = 1.8 Hz), 7.79 (d, 1H, J_1 = 8.4 Hz), 7.73 (d, 2H, J_1 = 8.9 Hz), 7.40 (d, 1H, J_1 = 1.4 Hz), 7.36 (dd, 1H, J_1 = 8.5 Hz, J_2 = 1.9 Hz), 7.27 (m, 1H), 6.89 (d, 2H, J_1 = 8.9 Hz), 3.87 (s, 3H).

^{13}C NMR (75.5 MHz, $CDCl_3$) δ ppm: 167.39, 165.22, 151.75, 150.79, 149.77, 141.64, 136.31, 135.72, 133.51, 130.85, 126.61, 125.33, 124.17, 123.27, 121.32, 114.72, 113.27, 55.87.

IR: 1696 cm^{-1} (C=O), 1258 cm^{-1} (C-O)

(5-chloro-2-(furan-2-yl)-1*H*-benzo[*d*]imidazol-1-yl)(4-methoxyphenyl)methanone (3c)

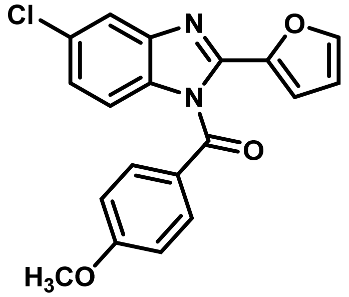

White solid. Yield = 51%. mp: 131.5 – 132.4°C.

^{1}H NMR (300 MHz, $CDCl_3$) δ ppm: 7.79 (d, 1H, J_1 = 1.1 Hz), 7.74 (d, 2H, J_1 = 8.9 Hz), 7.36 (d, 1H, J_1 = 1.0 Hz), 7.18 (qd, 2H, J_1 = 8.7 Hz, J_2 = 1.7 Hz), 7.05 (d, 1H, J_1 = 3.3 Hz), 6.91 (d, 2H, J_1 = 8.9 Hz), 6.43 (dd, 1H, J_1 = 3.4 Hz, J_2 = 1.7 Hz), 3.86 (s, 3H).

^{13}C NMR (75.5 MHz, $CDCl_3$) δ ppm: 167.30, 164.99, 145.52, 144.97, 143.90, 143.68, 133.20, 133.15, 129.79, 124.86, 124.58, 119.88, 114.51, 114.09, 113.34, 112.17, 55.81.

IR: 1711 cm^{-1} (C=O), 1271 cm^{-1} (C-O).

(6-chloro-2-(furan-2-yl)-1*H*-benzo[*d*]imidazol-1-yl)(4-methoxyphenyl)methanone (3c')

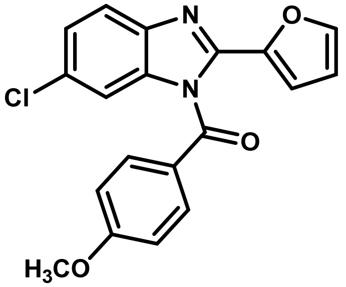

White solid. Yield = 49%. mp: 91.5-92.5°C.

^{1}H NMR (300 MHz, $CDCl_3$) δ ppm: 7.74 (m, 3H), 7.35 (d, 1H, J_1 = 1.1 Hz), 7.3 (td, 2H, J_1 = 7.6 Hz, J_2 = 1.9 Hz), 7.04 (d, 1H, J_1 = 3.4 Hz), 6.93 (d, 2H, J_1 = 8.9 Hz), 6.3 (d, 1H, J_1 = 4.5 Hz, J_2 = 1.7 Hz), 3.88 (s, 3H).

^{13}C NMR (75.5 MHz, $CDCl_3$) δ ppm: 167.28, 165.05, 145.08, 144.86, 144.02, 141.51, 135.13, 133.13, 130.27, 125.03, 124.61, 120.94, 114.57, 113.82, 112.69, 112.15, 55.81.

IR: 1701 cm^{-1} (C=O), 1269 cm^{-1} (C-O).

(5-chloro-2-(isoxazol-3-yl)-1*H*-benzo[*d*]imidazol-1-yl)(4-methoxyphenyl)methanone (3d)

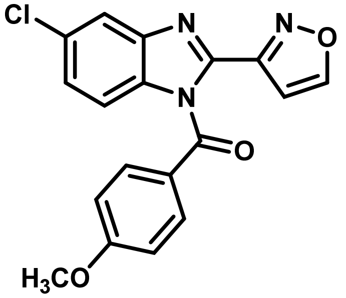

White solid. Yield = 55%. mp: 155.3-156.1°C.

^{1}H NMR (300 MHz, DMSO-d^6) δ ppm: 9.10 (d, 1H, J_1 = 1.7 Hz), 8.02 (d, 1H, J_1 = 1.9 Hz), 7.75 (d, 2H, J_1 = 8.8 Hz), 7.59 (dd, 1H, J_1 = 8.7, Hz, J_2 = 1.9 Hz), 7.37 (d, 1H, J_1 = 8.7 Hz), 7.15 (d, 1H, J_1 = 1.7 Hz), 7.05 (d, 2H, J_1 = 8.9 Hz), 3.85 (s, 3H).

^{13}C NMR (75.5 MHz, DMSO-d^6) δ ppm: 166.42, 164.71, 161.33, 153.56, 143.50, 142.95, 133.28, 133.22, 128.60, 125.78, 123.70, 119.86, 114.66, 113.86, 105.03, 55.80.

IR: 1704 cm^{-1} (C=O), 1261 cm^{-1} (C-O).

(6-chloro-2-(isoxazol-3-yl)-1*H*-benzo[*d*]imidazol-1-yl)(4-methoxyphenyl)methanone (3d')

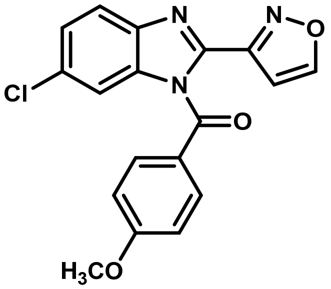

White solid. Yield = 45%. mp: 135-7 -136.7 °C.

^{1}H NMR (300 MHz, DMSO-d^{6}) δ ppm: 9.09 (d, 1H, J_1 = 1.7 Hz), 7.94 (d, 1H, J_1 = 8.6 Hz), 7.75 (d, 2H, J_1 = 8.9 Hz), 7.48 (dd, 1H, J_1 = 8.6 Hz, J_2 = 1.9 Hz), 7.44 (d, 1H, J_1 = 1.7 Hz), 7.14 (d, 1H, J_1 = 1.7 Hz), 7.06 (d, 2H, J_1 = 8.9 Hz), 3.85 (s, 3H).

^{13}C NMR (75.5 MHz, DMSO-d^{6}) δ ppm: 167.00, 165.31, 161.92, 154.21, 143.67, 141.55, 135.66, 133.86, 130.60, 125.40, 124.36, 122.43, 115.25, 112.96, 105.63, 56.43.

IR: 1704 cm^{-1} (C=O), 1261 cm^{-1} (C-O).

4) Procedure for the synthesis of (5 or 6)-chloro-1-(4-methoxybenzyl)-2-aryl-1*H*-benzo[*d*]imidazole 4(a-d) and 4(a'-d')

To a solution of the corresponding 6-chloro-2-aryl-1*H*-benzo[*d*]imidazoles **2(a-d)** (0.40 mmol) and sodium hydride (NaH) (1.4 mmol) in dry THF (20 mL) under anaerobic conditions (N_2) and magnetic stirring was added dropwise a solution of 1-bromomethyl-4-methoxybenzene (**7**) (0.805 mmol) in 5 mL of anhydrous THF. After addition, reaction was stirred under reflux for 3 h. Subsequently, the remaining NaH was removed by filtration and the filtrate was poured over 100 mL of water and extracted with ethyl acetate. The organic portions were dried with anhydrous Na_2SO_4, filtered and concentrated under reduced pressure in a rotary evaporator. The residue was purified by thin layer chromatography with 30% ethyl acetate and 70% hexane as eluent to yield target regioisomers **4(a-d)** and **4(a'-d')**.

5-chloro-1-(4-methoxybenzyl)-2-(3-methoxyphenyl)-1*H*-benzo[*d*]imidazolemethanone (4a)

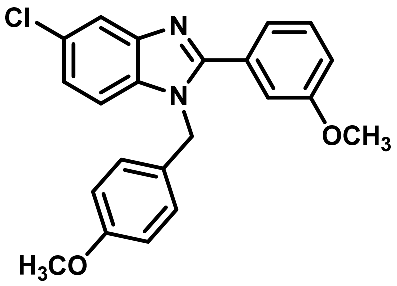

White solid. Yield = 54%. mp: 123.6 – 124.8 °C.

^{1}H NMR (300 MHz, $CDCl_3$) δ ppm: 7.85 (d, 1H, J_1 = 1.7 Hz), 7.39 (t, 1H, J_1 = 8.1 Hz), 7.23 (m, 2H), 7.21 (d, 1H, J_1 = 1.8 Hz), 7.14 (d, 1H, J_1 = 8.6 Hz), 7.07 (d, 1H, J_1 = 1.7 Hz), 7.01 (d, 2H, J_1 = 8.9 Hz), 6.88 (d, 2H, J_1 = 8.6 Hz), 5.41 (s, 2H), 3.80 (s, 3H), 3.78 (s, 3H).

^{13}C NMR (75.5 MHz, $CDCl_3$) δ ppm: 159.94, 159.38, 155.30, 144.05, 134.78, 131.02, 130.01, 128.36, 128.05, 127.33, 123.57, 121.54, 119.86, 166.81, 114.62, 114.28, 111.48, 55.46, 55.44, 48.21.

IR: 1247 cm^{-1} (C-O)

6-chloro-1-(4-methoxybenzyl)-2-(3-methoxyphenyl)-1*H*-benzo[*d*]imidazolemethanone (4a')

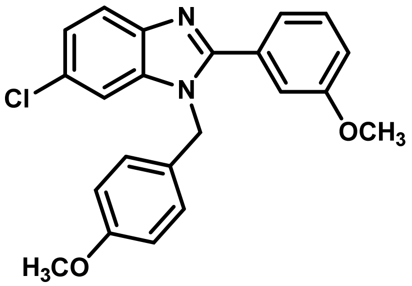

White solid. Yield = 46%. mp: 114.3 – 115.7 °C.

^{1}H NMR (300 MHz, $CDCl_3$) δ ppm: 7.78 (d, 1H, J_1 = 8.6 Hz), 7.38 (t, 1H, J_1 = 8.1 Hz), 7.29 (dd, 1H, J_1 = 8.6 Hz, J_2 = 1.9 Hz), 7.25 (m, 3H), 7.05 (dd, 1H, J_1 = 2.4 Hz, J_2 = 0.9 Hz), 7.03 (d, 2H, J_1 = 8.5 Hz), 6.88 (d, 2H, J_1 = 8.5 Hz), 5.39 (s, 2H), 3.81 (s, 3H), 3.77 (s, 3H).

^{13}C NMR (75.5 MHz, $CDCl_3$) δ ppm: 159.81, 159.28, 154.81, 141.67, 136.69, 130.81, 129.88, 128.75, 127.80, 127.17, 123.41, 121.38, 120.82, 116.72, 114.57, 114.09, 110.59, 55.33, 48.05.

IR: 1247 cm^{-1} (C-O).

5-chloro-1-(4-methoxybenzyl)-2-(pyridin-3-yl)-1*H*-benzo[*d*]imidazole (4b)

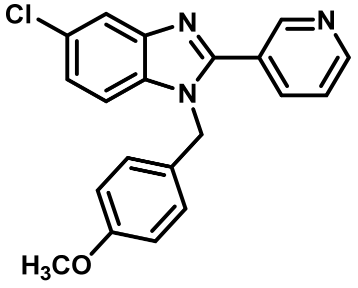

White solid. Yield = 48%. mp: 121.0-121.9°C.

^{1}H NMR (300 MHz, $CDCl_3$) δ ppm: 8.92 (s, 1H), 8.73 (d, 1H, J_1 = 4.8 Hz), 8.01 (dd, 1H, J_1 = 7.9 Hz, J_2 = 1.7 Hz), 7.84 (s, 1H), 7.41 (dd, 1H, J_1 = 7.9 Hz, J_2 = 4.9 Hz), 7.24 (m, 1H), 7.18 (dd, 1H, J_1 = 8.6 Hz, J_2 = 0.9 Hz), 6.96 (d, 2H, J_1 = 8.3 Hz), 6.85 (d, 2H, J_1 = 8.3 Hz), 5.39 (s, 2H), 3.78 (s, 3H).

^{13}C NMR (75.5 MHz, $CDCl_3$) δ ppm: 195.55, 152.33, 151.17, 149.78, 144.09, 136.81, 134.84, 128.70, 127.47, 127.20, 126.26, 124.12, 123.69, 120.05, 114.76, 111.56, 55.44, 48.20.

IR: 1244 cm^{-1} (C-O).

6-chloro-1-(4-methoxybenzyl)-2-(pyridin-3-yl)-1*H*-benzo[*d*]imidazole (4b')

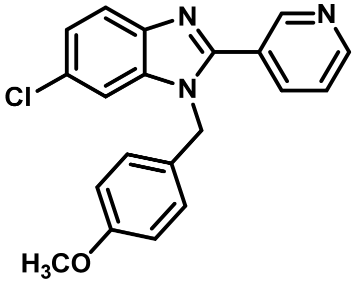

White solid. Yield = 52%. mp: 127.9 – 129.1°C.

^{1}H NMR (300 MHz, $CDCl_3$) δ ppm: 8.93 (d, 1H, J_1 = 1.4 Hz), 8.73 (dd, 1H, J_1 = 4.7 Hz, J_2 = 1.4 Hz), 8.00 (dt, 1H, J_1 = 7.9 Hz, J_2 = 1.9 Hz), 7.76 (d, 1H, J_1 = 8.5 Hz), 7.41 (dd, 1H, J_1 = 7.9 Hz, J_2 = 4.9 Hz), 7.30 (dd, 1H, J_1 =8.5 Hz, J_2 = 1.9 Hz), 7.27 (d, 1H, J_1 = 1.9 Hz), 6.98 (d, 2H, J_1 = 8.7Hz), 6.87 (d, 2H, J_1 = 8.7 Hz), 5.37 (s, 2H), 3.79 (s, 3H).

^{13}C NMR (75.5 MHz, $CDCl_3$) δ ppm: 159.57, 151.96, 151.12, 149.78, 141.92, 136.74, 129.43, 127.36, 127.17, 126.25, 123.88, 123.69, 121.17, 114.84, 110.76, 55.45, 48.15.

IR: 1244 cm^{-1} (C-O).

5-chloro-2-(furan-2-yl)-1-(4-methoxybenzyl)-1*H*-benzo[*d*]imidazole (4c)

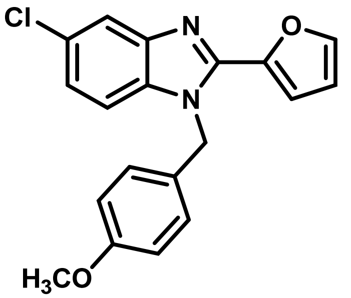

White solid. Yield = 56%. mp: 147.0 – 148.0 °C

^{1}H NMR (300 MHz, $CDCl_3$) δ ppm: 7.77 (d, 1H, J_1 = 1.2 Hz), 7.59 (d, 1H, J_1 = 1.6 Hz), 7.19 (d, 1H, J_1 = 0.9 Hz), 7.11 (d, 1H, J_1 = 3.5 Hz), 7.05 (d, 2H, J_1 = 8.6 Hz), 6.82 (d, 2H, J_1 = 8.6 Hz), 6.57 (dd, 1H, J_1 = 3.4 Hz, J = 1.7 Hz), 5.61 (s, 2H), 3.75 (s, 3H).

^{13}C NMR (75.5 MHz, $CDCl_3$) δ ppm: 159.38, 145.60, 145.03, 144.45, 144.09, 134.48, 128.61, 128.04, 127.72, 123.74, 119.67, 114.49, 113.45, 112.23, 110.99, 55.40, 48.17.

IR: 1247 cm^{-1} (C-O).

6-chloro-2-(furan-2-yl)-1-(4-methoxybenzyl)-1*H*-benzo[*d*]imidazole (4c')

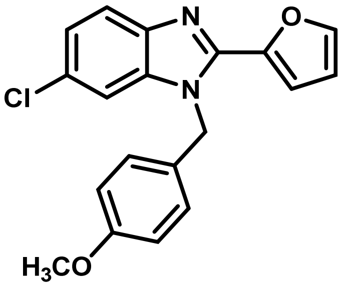

White solid. Yield = 44%. mp: 113.9 - 115.1°C.

^{1}H NMR (300 MHz, $CDCl_3$) δ ppm: 7.70 (d, 1H, J_1 = 8.5 Hz), 7.59 (dd, 1H, J_1 = 1.6 Hz, J_2 = 0.6 Hz), 7.27 (t, 1H, J_1 = 2.0 Hz), 7.24 (dd, 1H, J_1 = 8.5 Hz, J_2 = 1.9 Hz), 7.9 (dd, 1H, J_1 = 3.5 Hz, J_2 = 0.9 Hz), 7.06 (d, 2H, J_1 = 8.7 Hz), 6.84 (d, 2H, J_1 = 8.7 Hz), 6.56 (dd, 1H, J_1 = 3.5 Hz, J_2 = 1.8 Hz), 5.58 (s, 2H), 3.76 (s, 3H).

^{13}C NMR (75.5 MHz, $CDCl_3$) δ ppm: 159.39, 145.21, 145.00, 144.39, 141.87, 136.46, 129.00, 127.91, 127.70, 123.75, 120.77, 114.53, 113.29, 112.21, 110.26, 55.39, 48.12.

IR: 1249 cm^{-1} (C-O).

3-(5-chloro-1-(4-methoxybenzyl)-1*H*-benzo[*d*]imidazol-2-yl)isoxazole (4d)

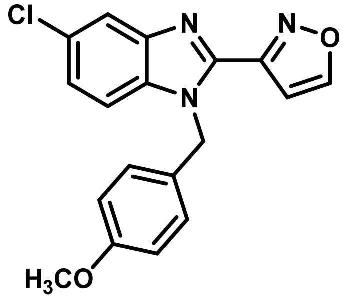

White solid. Yield = 42%. mp: 151.2-152.0°C

^{1}H NMR (300 MHz, $CDCl_3$) δ ppm: 9.22 (d, 1H, J_1 = 1.7 Hz), 7.86 (d, 1H, J_1 =1.7 Hz), 7.70 (d, 1H, J_1 = 8.7 Hz), 7.37 (dd, 1H, J_1 = 8.7 Hz, J_2 = 2.0 Hz), 7.26 (d, 1H, J_1 = 1.7 Hz), 7.12 (d, 2H, J_1 = 8.6 Hz), 6.84 (d, 2H, J_1 =8.6 Hz), 5.90 (s, 2H), 3.67 (s, 3H).

^{13}C NMR (75.5 MHz, $CDCl_3$) δ ppm: 160.98, 158.69, 154.96, 143.56, 143.32, 134.71, 128.39, 128.18, 127.41, 124.34, 119.28, 114.08, 113.13, 105.83, 55.02, 47.57.

IR: 1244 cm^{-1} (C-O).

3-(6-chloro-1-(4-methoxybenzyl)-1*H*-benzo[*d*]imidazol-2-yl)isoxazole (4d')

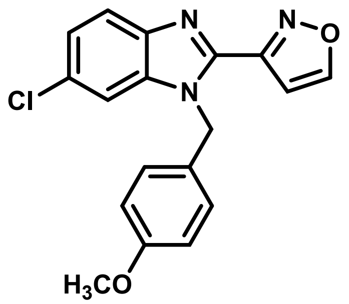

White solid. Yield = 58%. mp: 179.2-180.4 °C

^{1}H NMR (300 MHz, $CDCl_3$) δ ppm: 9.21 (d, 1H, J_1 = 1.6 Hz), 7.83 (d, 1H, J_1 = 1.7 Hz), 7.79 (d, 1H, J_1 = 8.6 Hz), 7.33 (dd, 1H, J_1 = 8.5 Hz, J_2 = 1.9 Hz), 7.25 (d, 1H, J_1 = 1.6 Hz), 7.12 (d, 2H, J_1 = 8.6 Hz), 6.85 (d, 2H, J_1 = 8.6 Hz), 5.91 (s, 1H), 3.68 (s, 3H).

^{13}C NMR (75.5 MHz, $CDCl_3$) δ ppm: 160.93, 158.69, 154.97, 143.27, 141.30, 136.61, 128.67, 128.40, 128.16, 123.40, 121.32, 114.10, 111.53, 105.80, 55.02, 47.43.

IR: 1244 cm^{-1} (C-O).

NMR spectra of compounds

5(6)-chloro-2-(3-methoxyphenyl)-1*H*-benzo[*d*]imidazole (**2a**)

^{1}H NMR (300 MHz, DMSO-d^6)

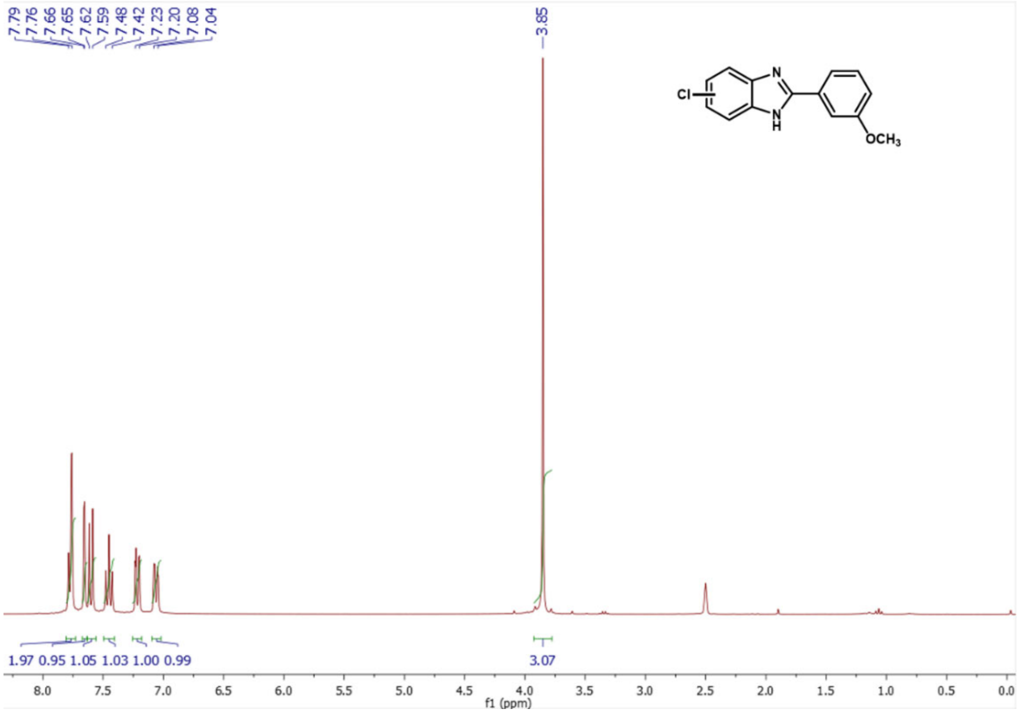

^{13}C NMR (75.5 MHz, DMSO-d^6)

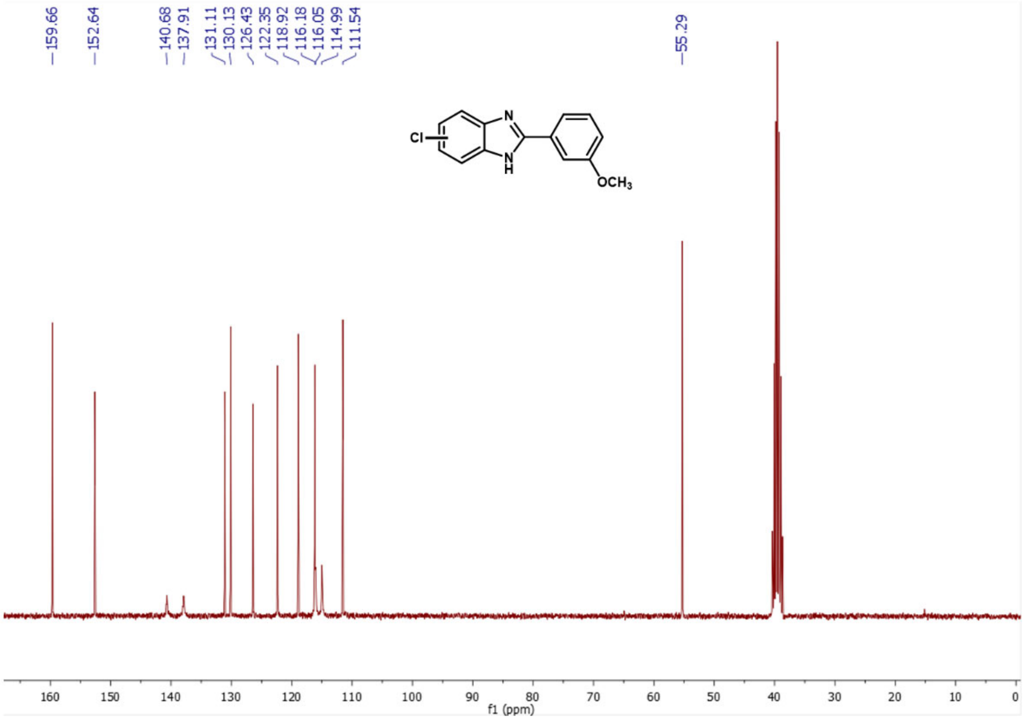

5(6)-chloro-2-(pyridin-3-yl)-1*H*-benzo[*d*]imidazole (**2b**)

^{1}H NMR (300 MHz, DMSO-d^{6})

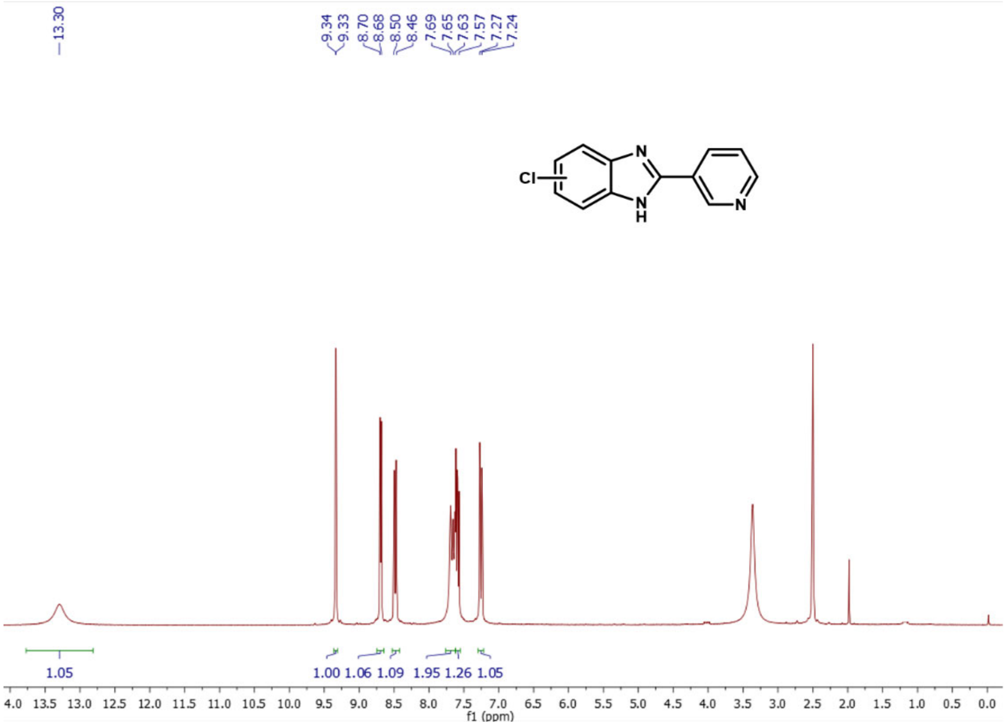

^{13}C NMR (75.5 MHz, DMSO-d^{6})

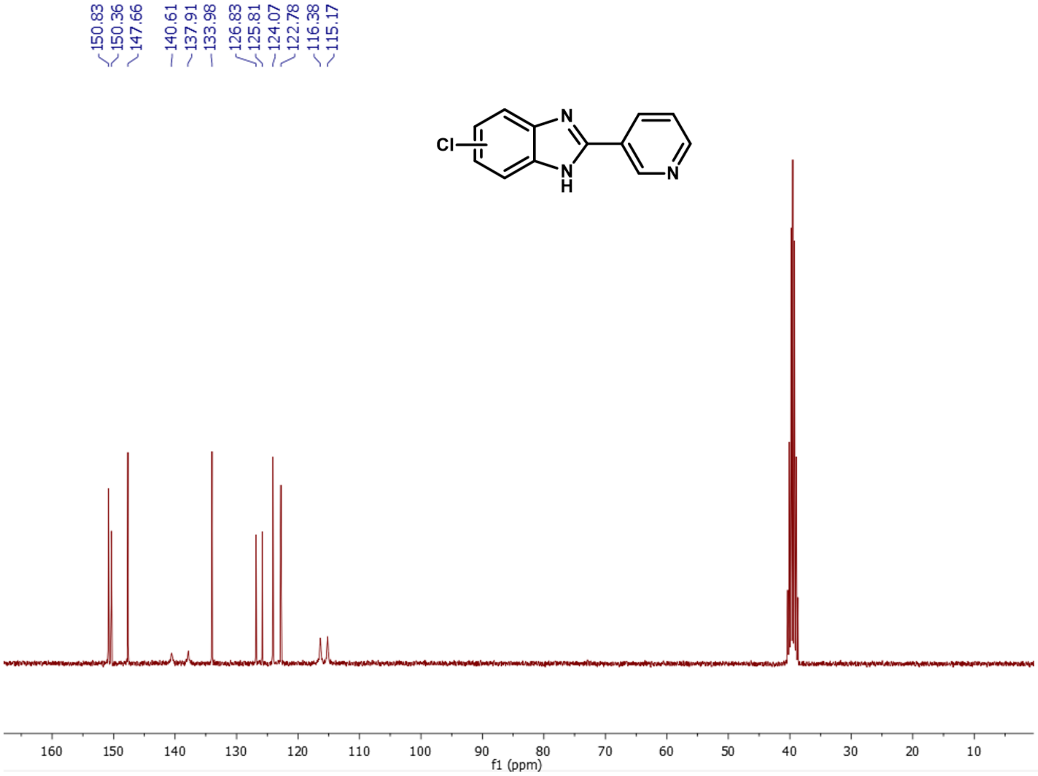

5(6)-chloro-2-(furan-2-yl)-1*H*-benzo[*d*]imidazole (**2c**)

^{1}H NMR (300 MHz, DMSO-d^{6})

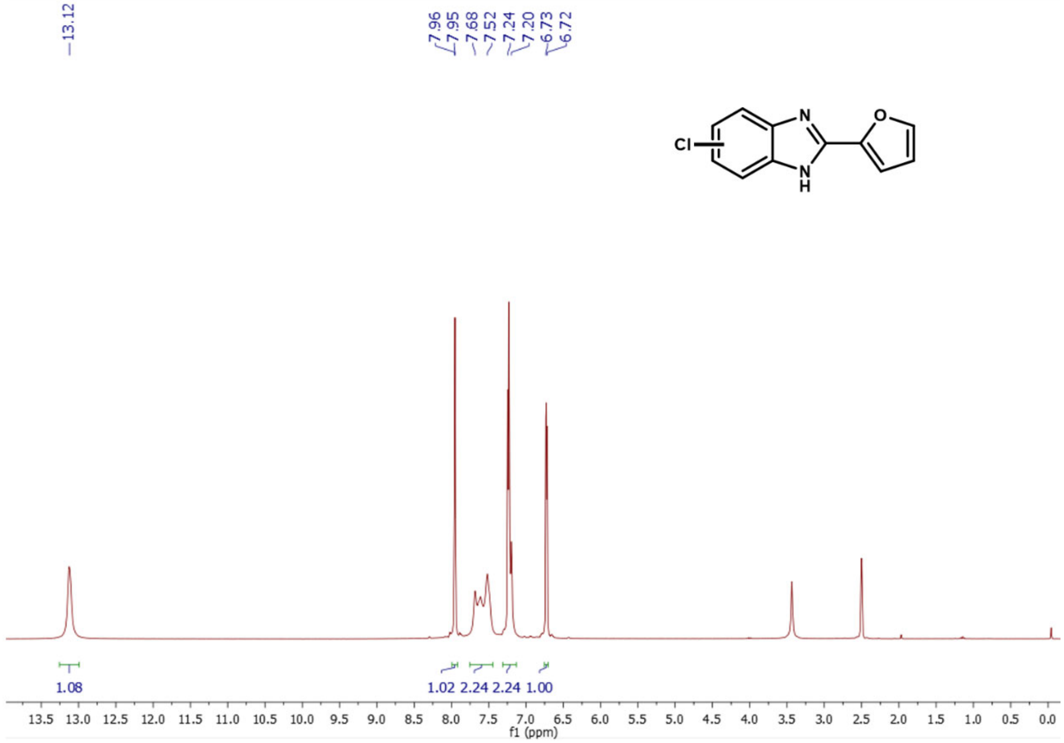

^{13}C NMR (75.5 MHz, DMSO-d^{6})

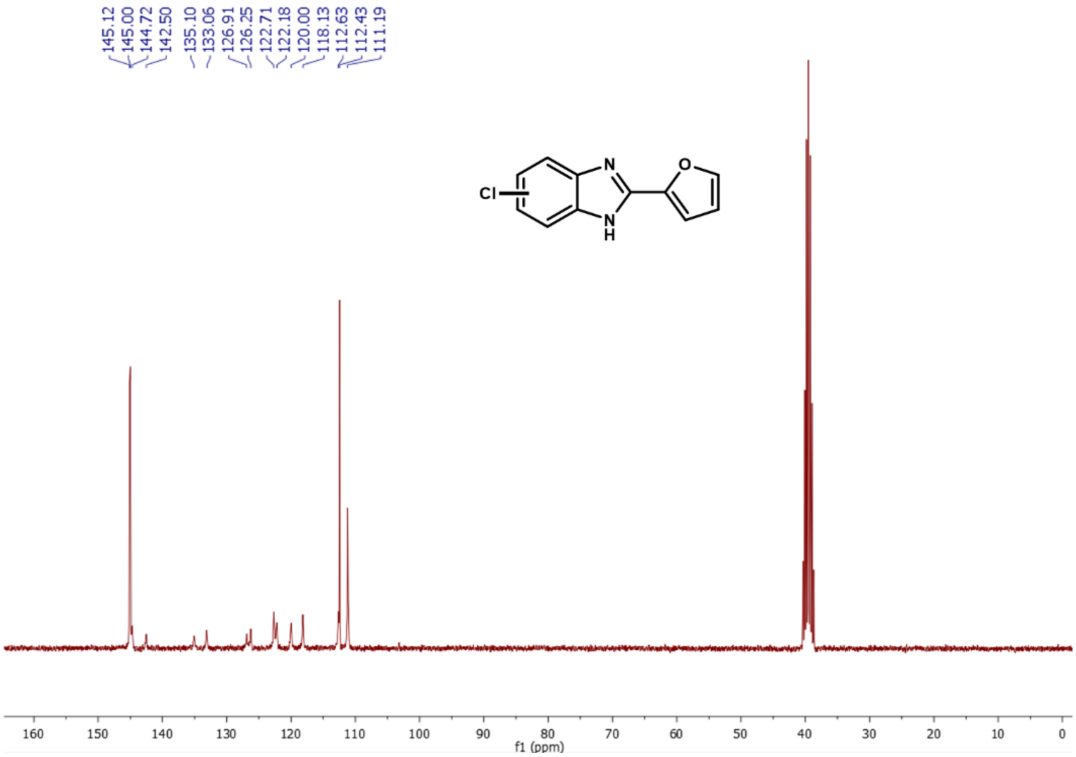

3-(5(6)-chloro-1*H*-benzo[*d*]imidazol-2-yl)isoxazole (**2d**):

^{1}H NMR (300 MHz, DMSO-d^{6})

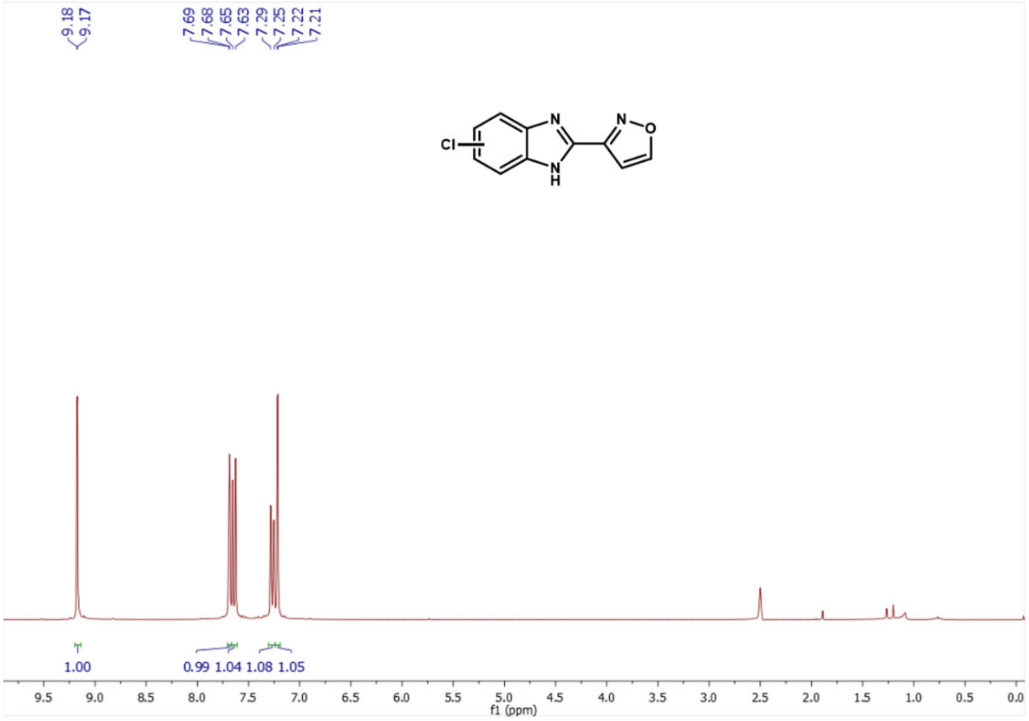

^{13}C NMR (75.5 MHz, DMSO-d^{6})

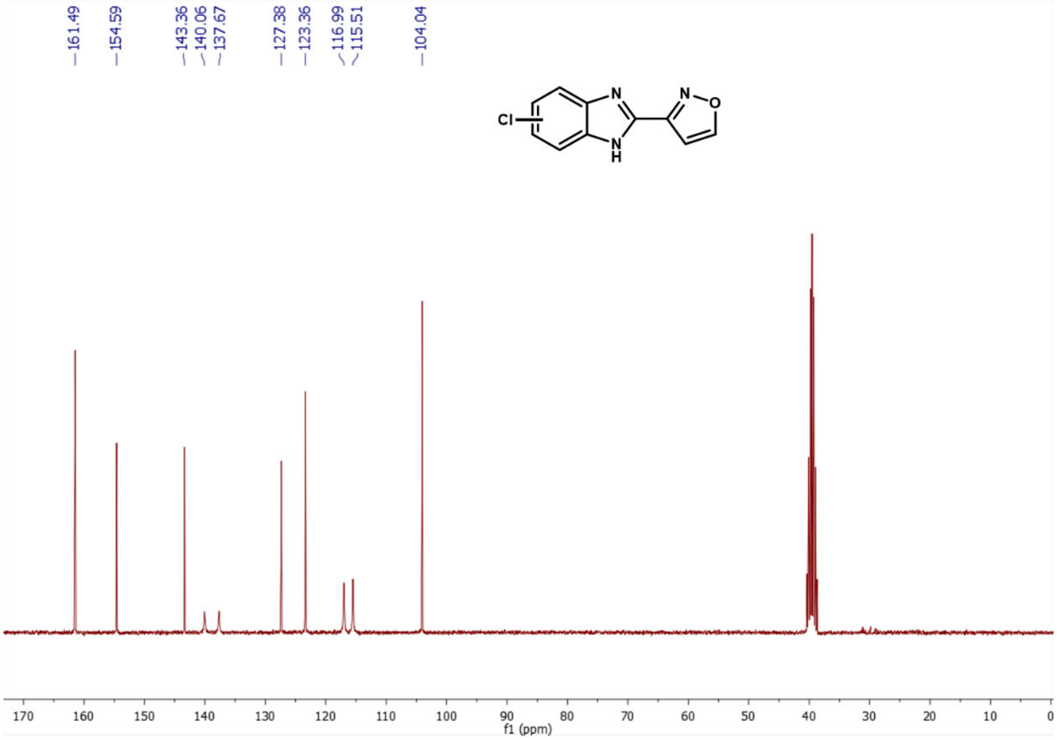

(5-chloro-2-(3-methoxyphenyl)-1*H*-benzo[*d*]imidazol-1-yl)(4-methoxyphenyl)methanone (**3a**)

^{1}H NMR (300 MHz, $CDCl_3$)

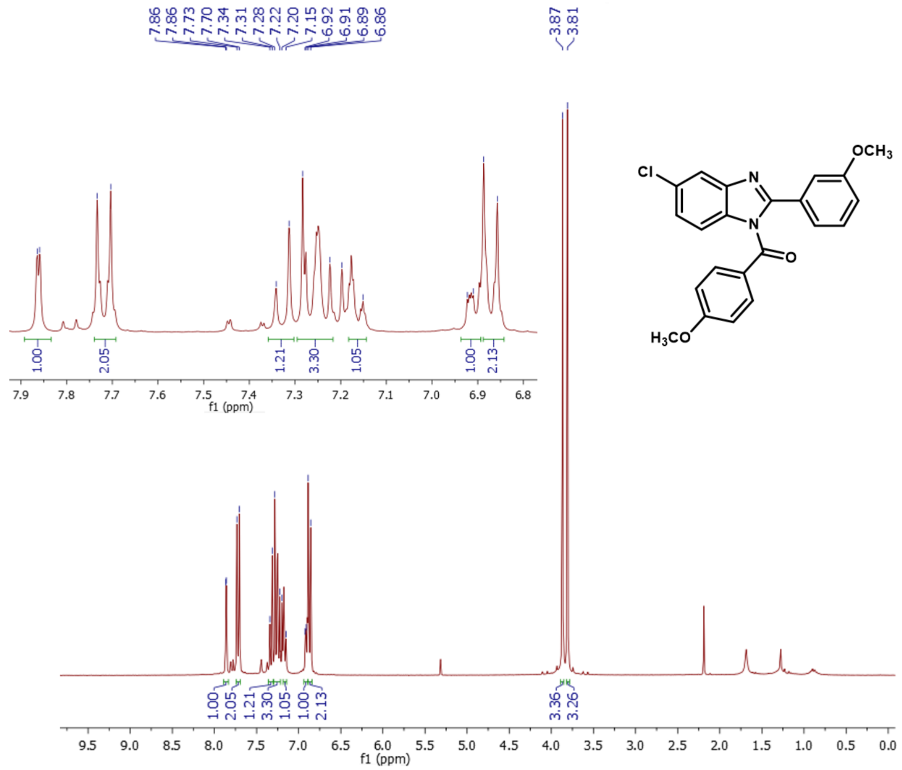

^{13}C NMR (75.5 MHz, $CDCl_3$)

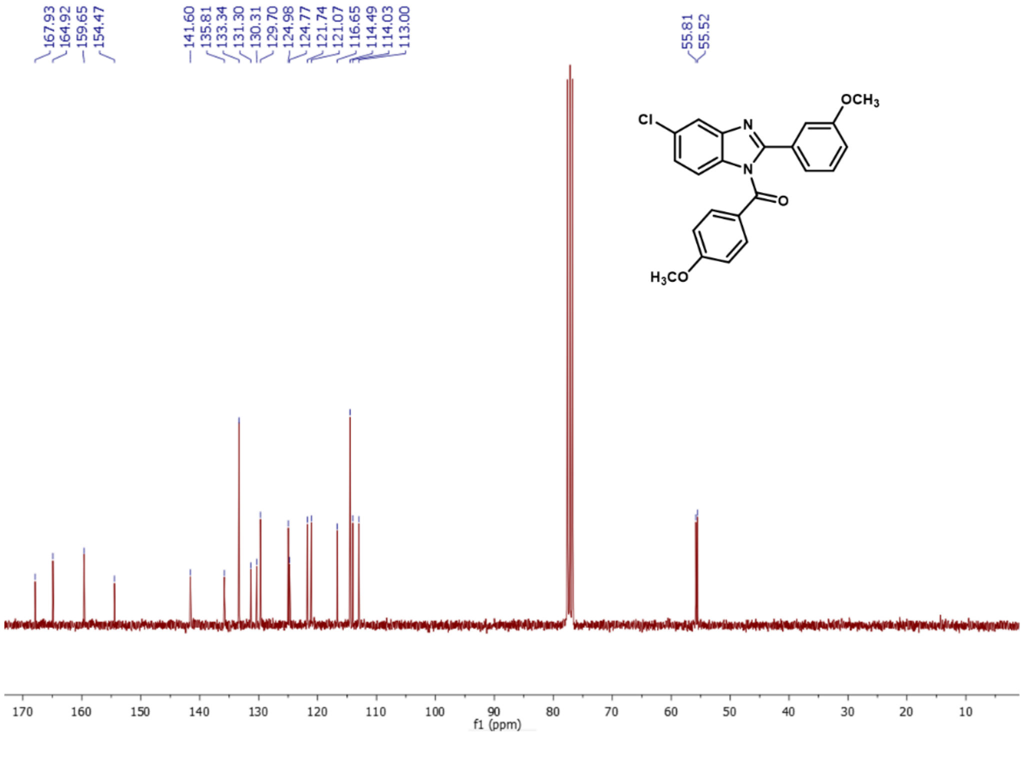

DEPT-135 – NMR

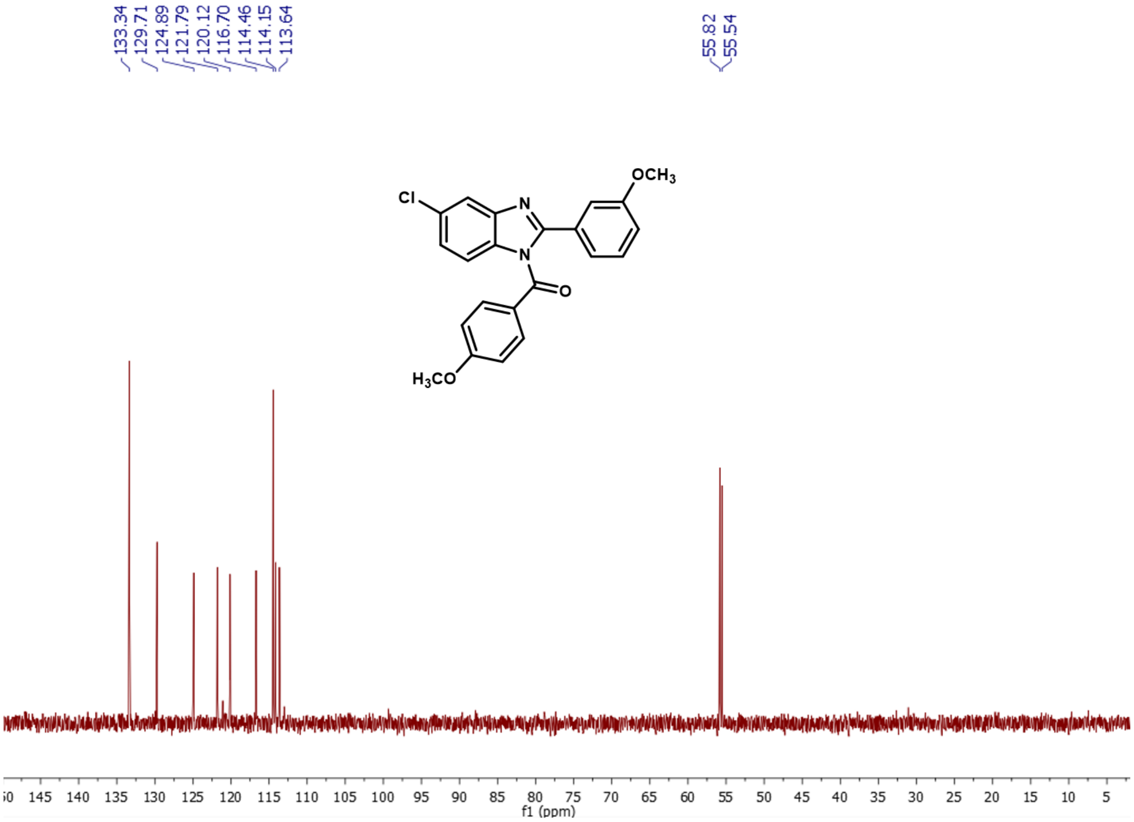

^1H–^1H – COSY NMR

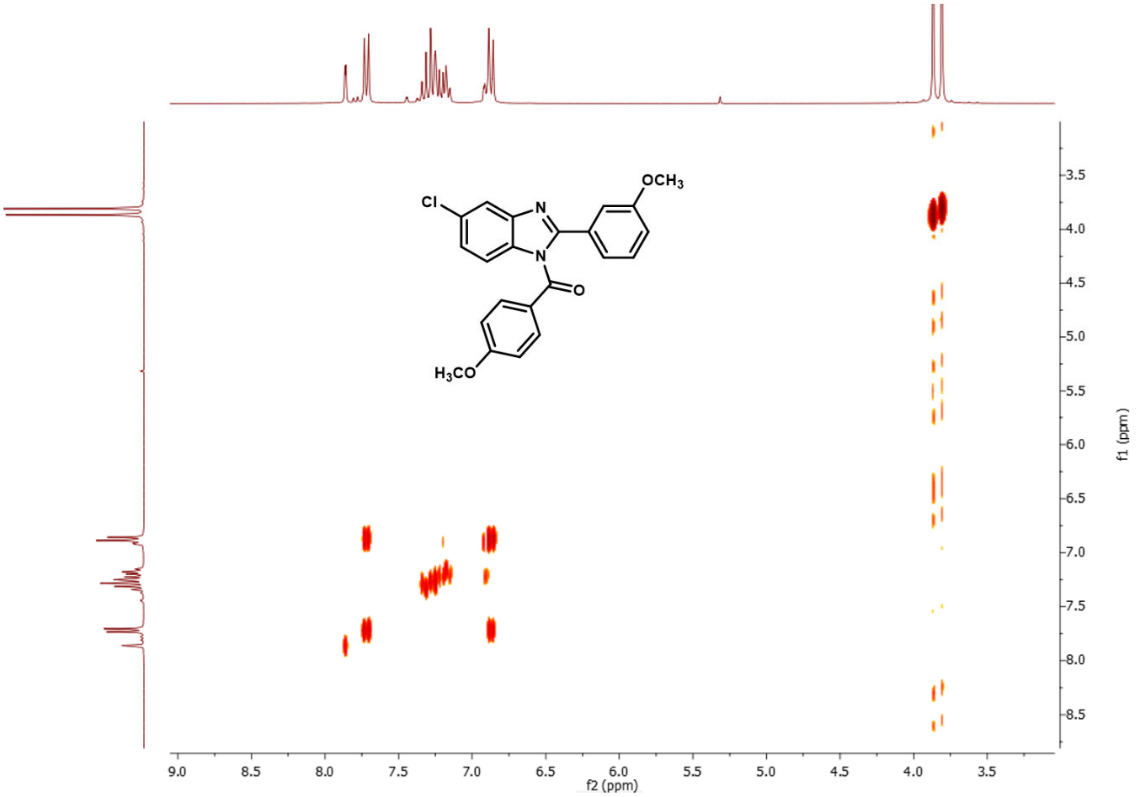

$^{1}H–^{13}C$ – HSQC NMR

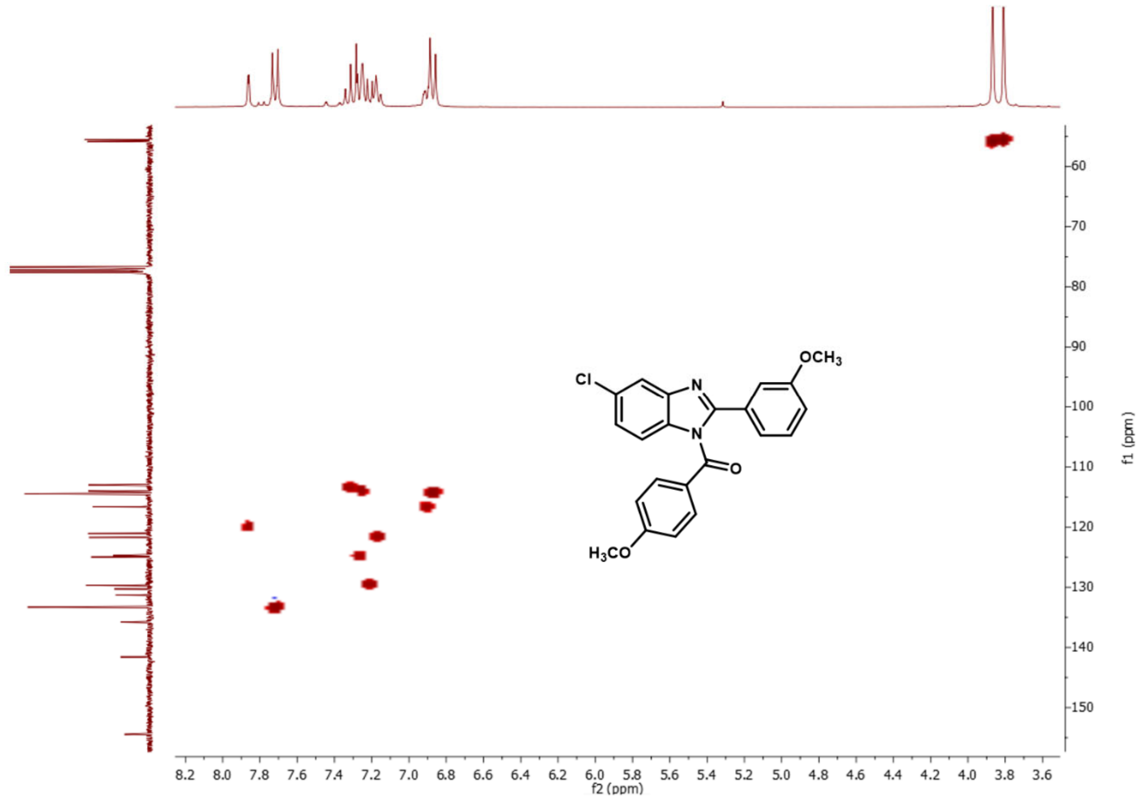

$^{1}H–^{13}C$ – HMBC NMR

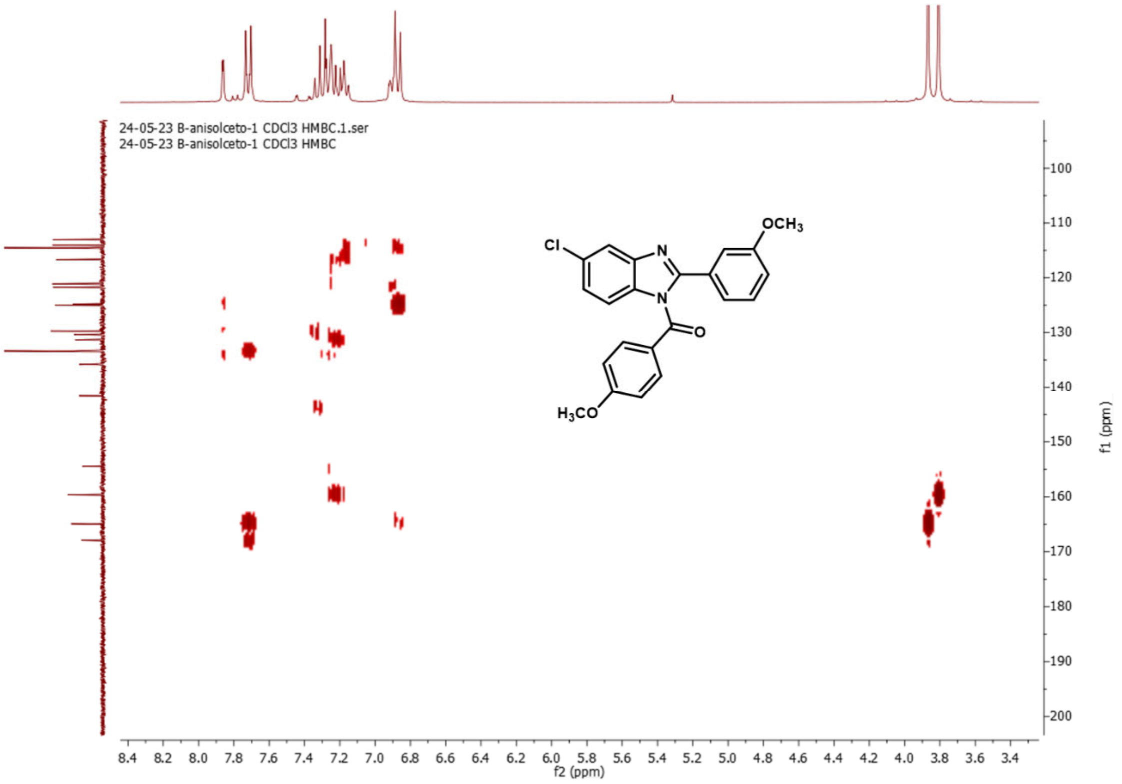

(5-chloro-2-(pyridin-3-yl)-1*H*-benzo[*d*]imidazol-1-yl)(4-methoxyphenyl)methanone (**3b**)

^{1}H NMR (300 MHz, $CDCl_3$)

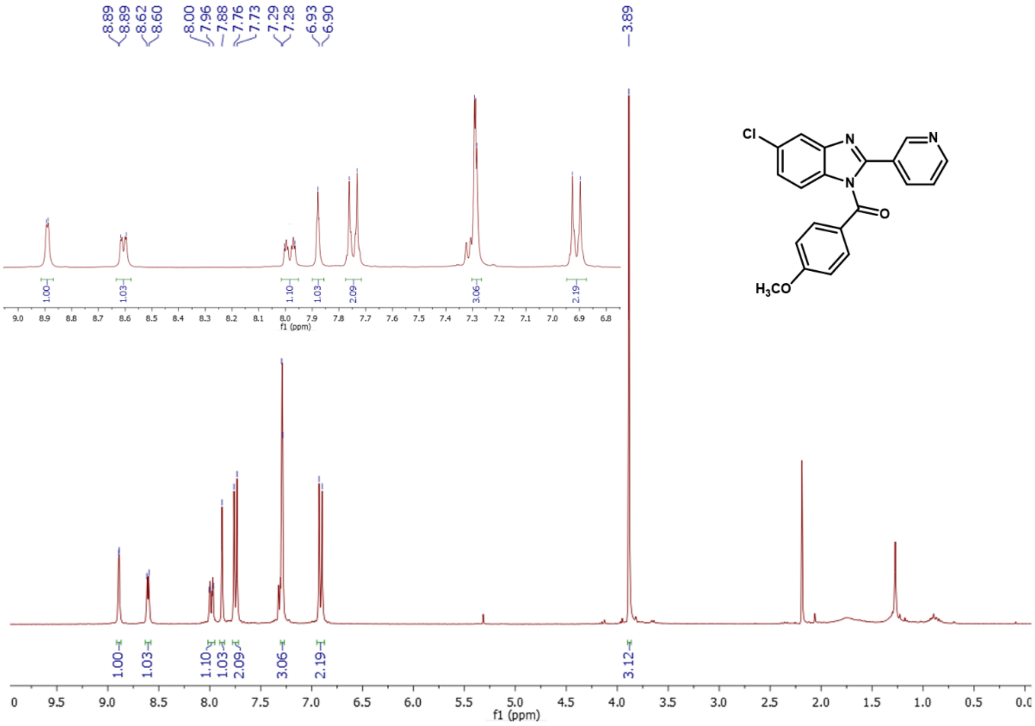

^{13}C NMR (75.5 MHz, $CDCl_3$)

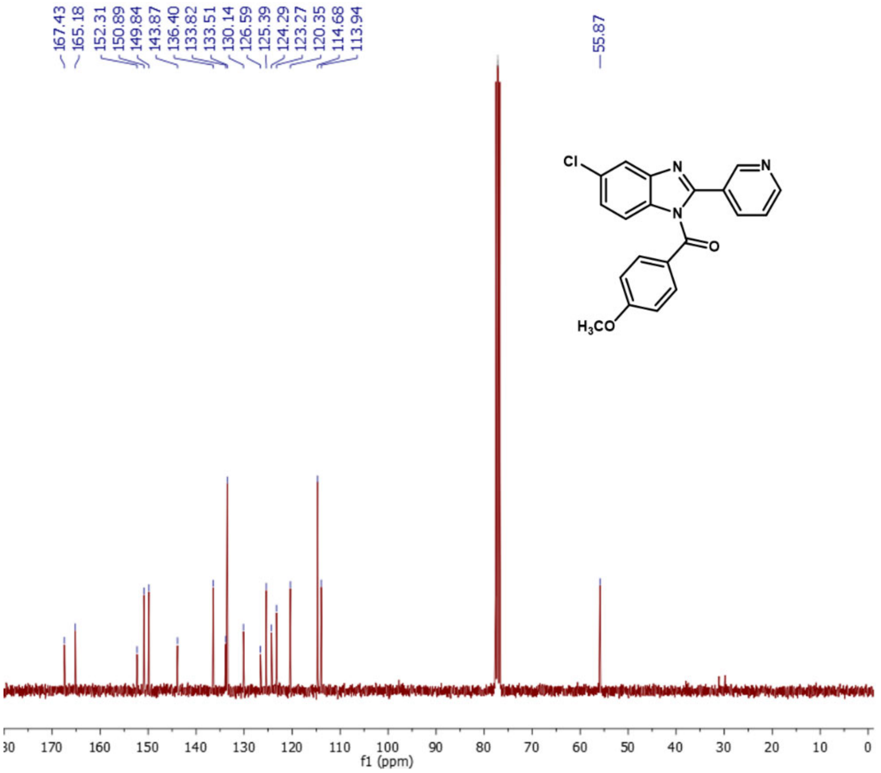

DEPT-135 – NMR

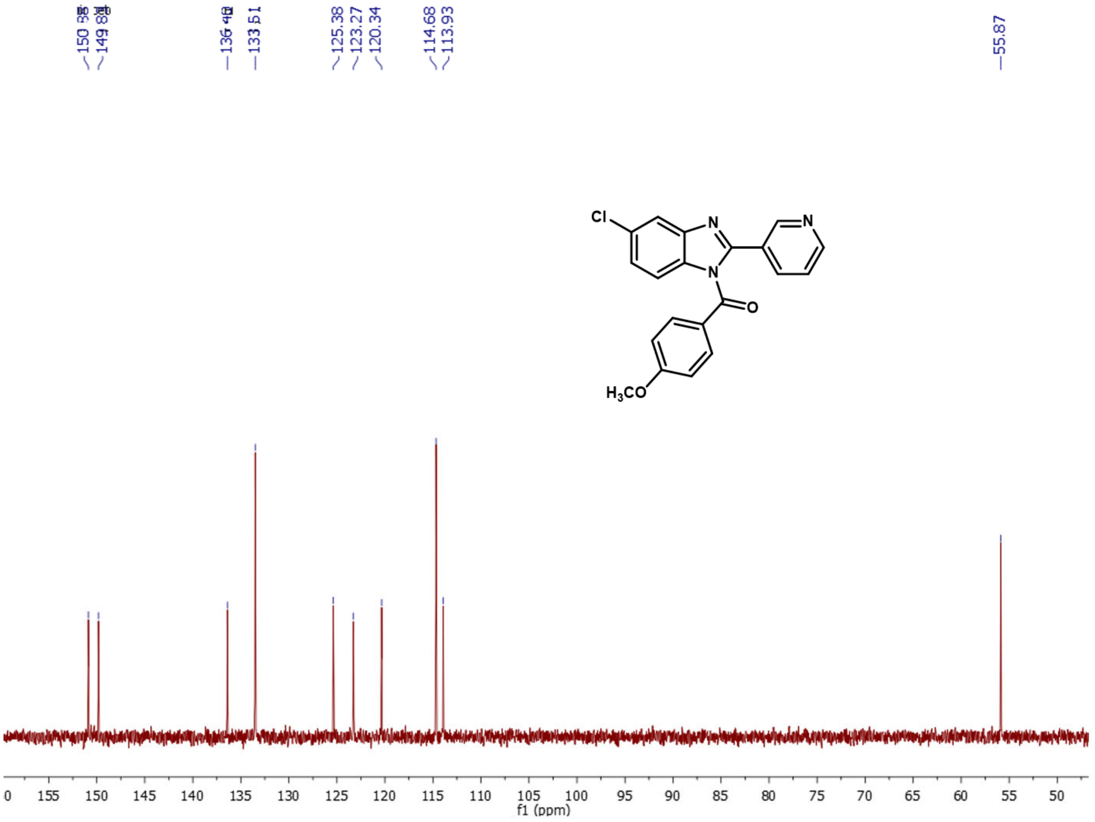

^{1}H–^{1}H – COSY NMR

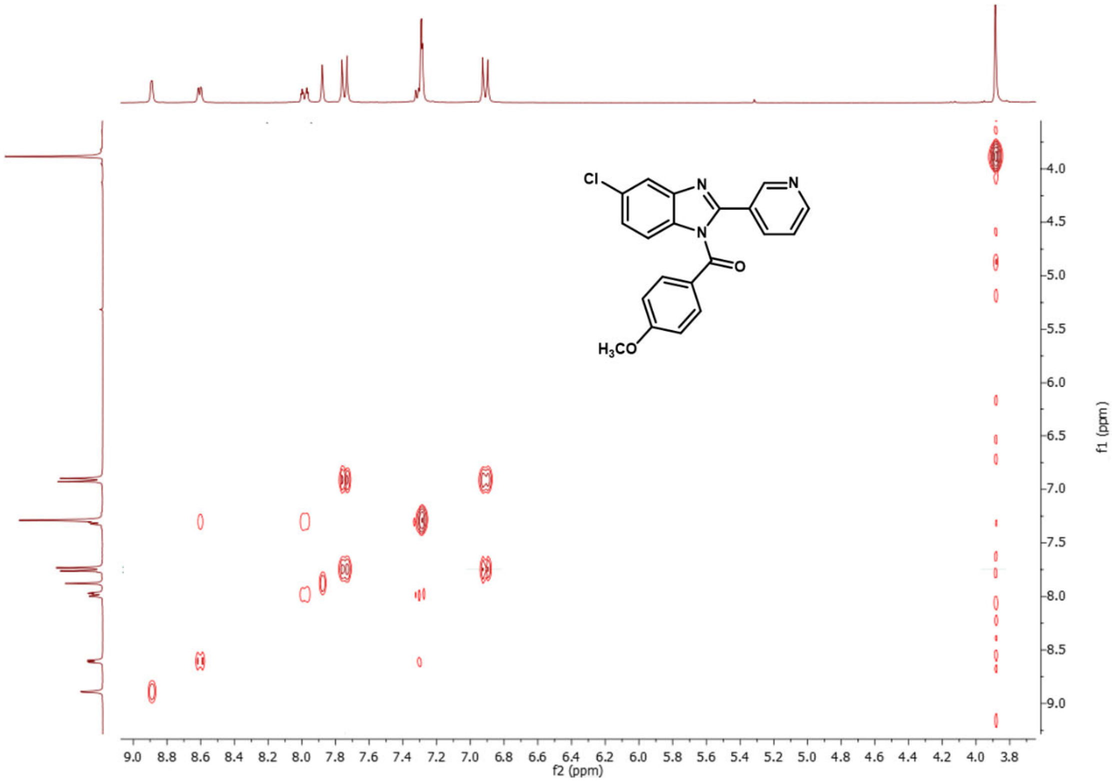

^{1}H–^{13}C – HSQC NMR

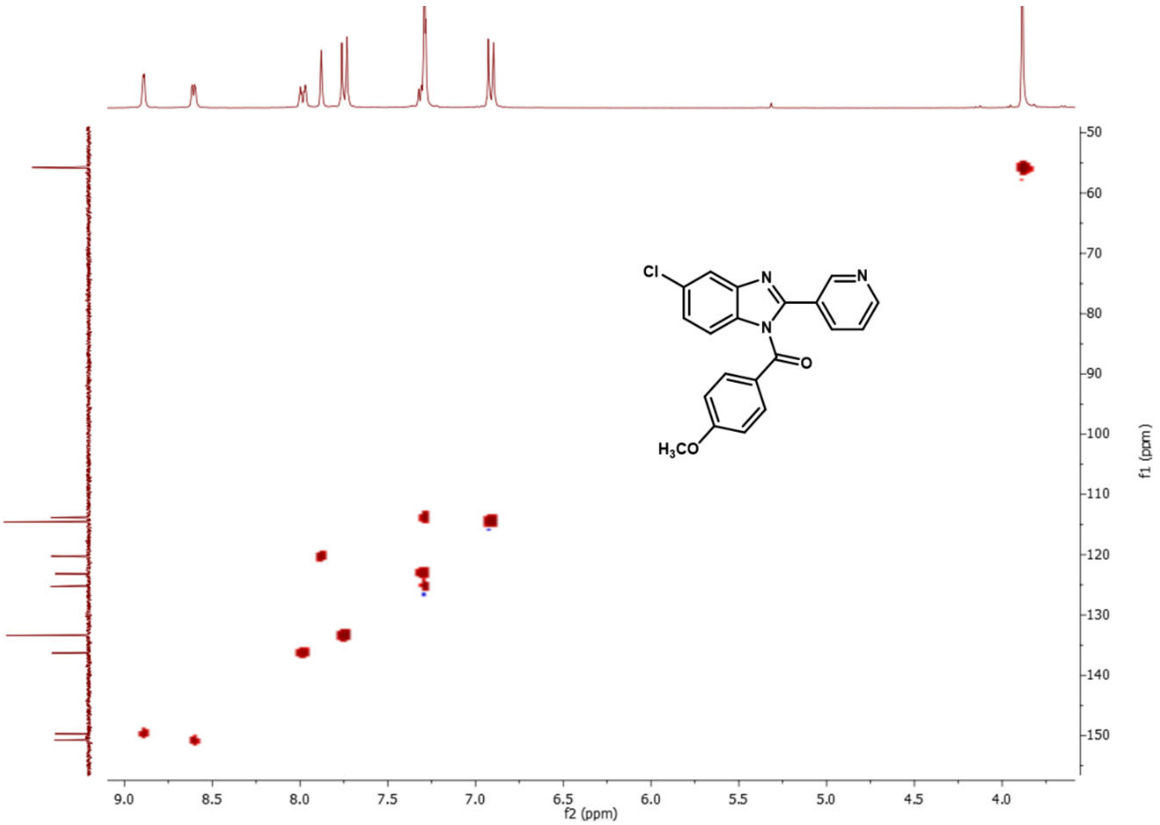

^{1}H–^{13}C – HMBC NMR

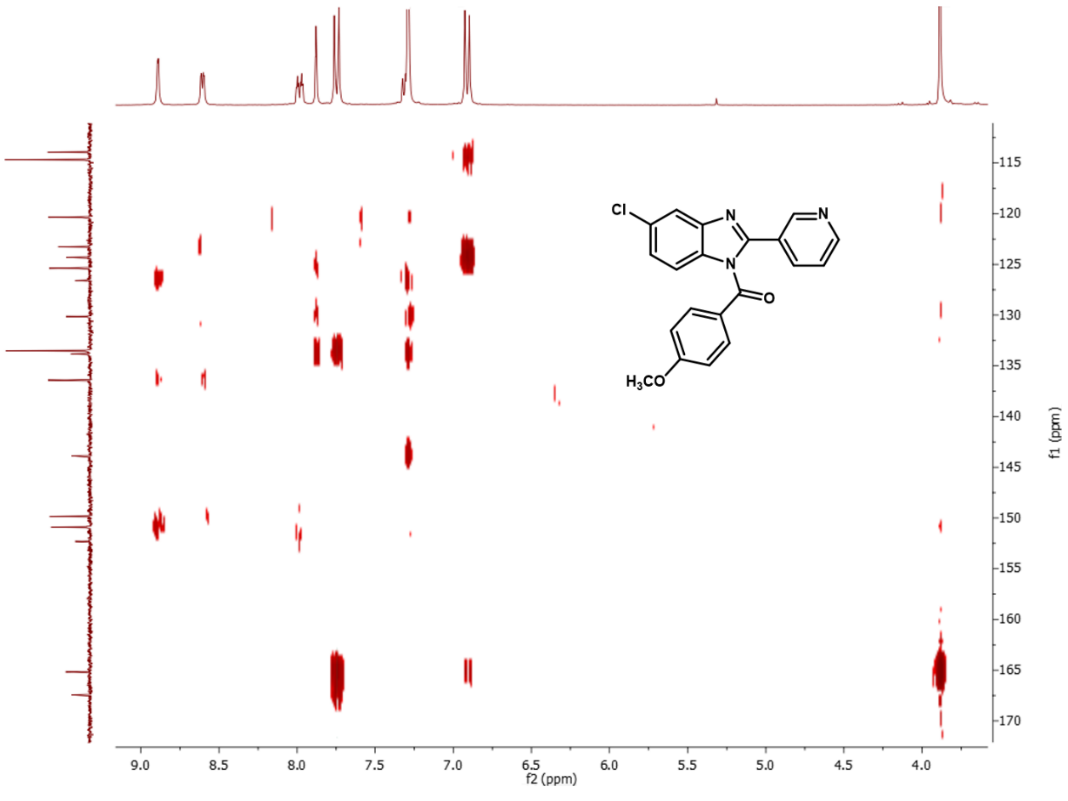

(6-chloro-2-(pyridin-3-yl)-1*H*-benzo[*d*]imidazol-1-yl)(4-methoxyphenyl)methanone (**3b'**)

^{1}H NMR (300 MHz, $CDCl_3$)

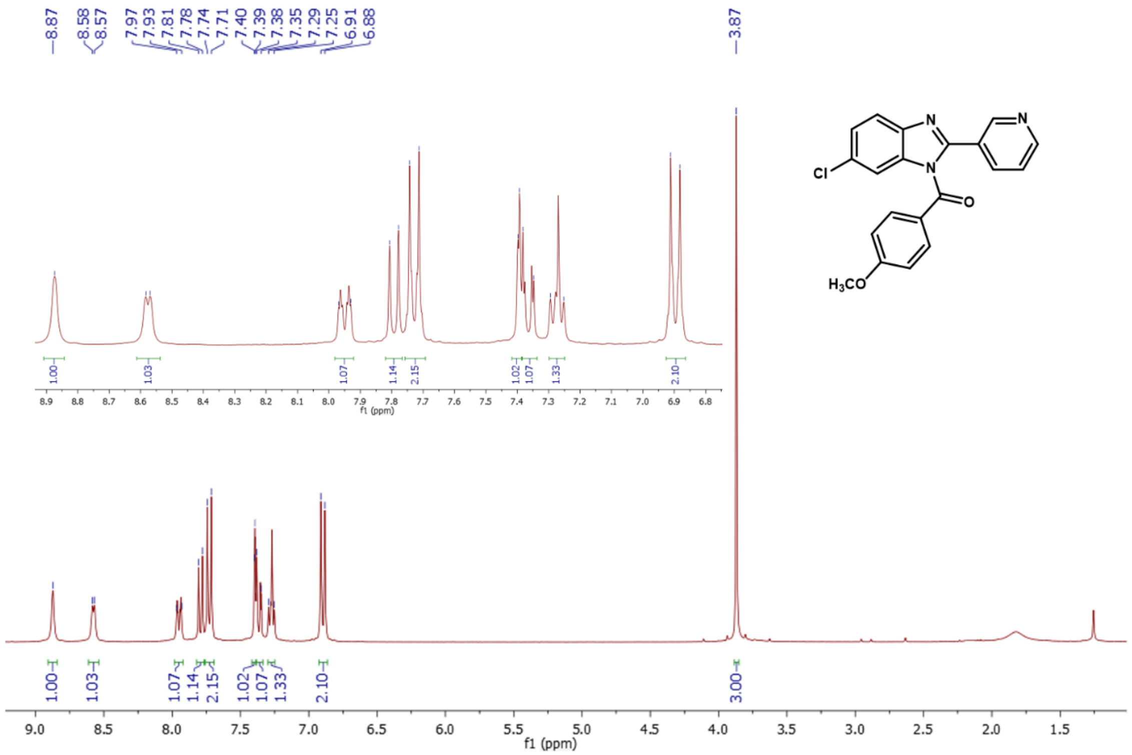

^{13}C NMR (75.5 MHz, $CDCl_3$)

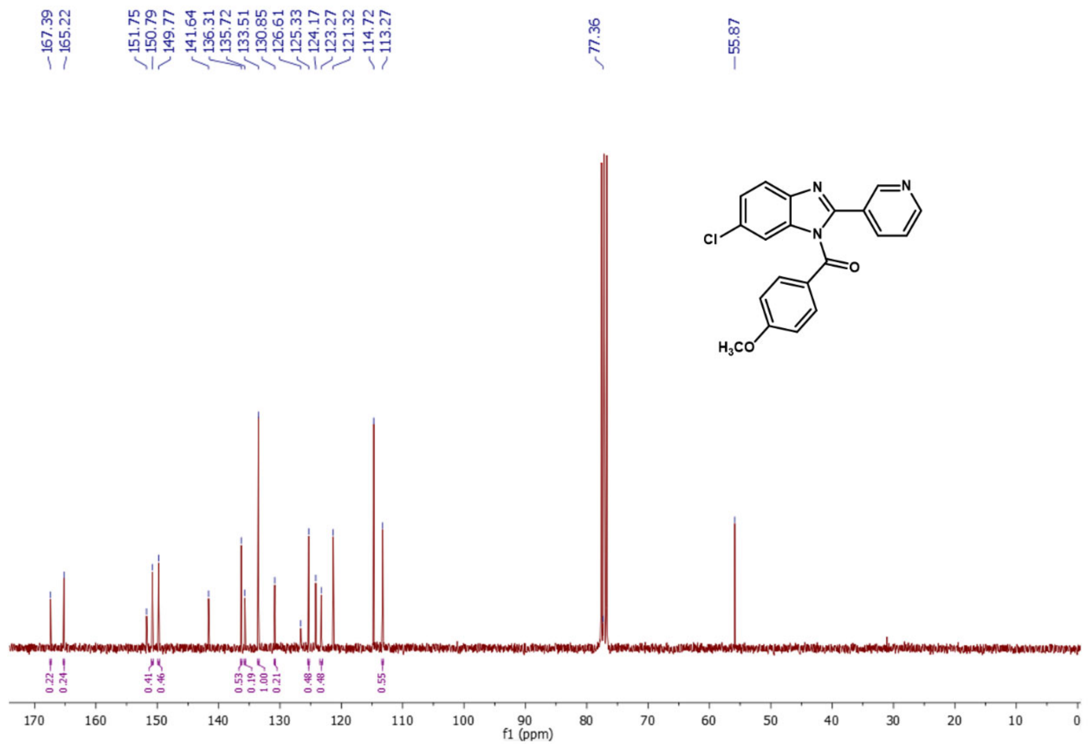

DEPT-135 – NMR

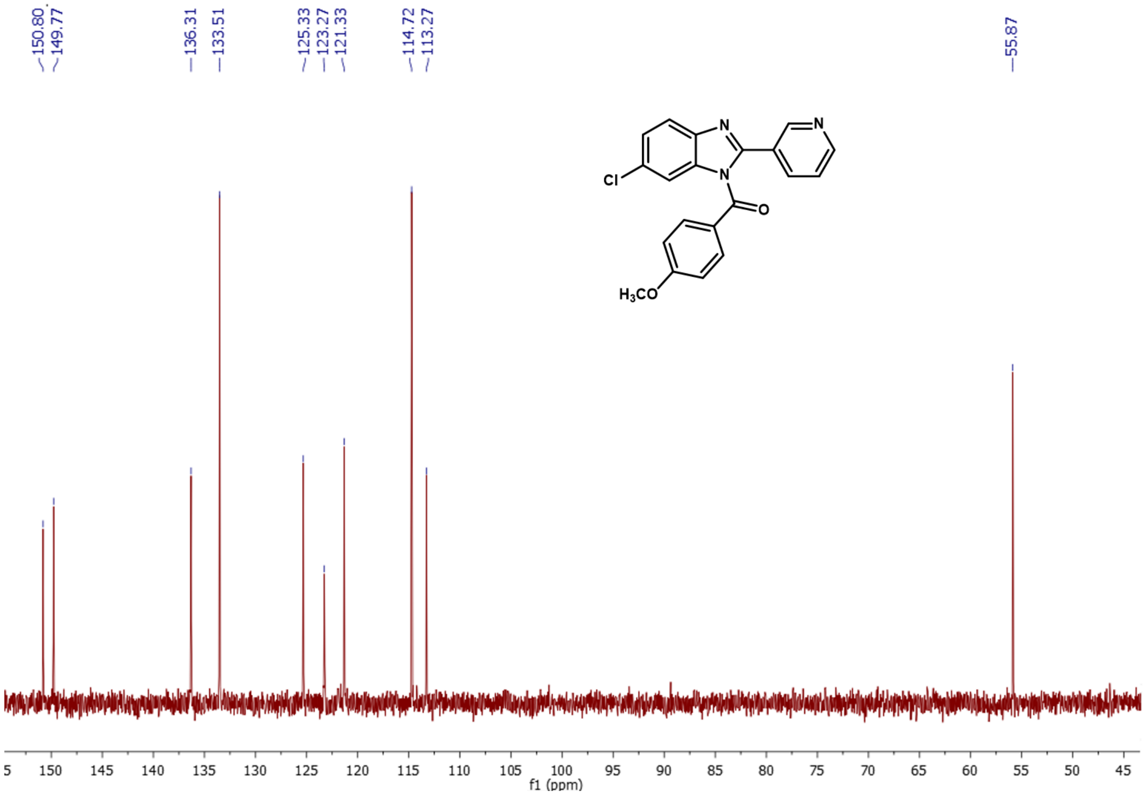

^1H–^1H – COSY NMR

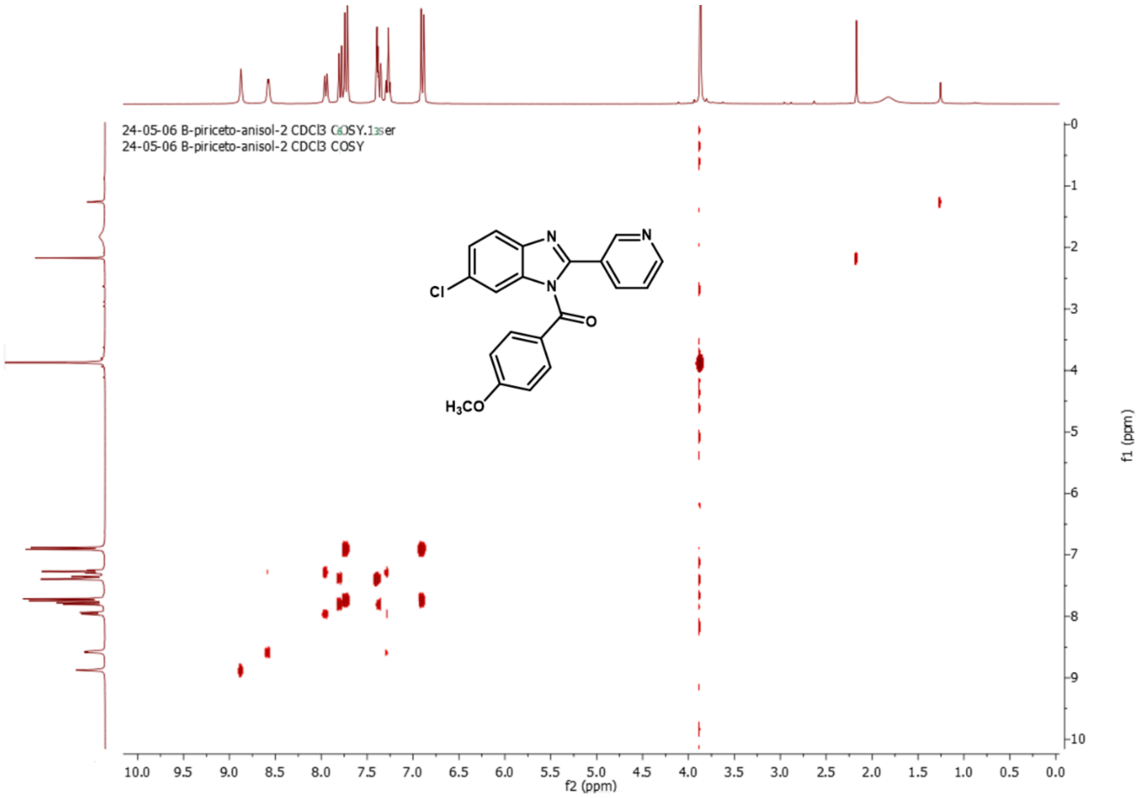

$^{1}H–^{13}C$ – HSQC NMR

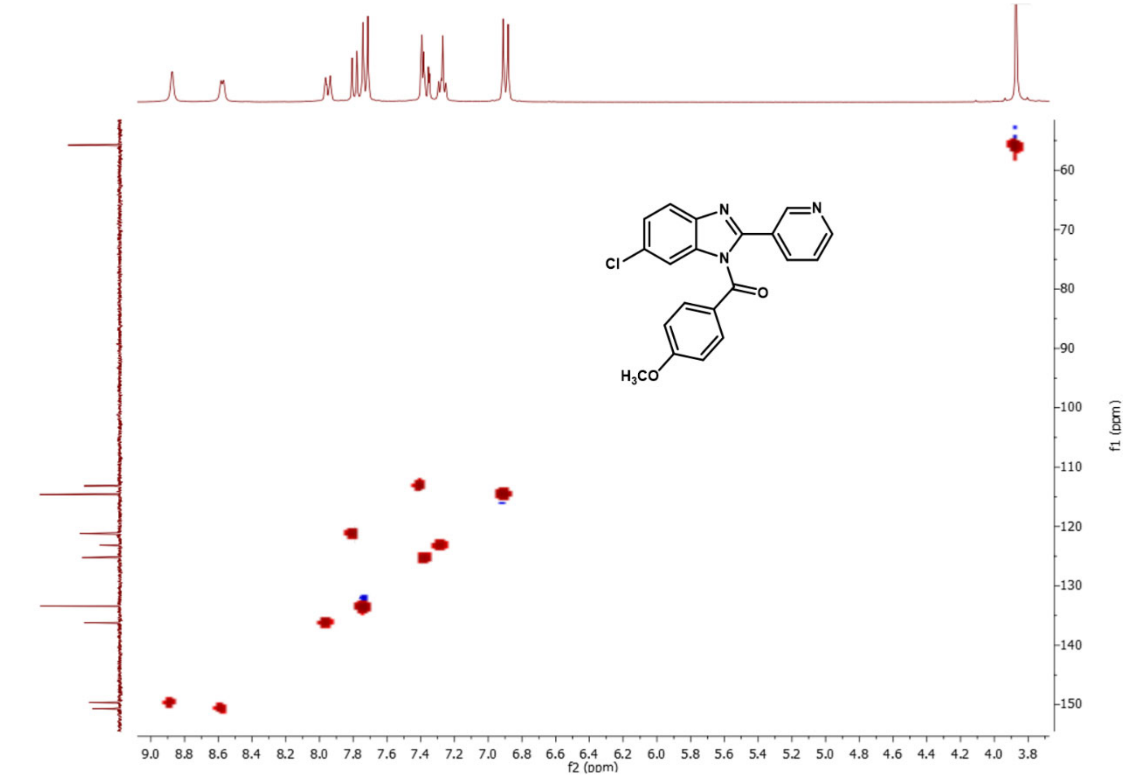

$^{1}H–^{13}C$ – HMBC NMR

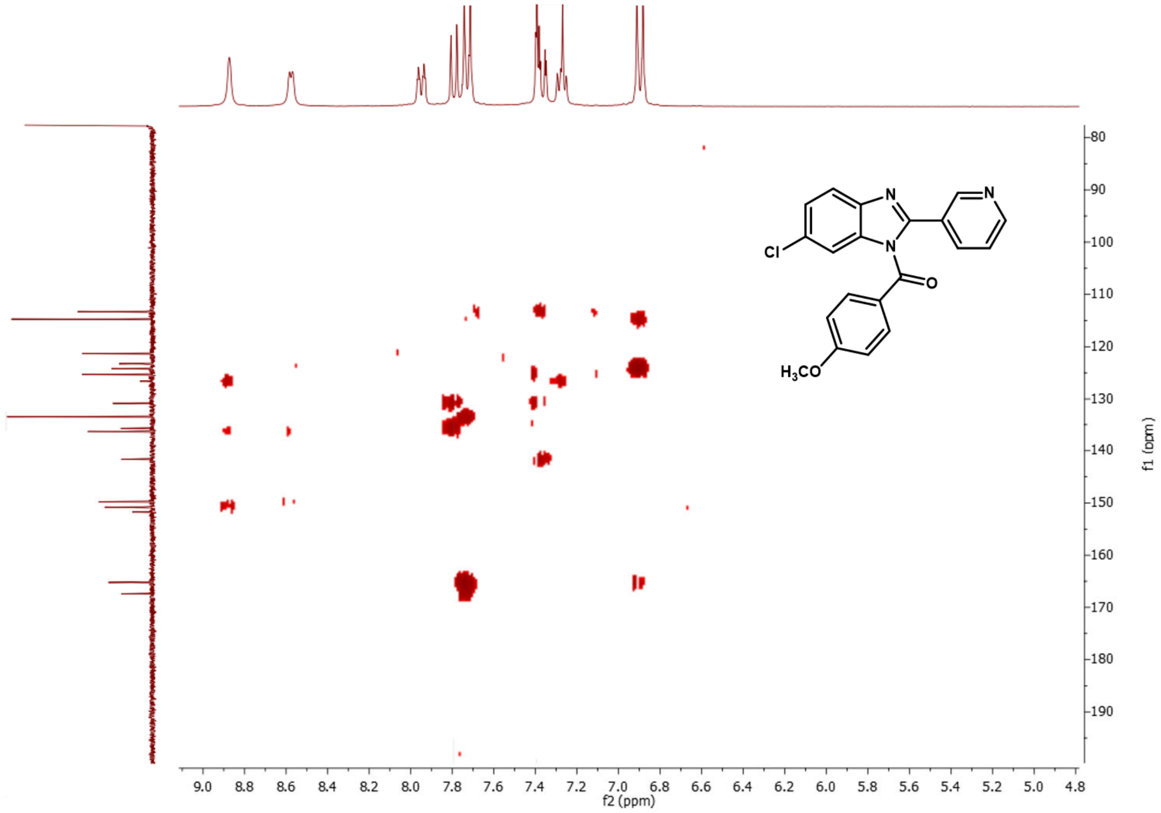

(5-chloro-2-(furan-2-yl)-1*H*-benzo[*d*]imidazol-1-yl)(4-methoxyphenyl)methanone (**3c**)

^{1}H NMR (300 MHz, $CDCl_3$)

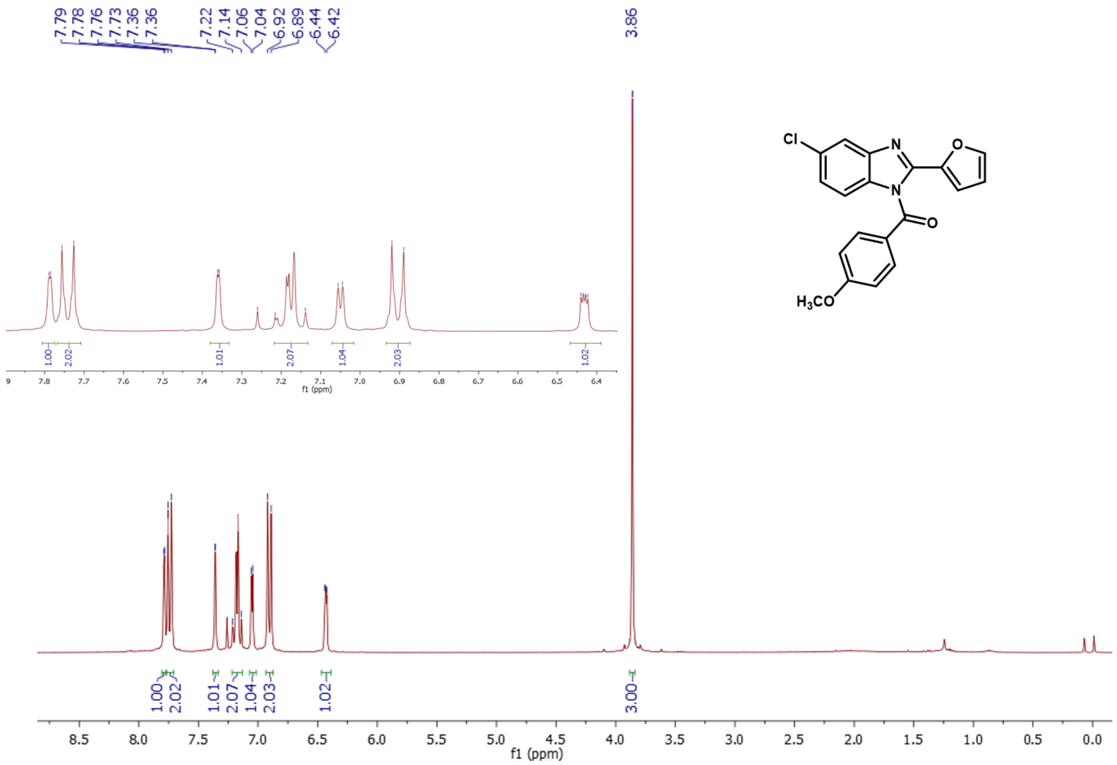

^{13}C NMR (75.5 MHz, $CDCl_3$)

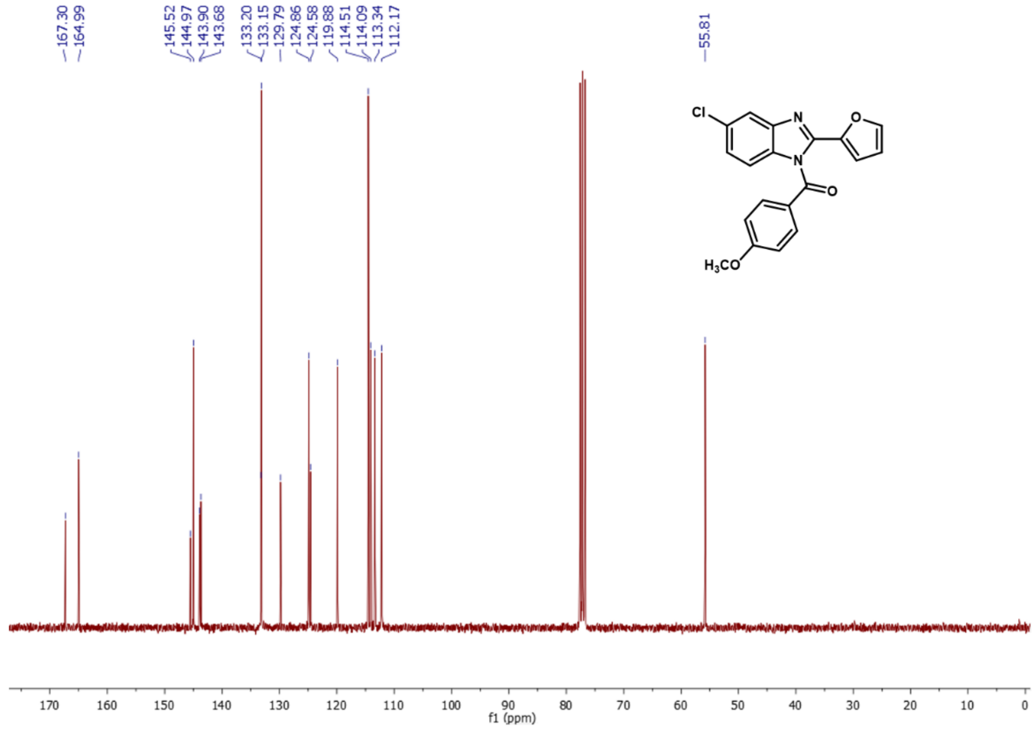

DEPT-135 – NMR

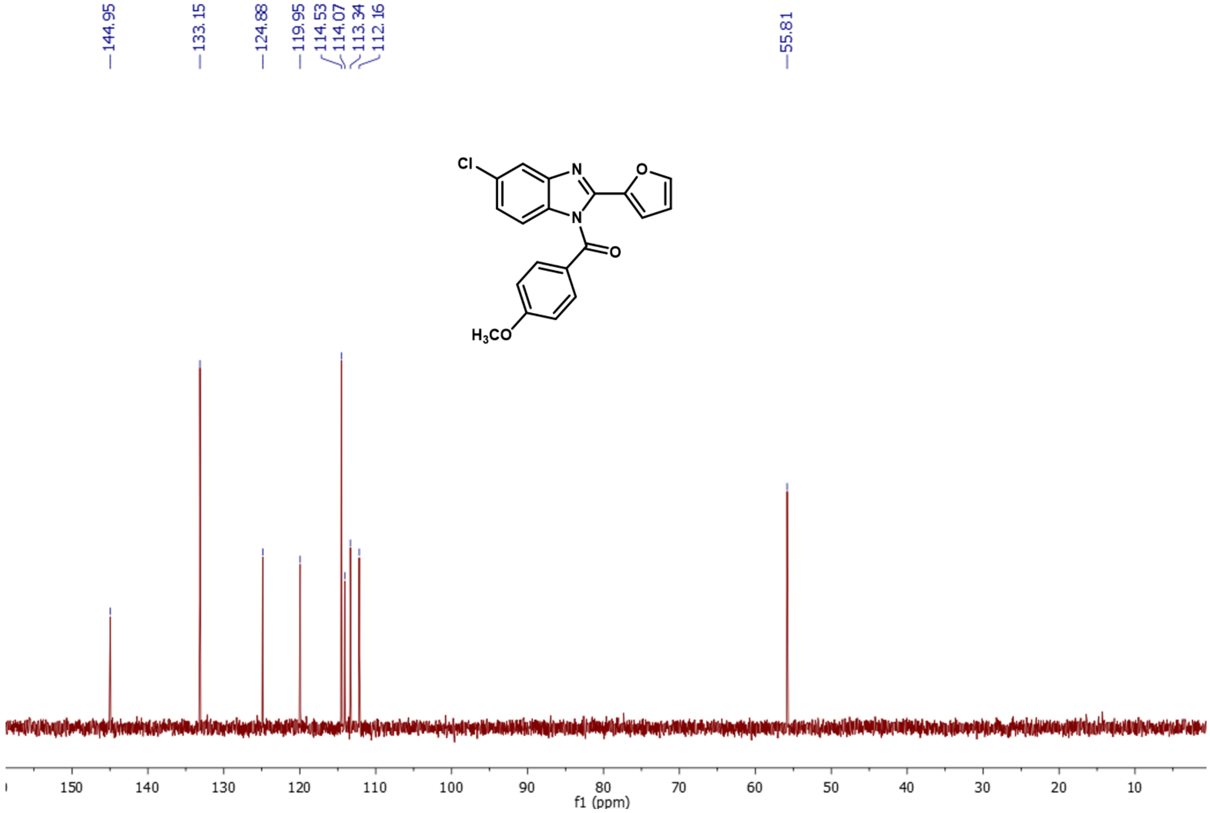

^1H–^1H – COSY NMR

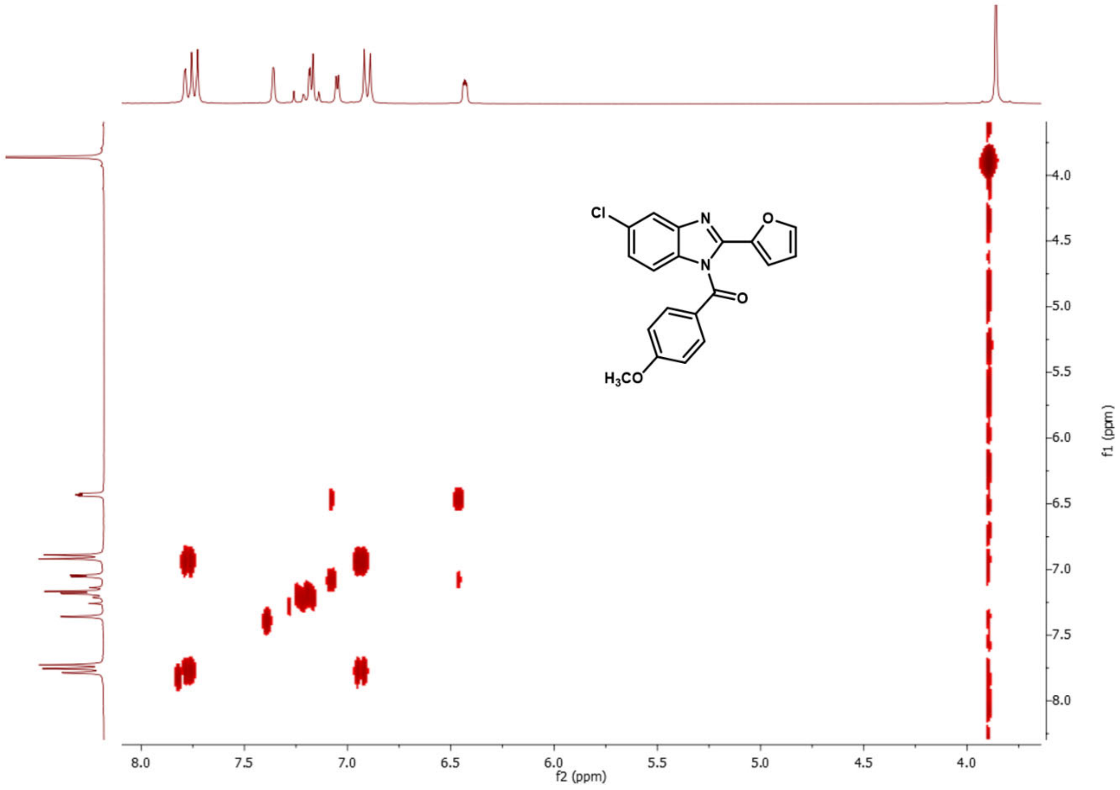

^{1}H–^{13}C – HSQC NMR

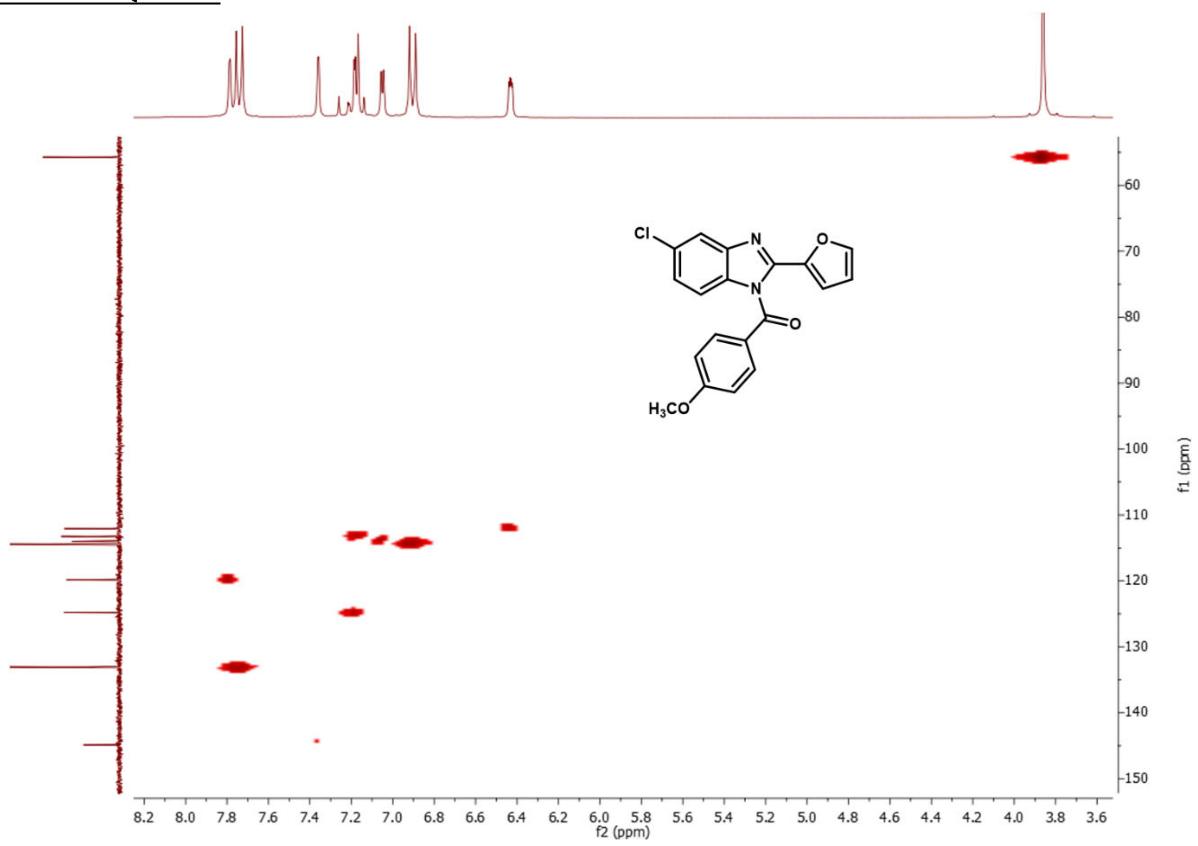

^{1}H–^{13}C – HMBC NMR

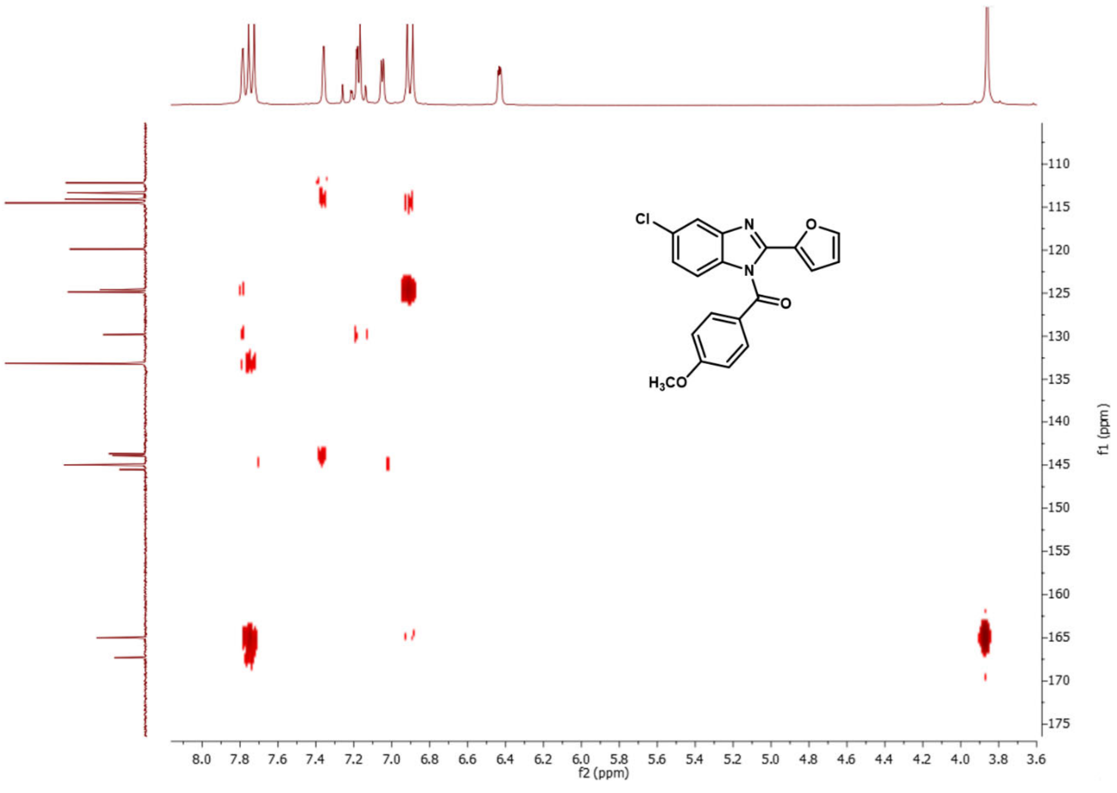

(6-chloro-2-(furan-2-yl)-1*H*-benzo[*d*]imidazol-1-yl)(4-methoxyphenyl)methanone (**3c'**)

^{1}H NMR (300 MHz, $CDCl_3$)

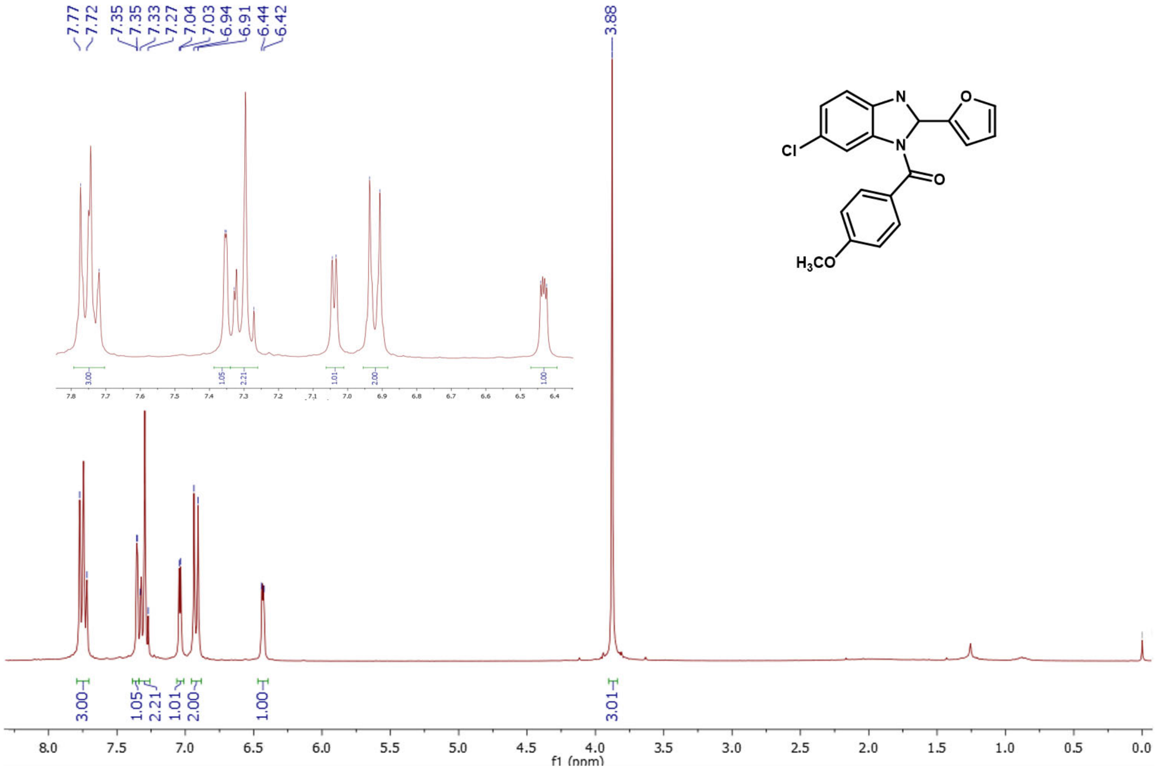

^{13}C NMR (75.5 MHz, $CDCl_3$)

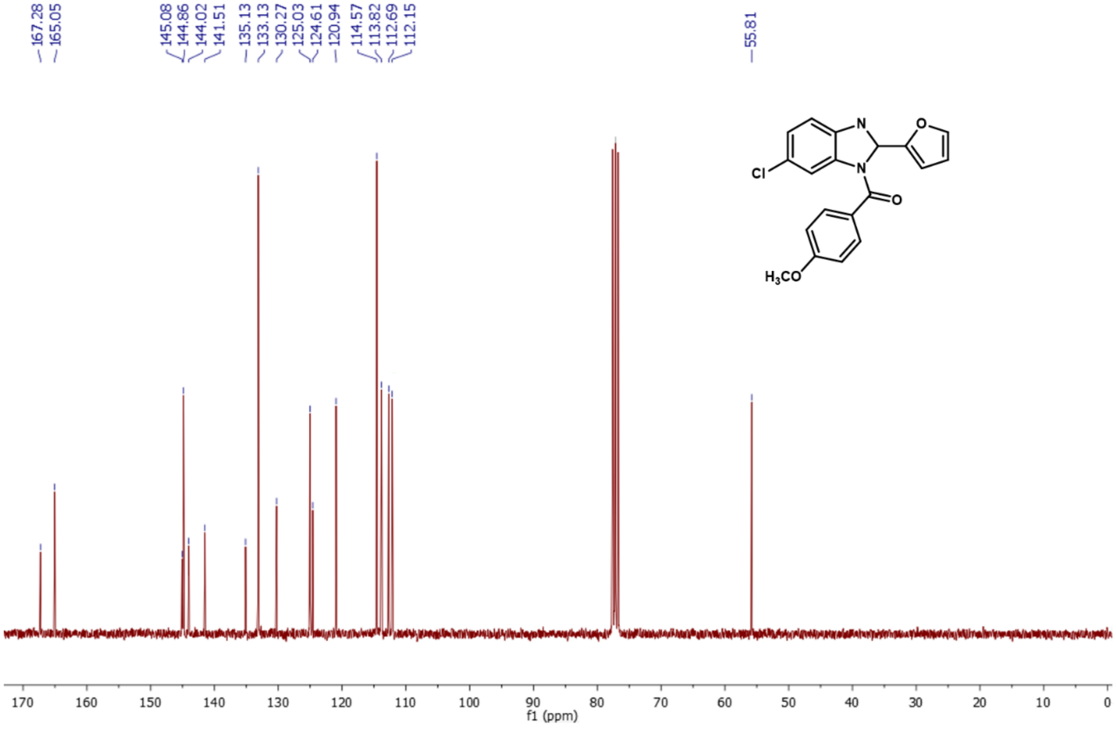

DEPT-135 – NMR

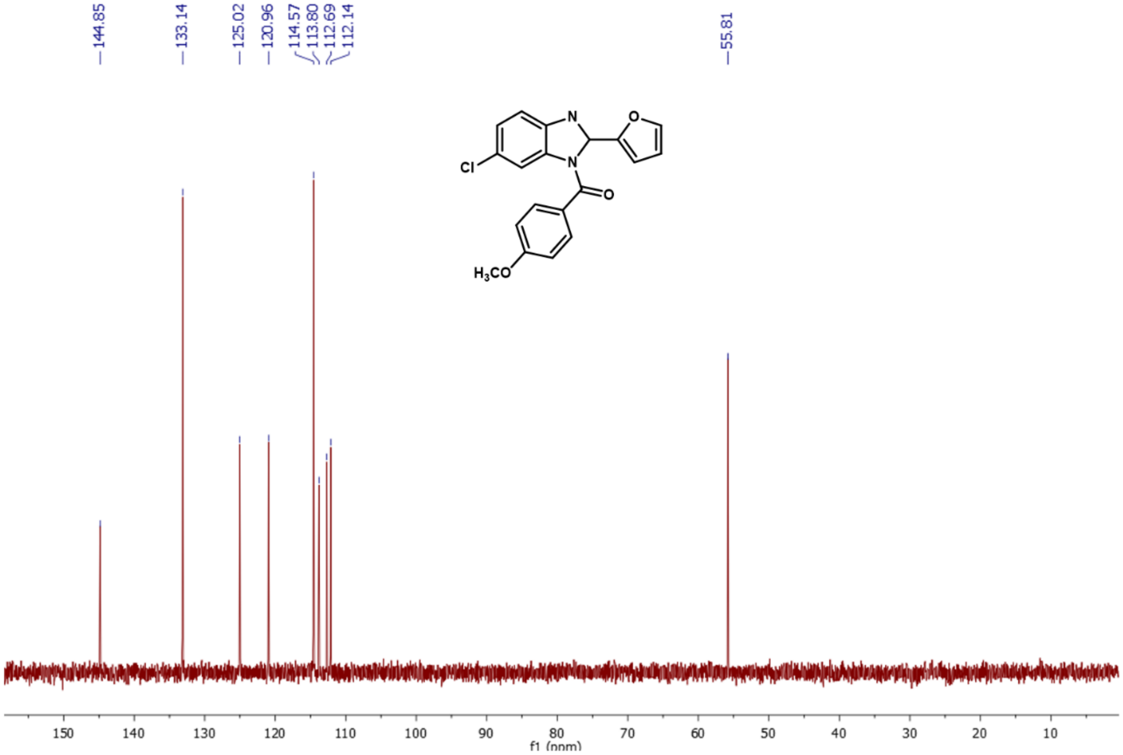

^{1}H–^{1}H – COSY NMR

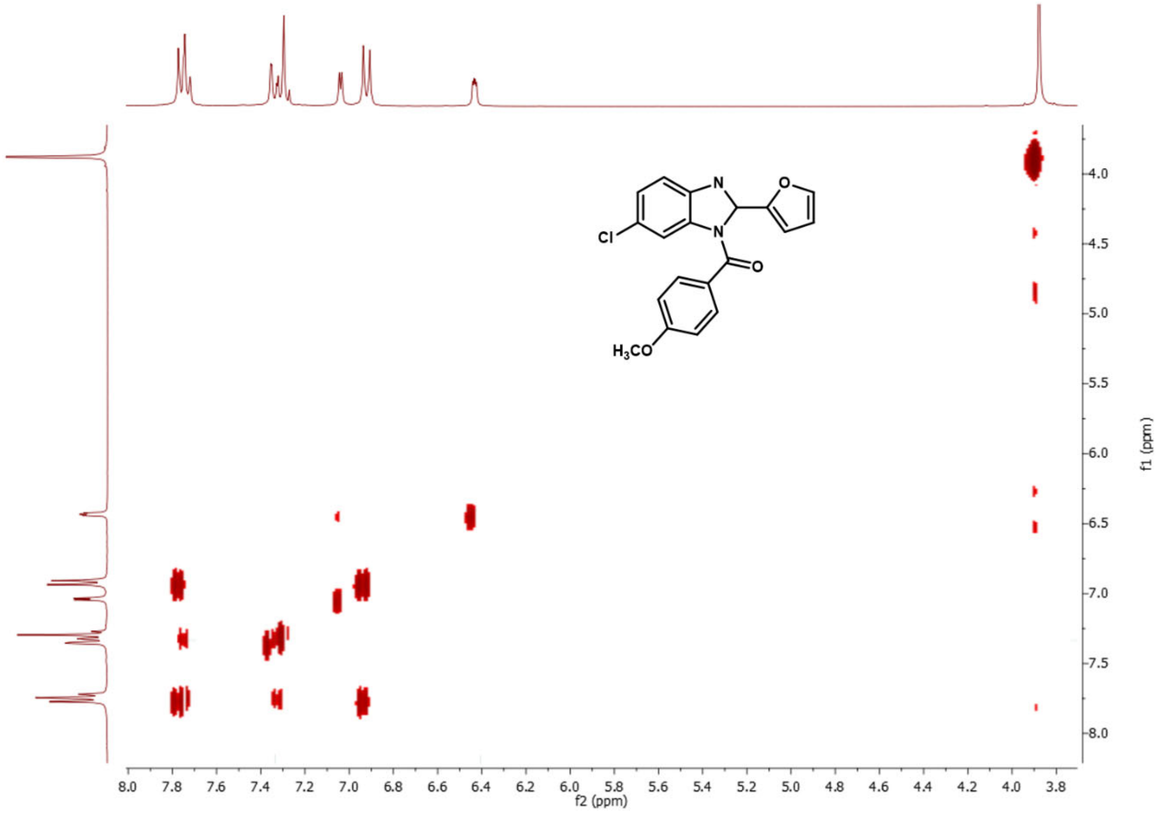

^{1}H–^{13}C – HSQC NMR

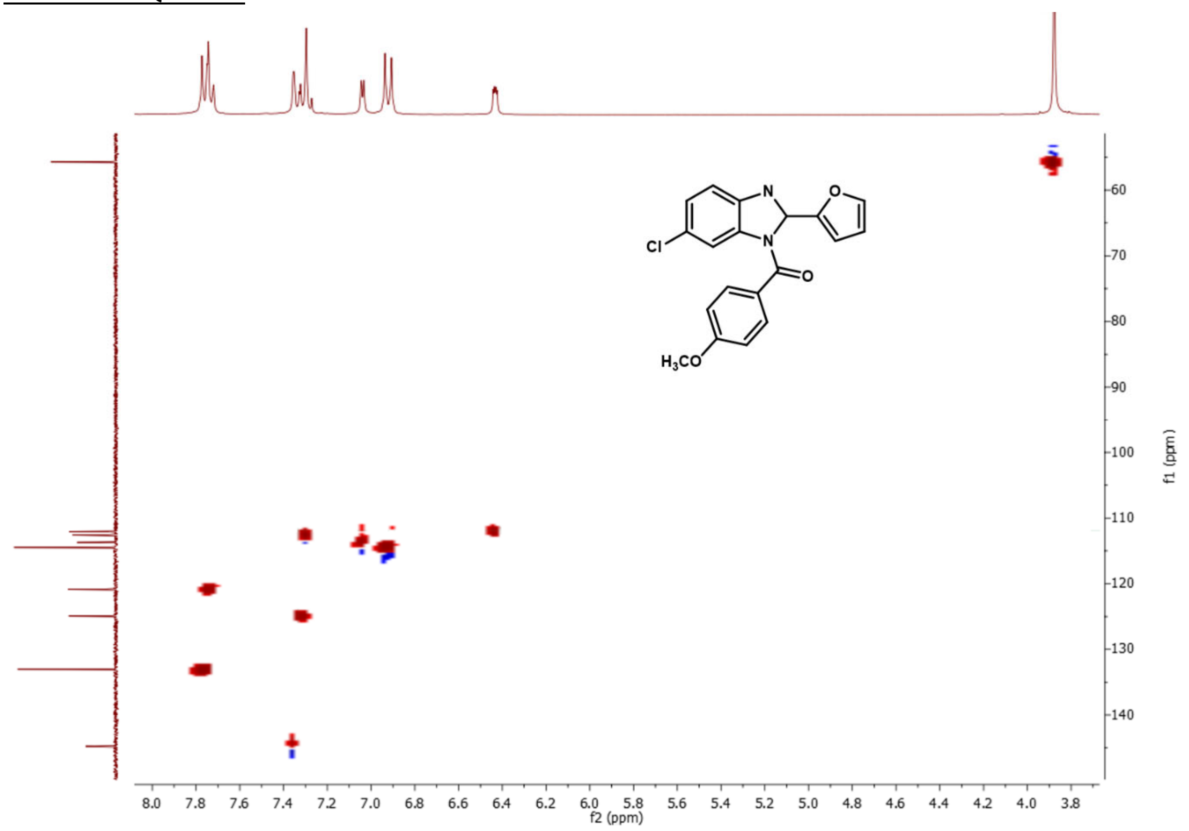

^{1}H–^{13}C – HMBC NMR

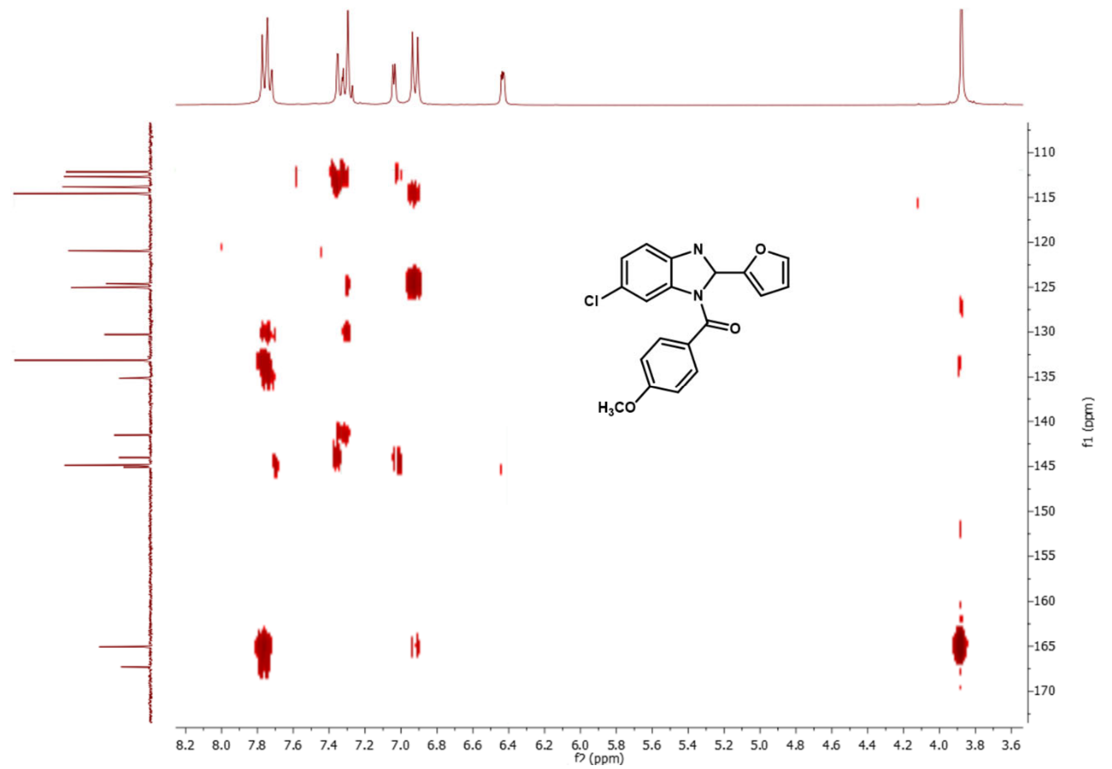

(5-chloro-2-(isoxazol-3-yl)-1*H*-benzo[*d*]imidazol-1-yl)(4-methoxyphenyl)methanone (**3d**)

^{1}H NMR (300 MHz, DMSO-d[6])

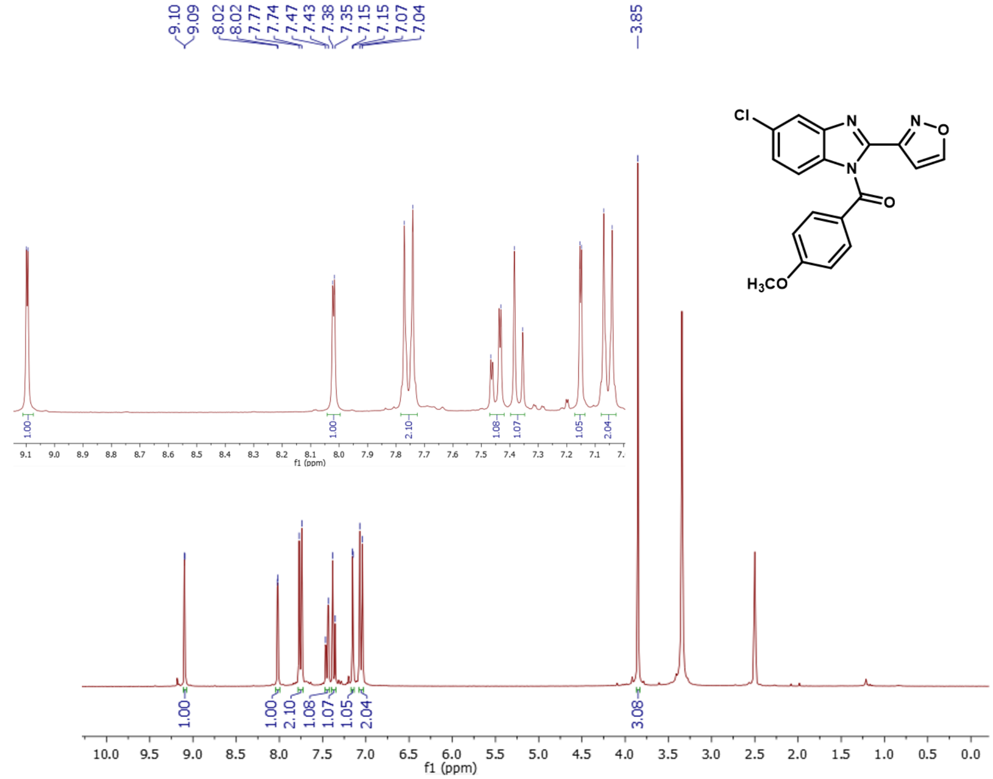

^{13}C NMR (75.5 MHz, DMSO-d[6])

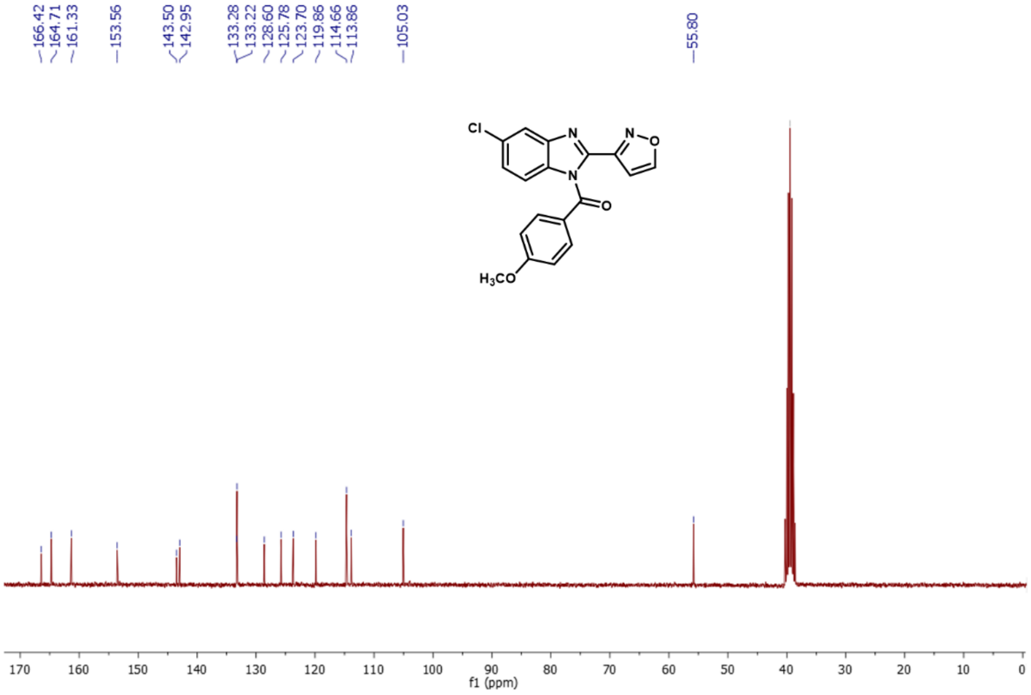

DEPT-135 – NMR

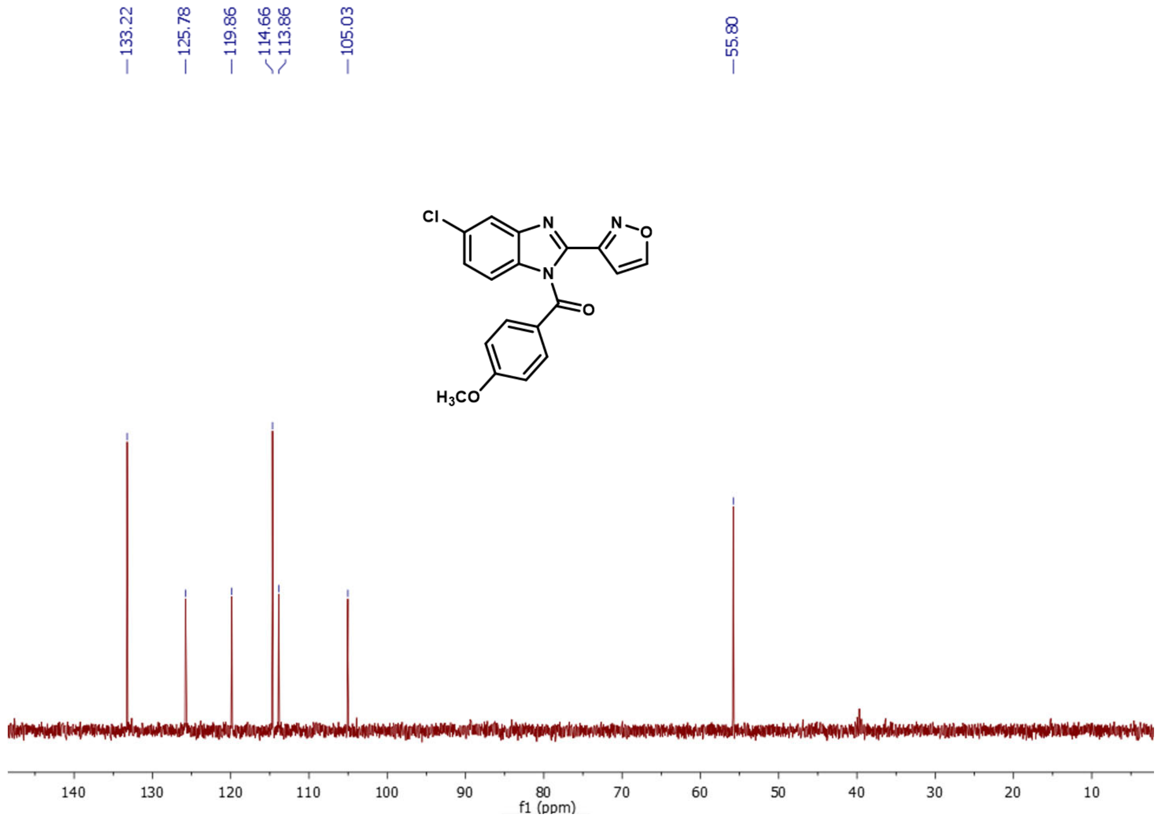

^1H–^1H – COSY NMR

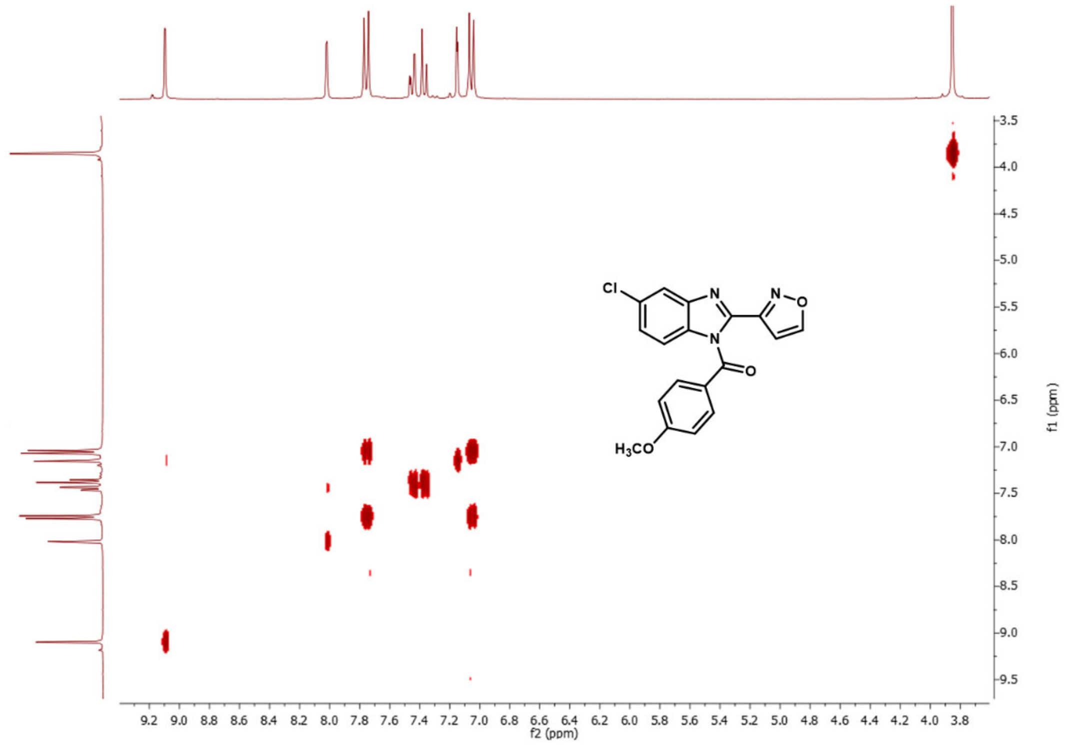

^{1}H–^{13}C – HSQC NMR

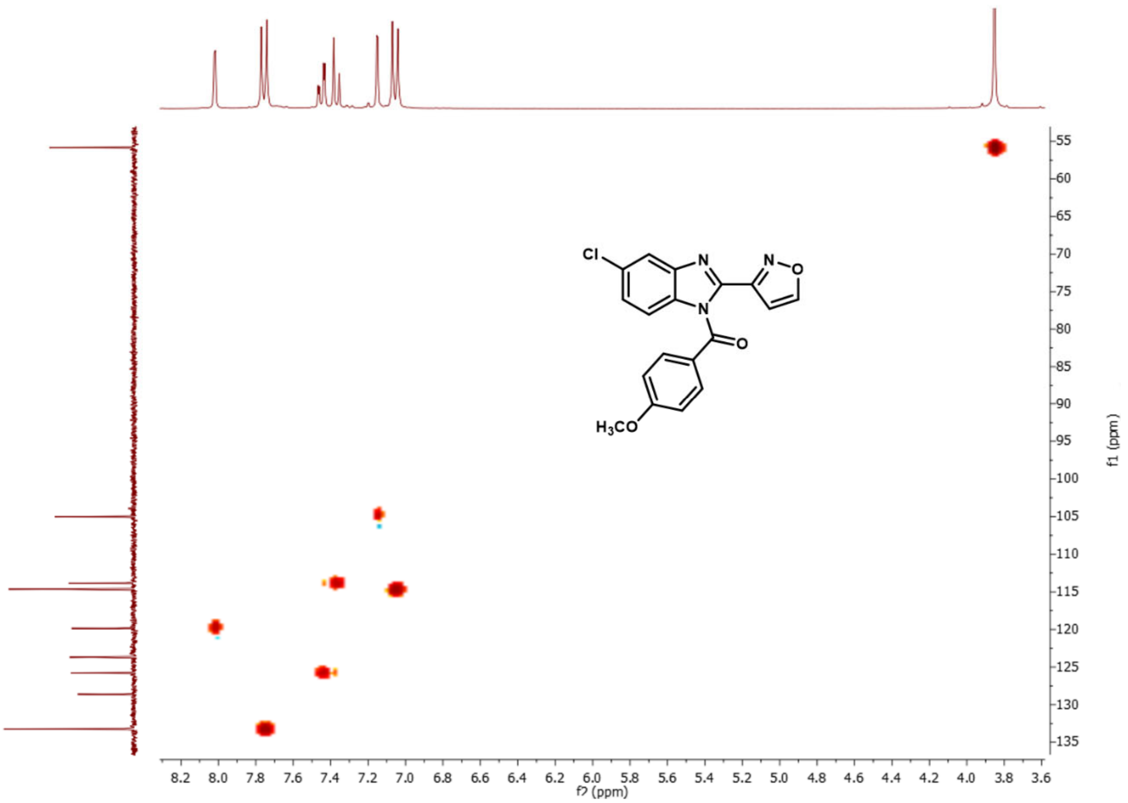

^{1}H–^{13}C – HMBC NMR

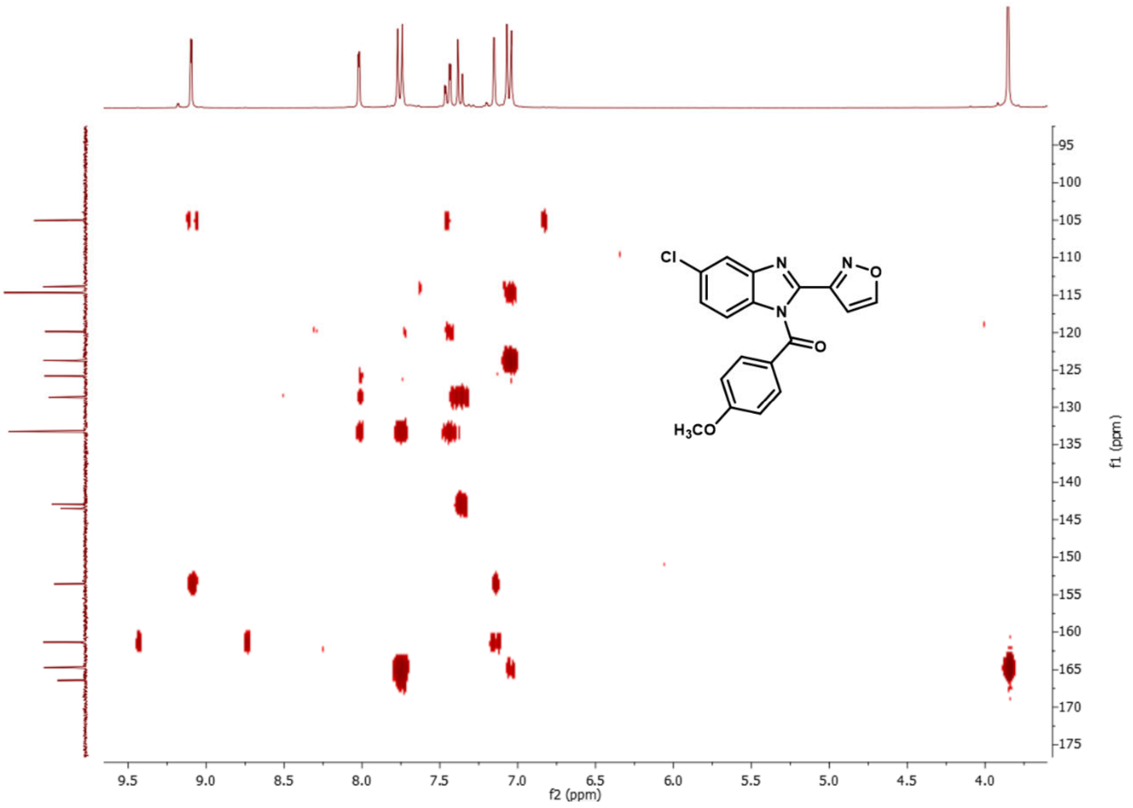

(6-chloro-2-(isoxazol-3-yl)-1*H*-benzo[*d*]imidazol-1-yl)(4-methoxyphenyl)methanone (**3d'**)

^{1}H NMR (300 MHz, DMSO-d^{6})

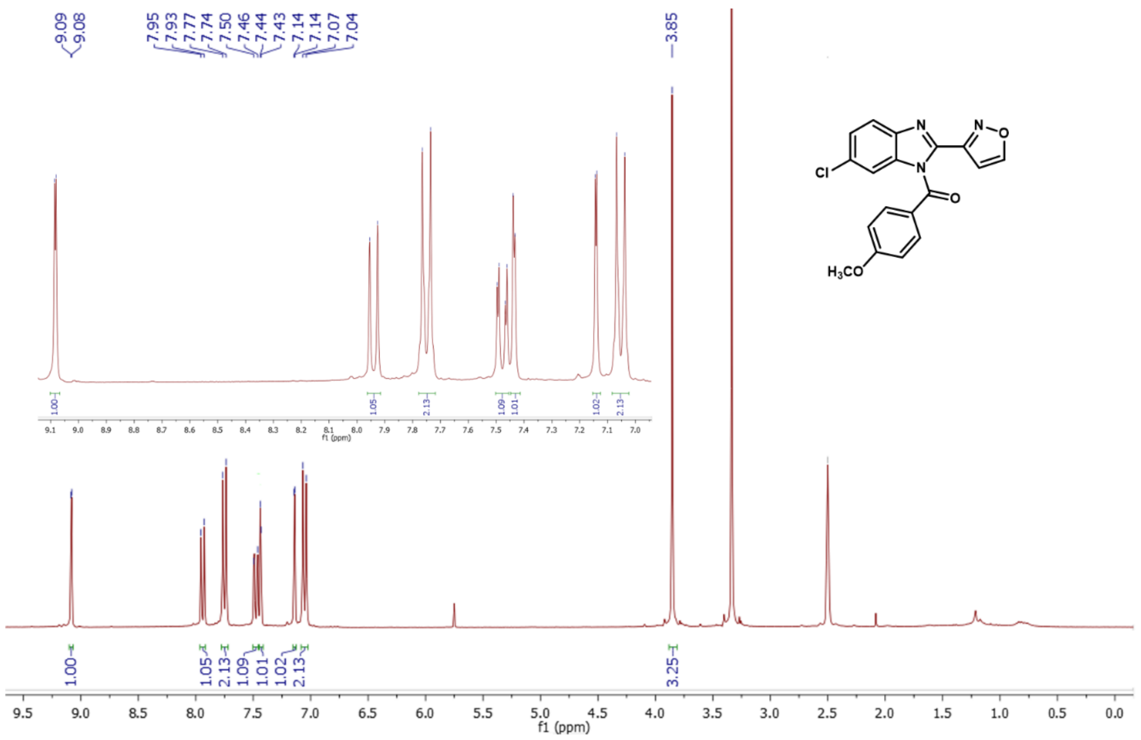

^{13}C NMR (75.5 MHz, DMSO-d^{6})

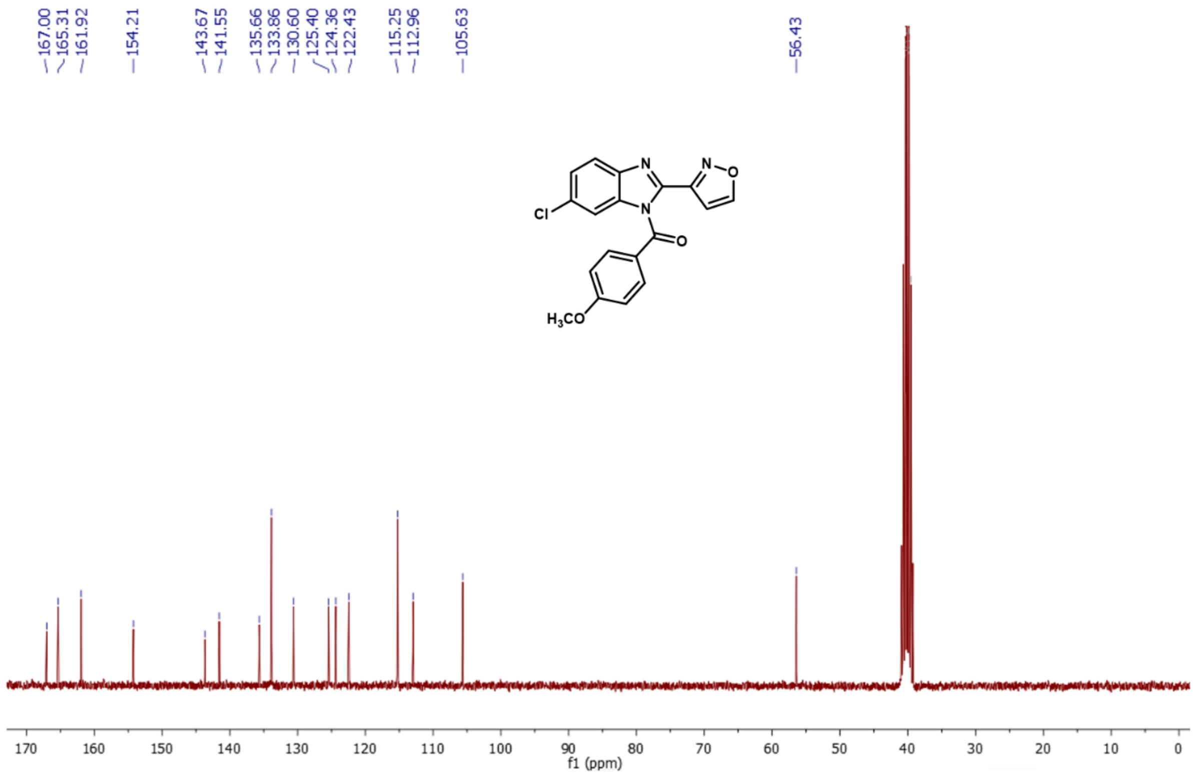

DEPT-135 – NMR

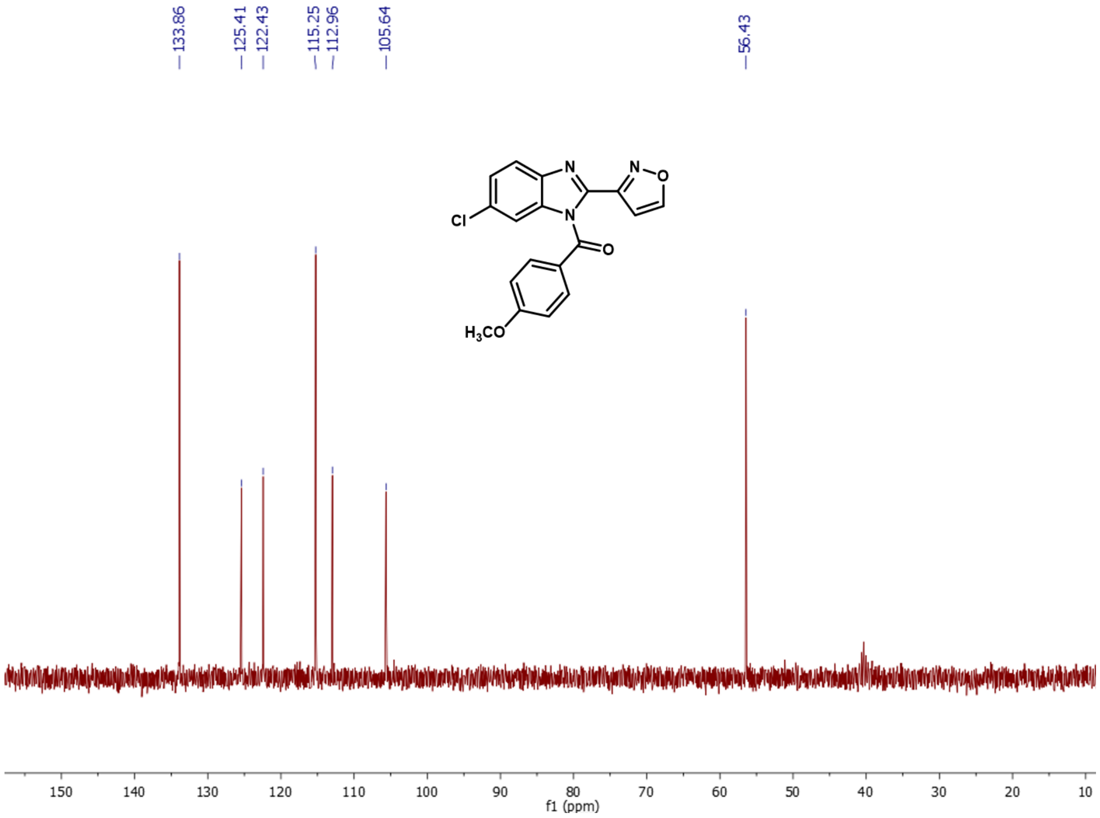

^{1}H–^{1}H – COSY NMR

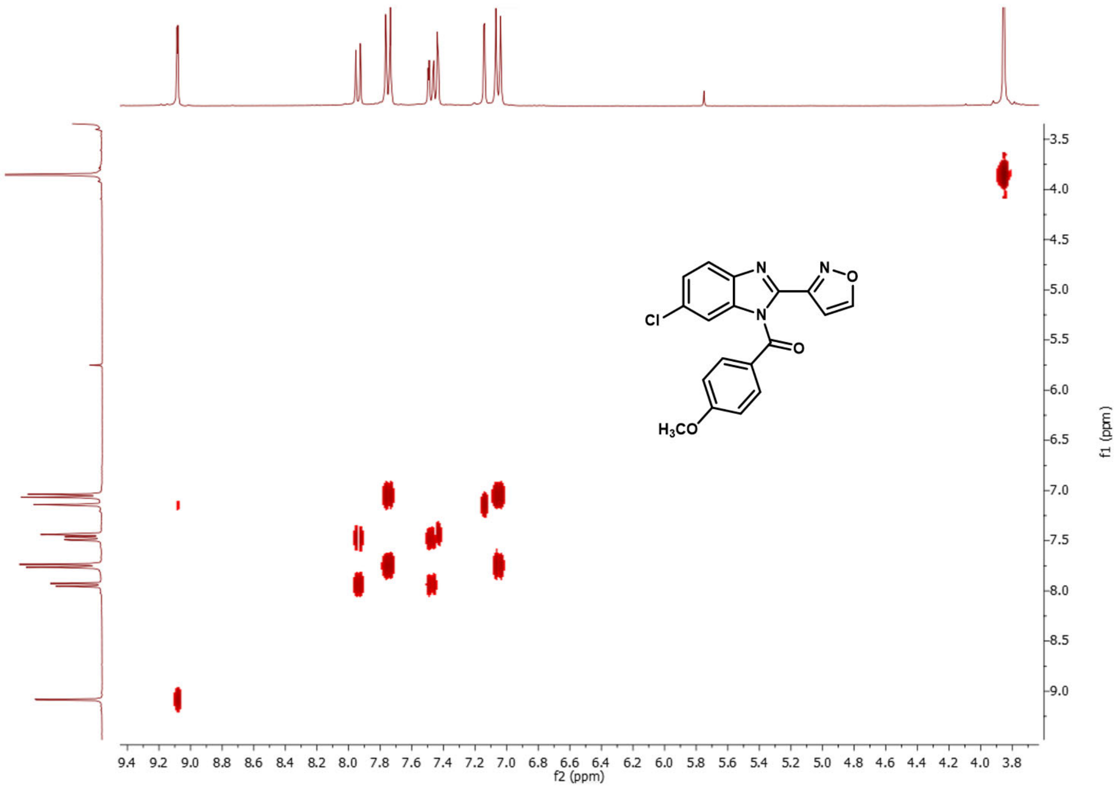

^{1}H–^{13}C – HSQC NMR

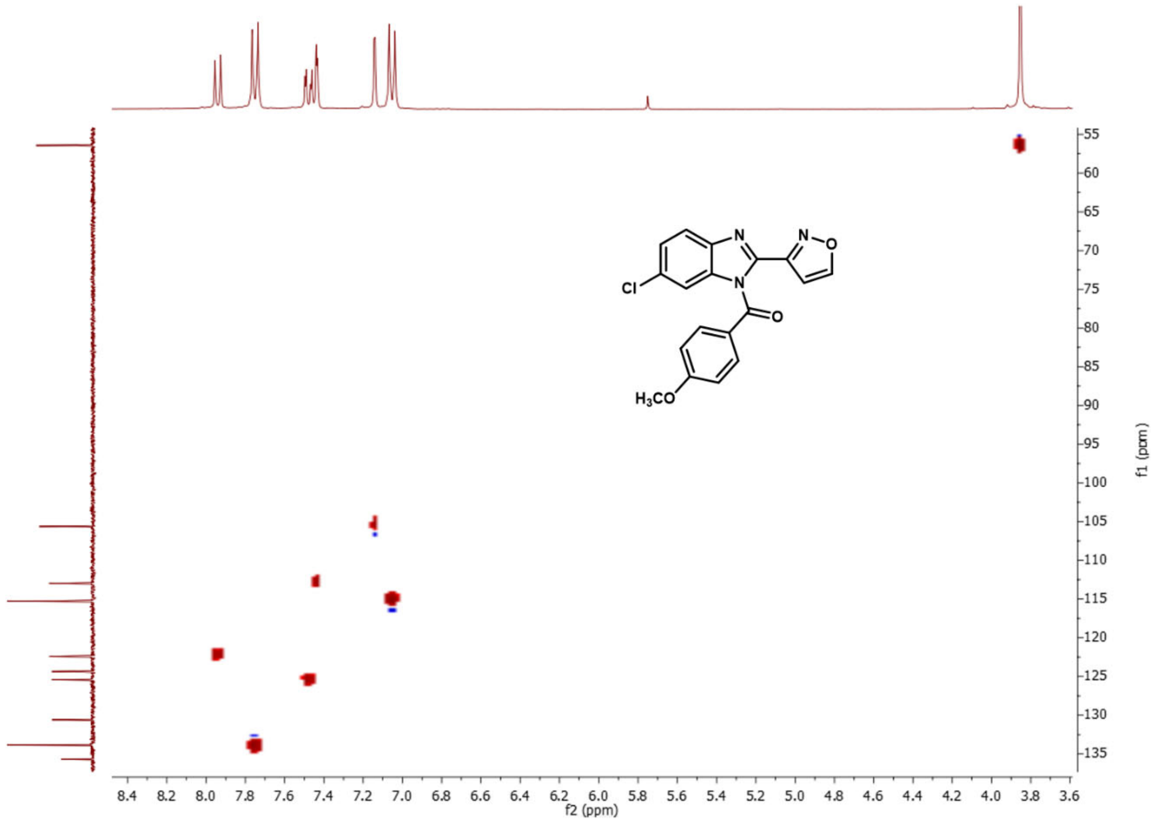

^{1}H–^{13}C – HMBC NMR

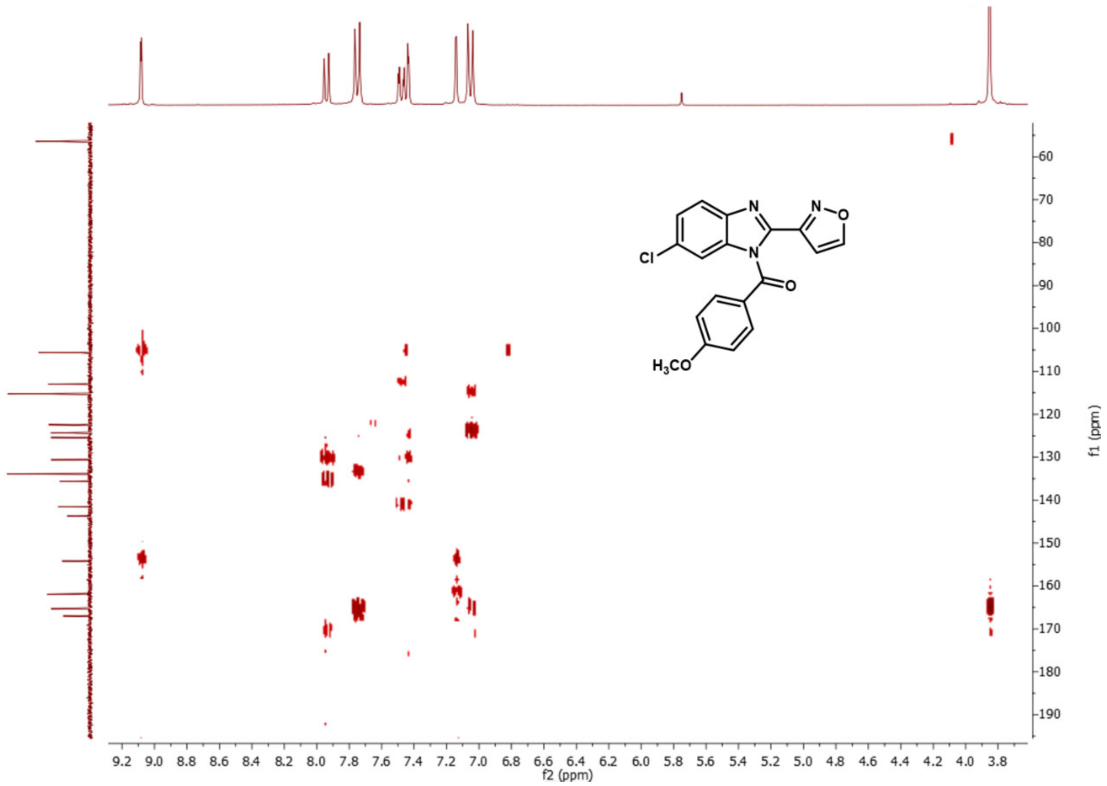

5-chloro-1-(4-methoxybenzyl)-2-(3-methoxyphenyl)-1*H*-benzo[*d*]imidazolemethanone (**4a**)

^{1}H NMR (300 MHz, $CDCl_3$)

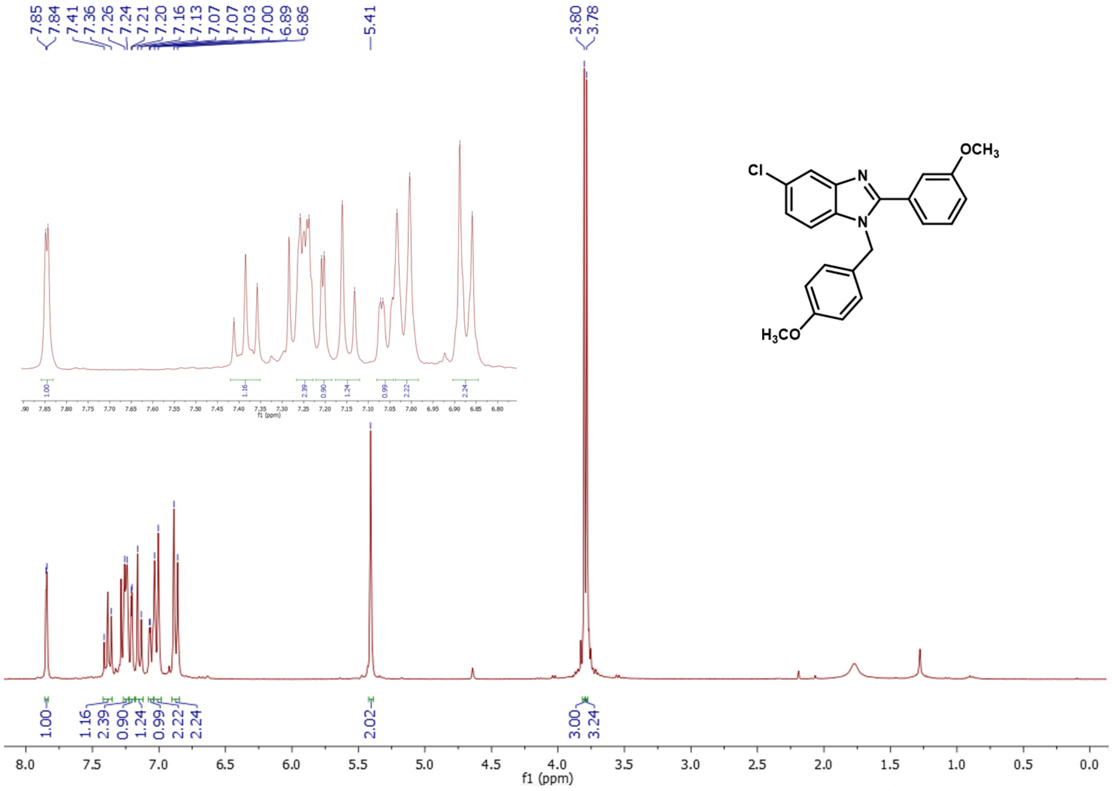

^{13}C NMR (75.5 MHz, $CDCl_3$)

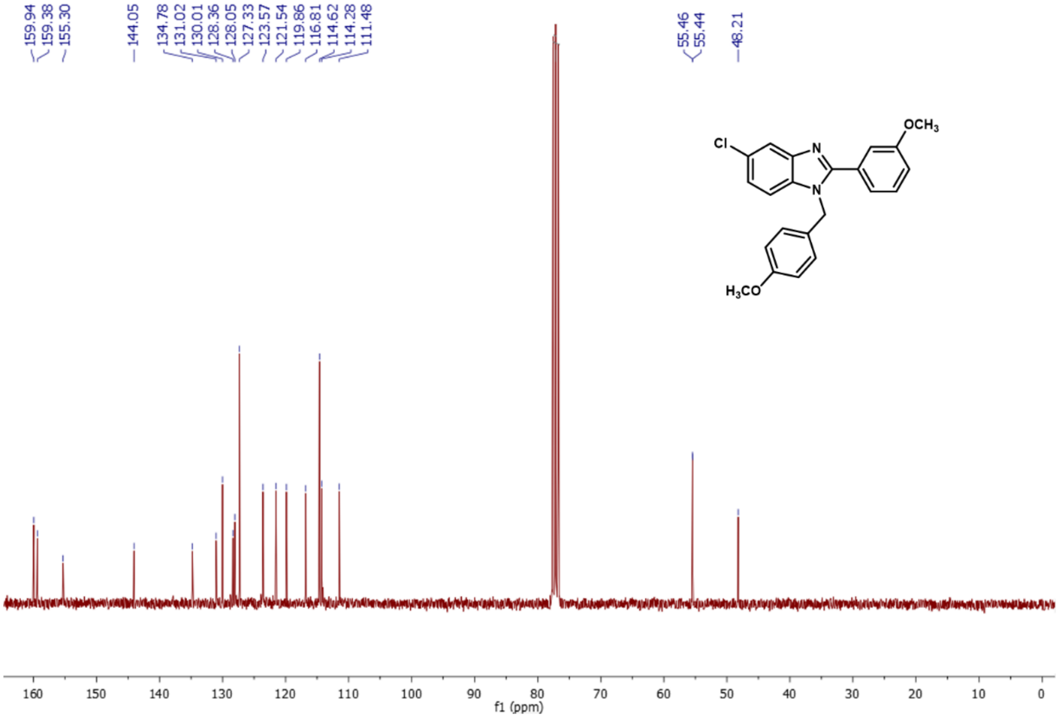

DEPT-135 – NMR

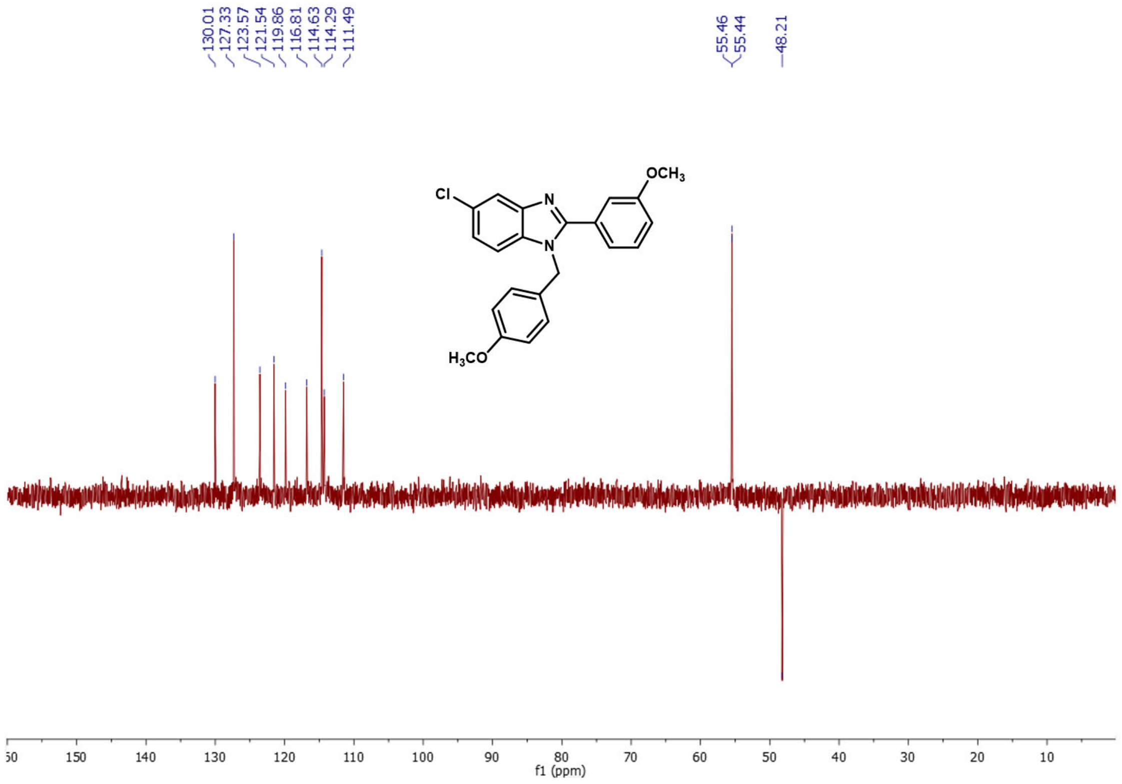

^{1}H–^{1}H – COSY NMR

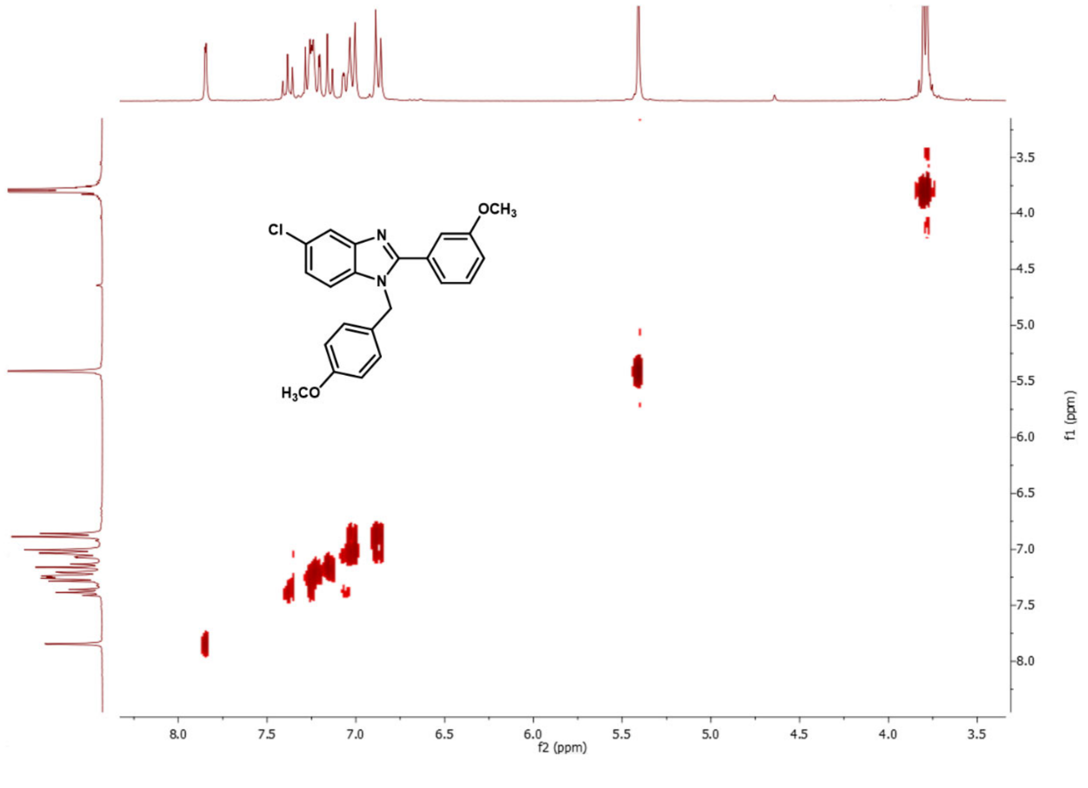

^1H–^{13}C – HSQC NMR

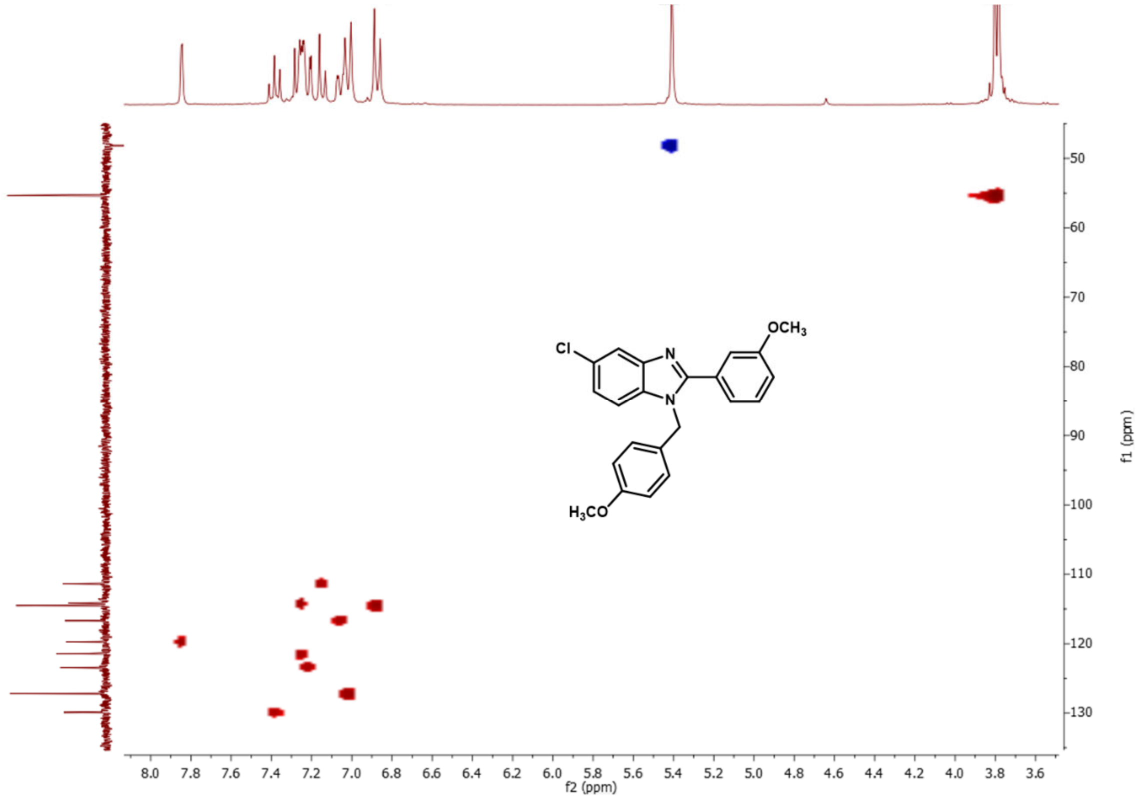

^1H–^{13}C – HMBC NMR

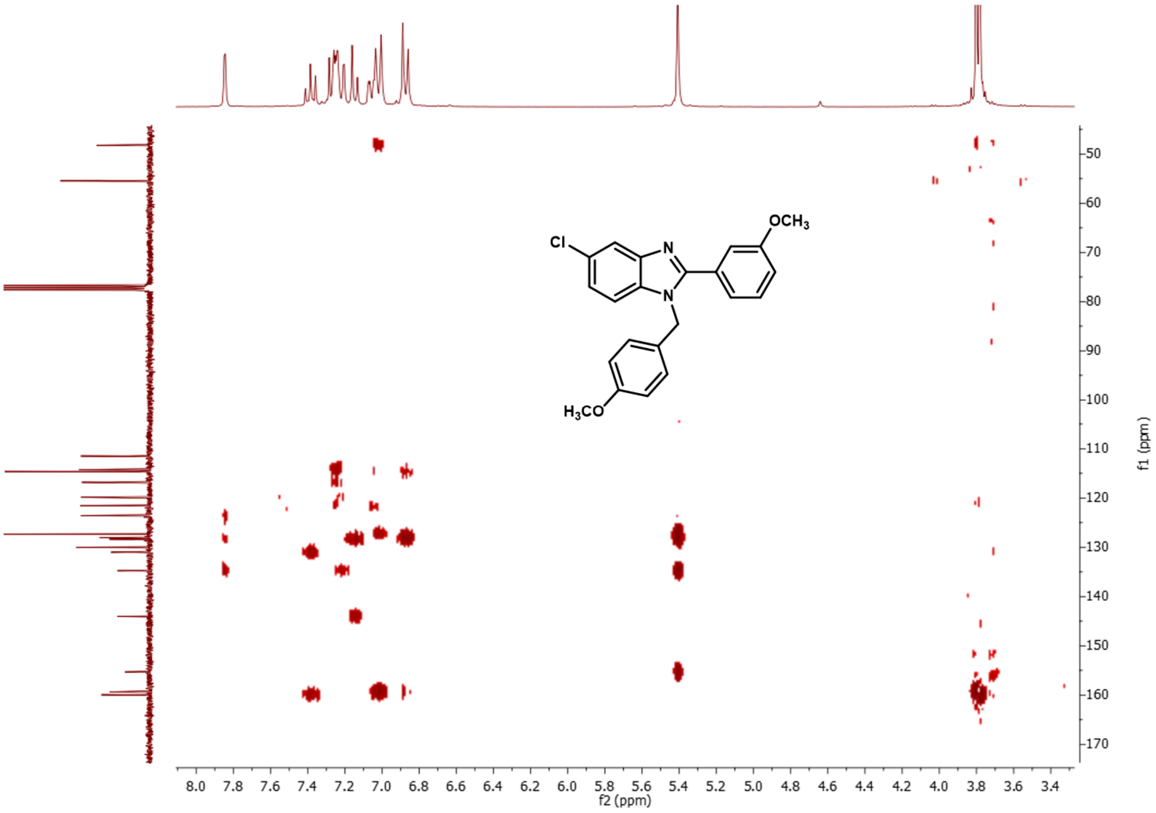

6-chloro-1-(4-methoxybenzyl)-2-(3-methoxyphenyl)-1*H*-benzo[*d*]imidazolemethanone (**4a'**)

^{1}H NMR (300 MHz, $CDCl_3$)

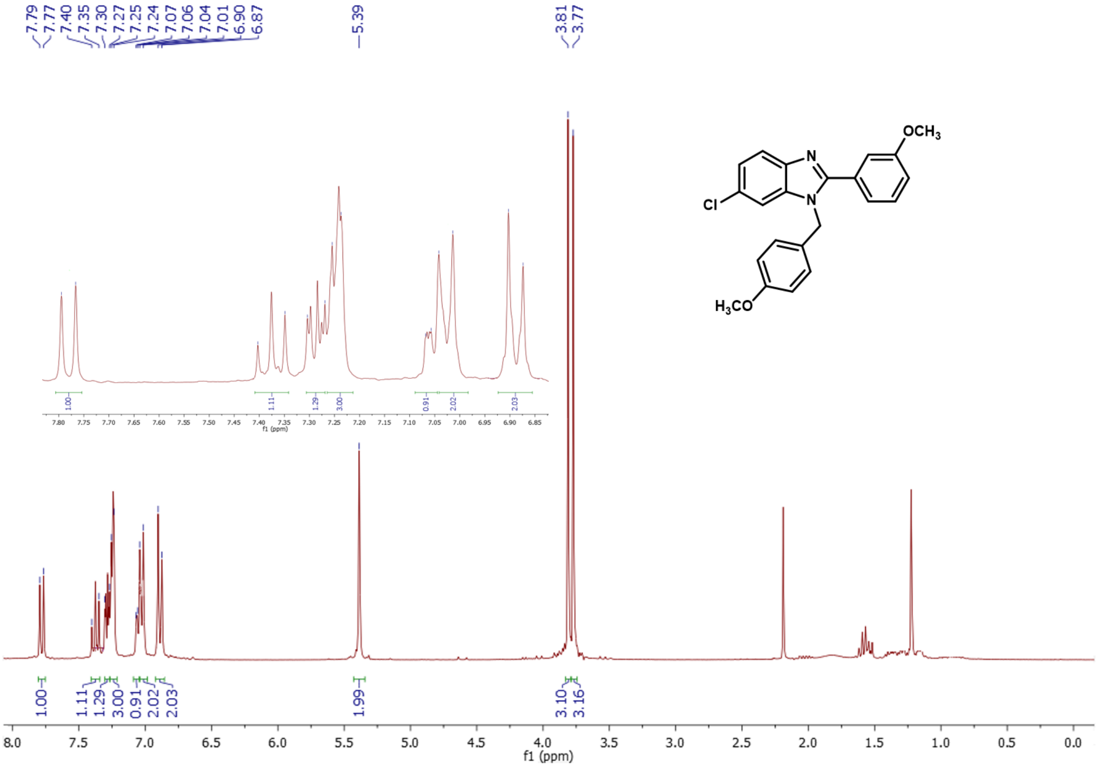

^{13}C NMR (75.5 MHz, $CDCl_3$)

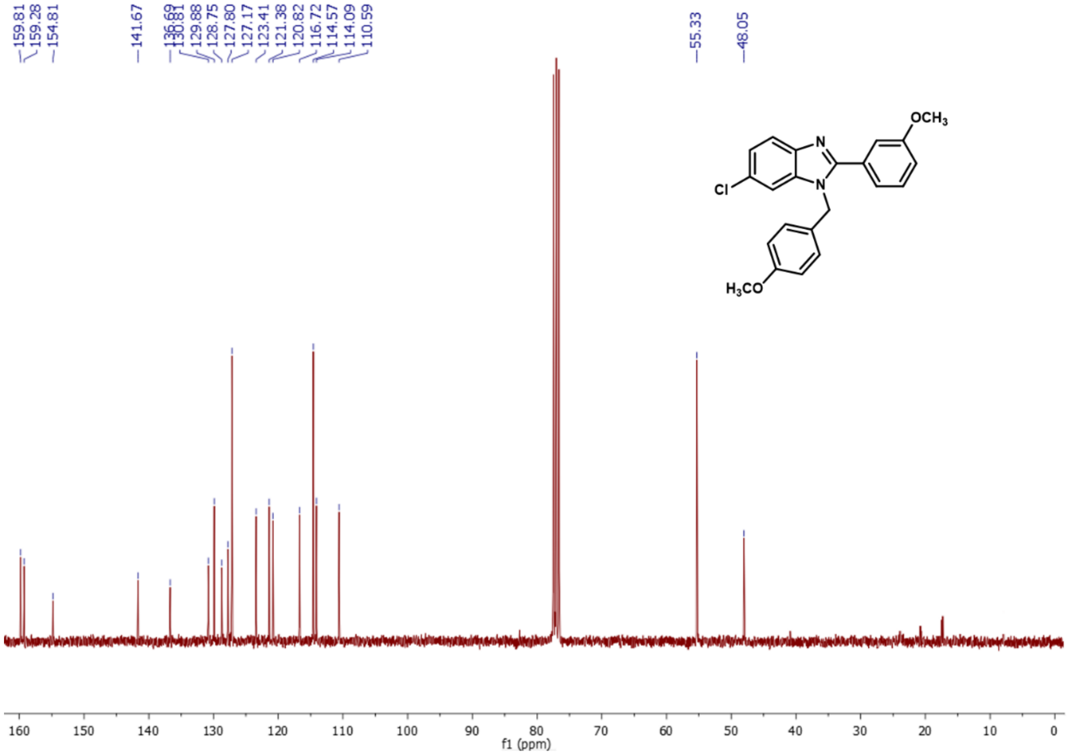

DEPT-135 – NMR

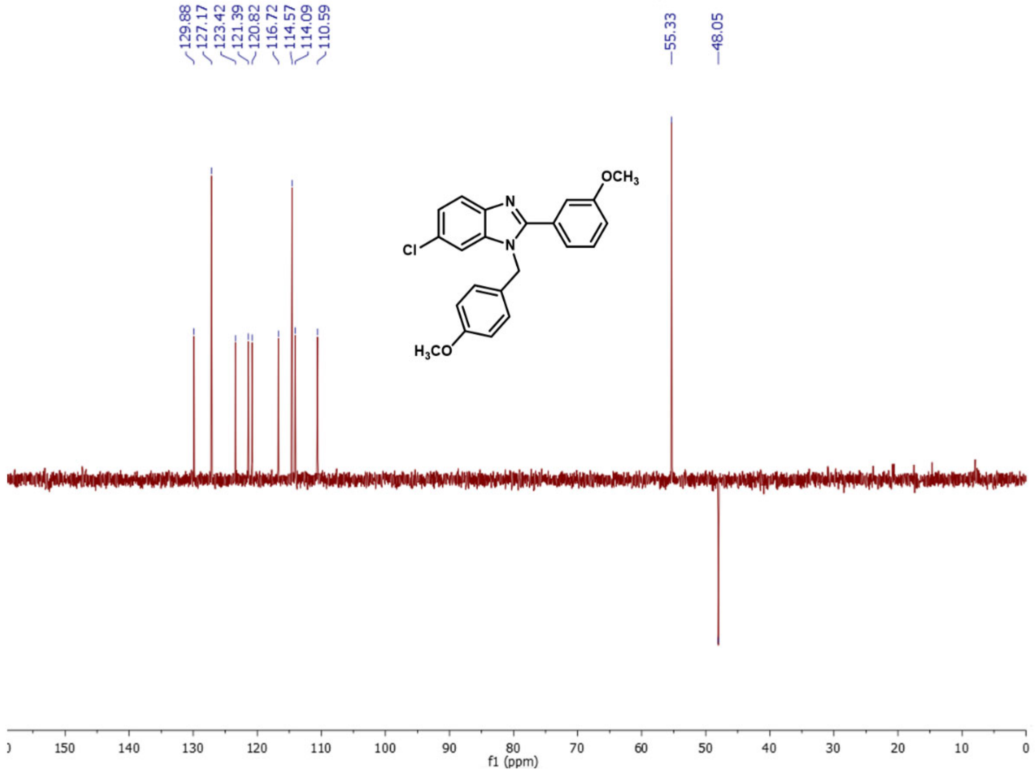

^1H–^1H – COSY NMR

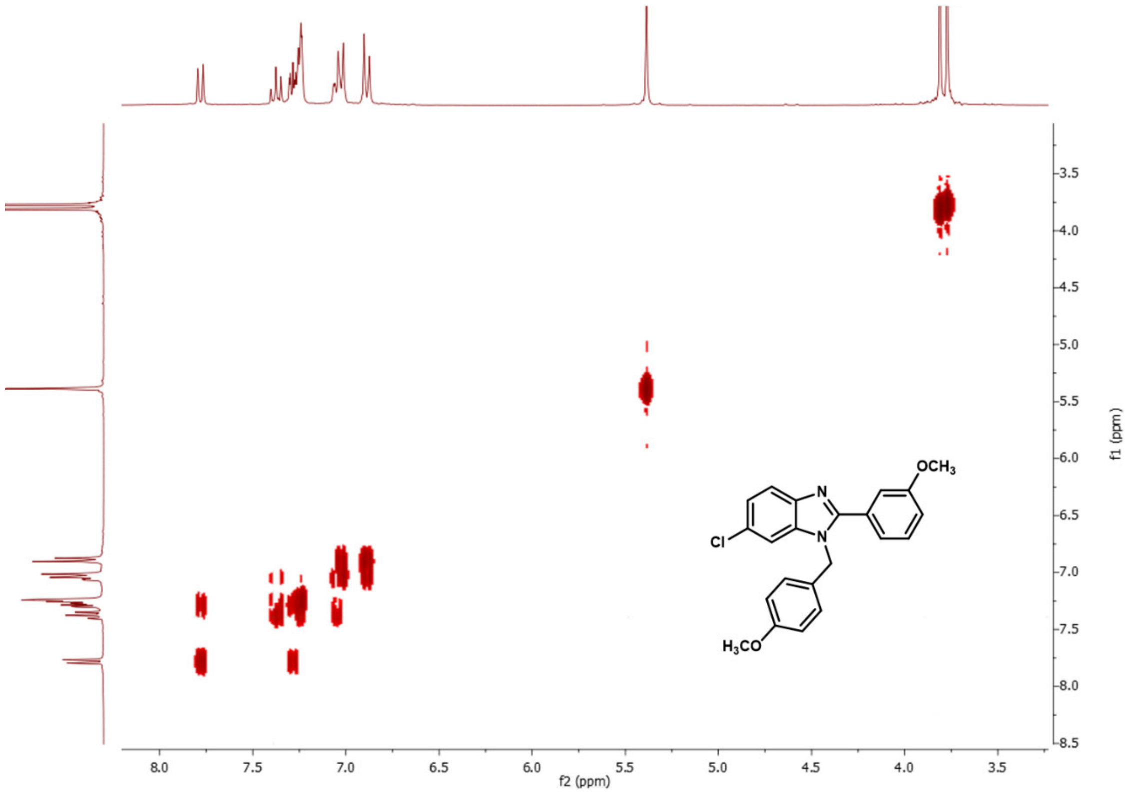

^{1}H–^{13}C – HSQC NMR

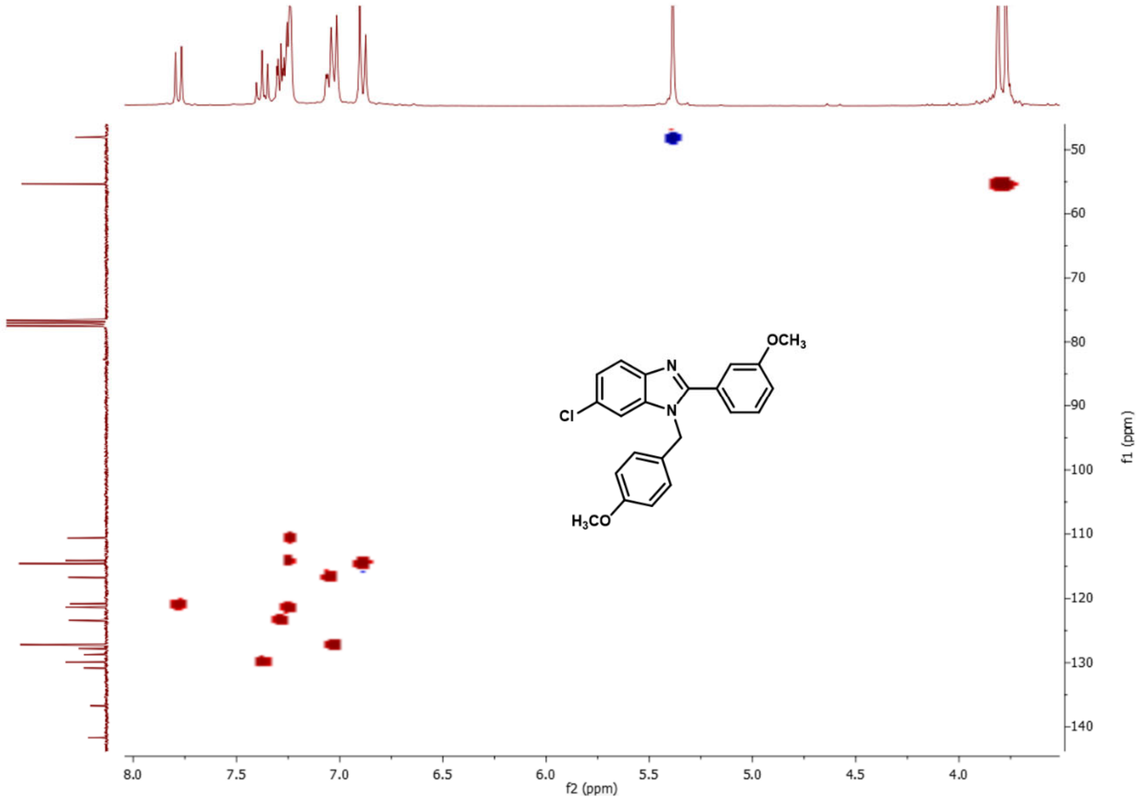

^{1}H–^{13}C – HMBC NMR

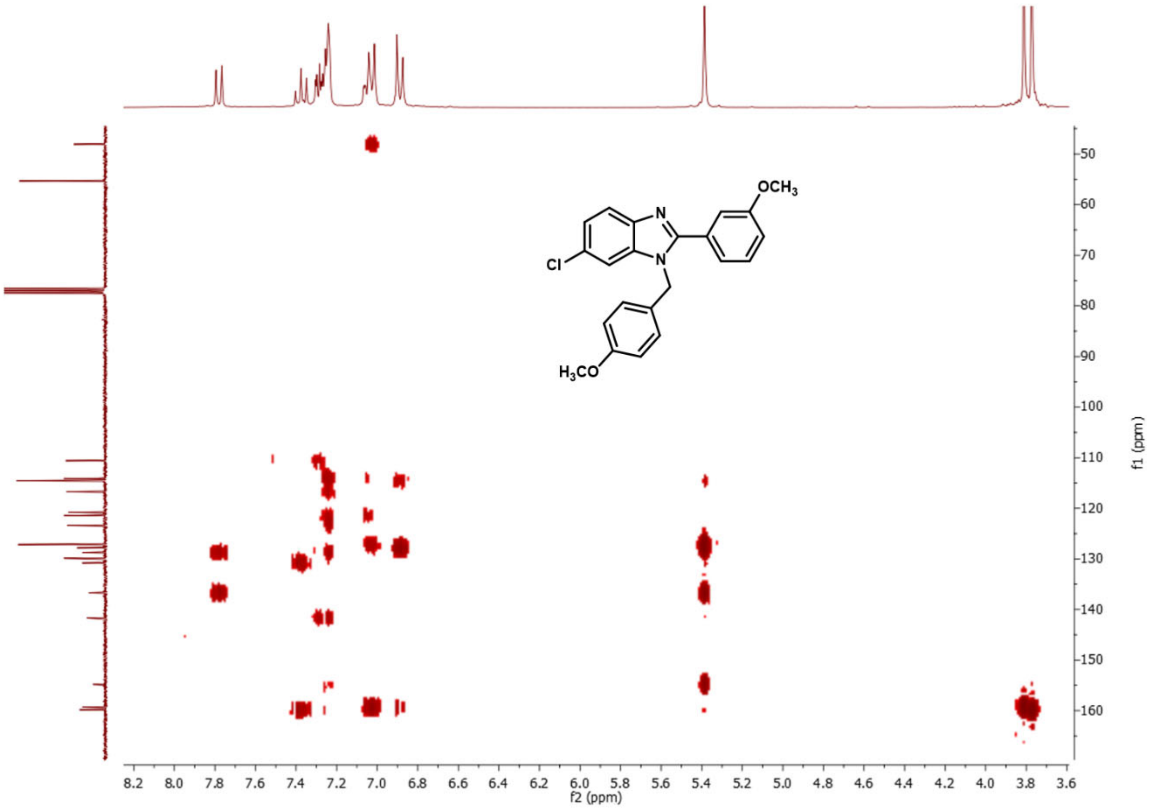

5-chloro-1-(4-methoxybenzyl)-2-(pyridin-3-yl)-1*H*-benzo[*d*]imidazole (**4b**)

^{1}H NMR (300 MHz, $CDCl_3$)

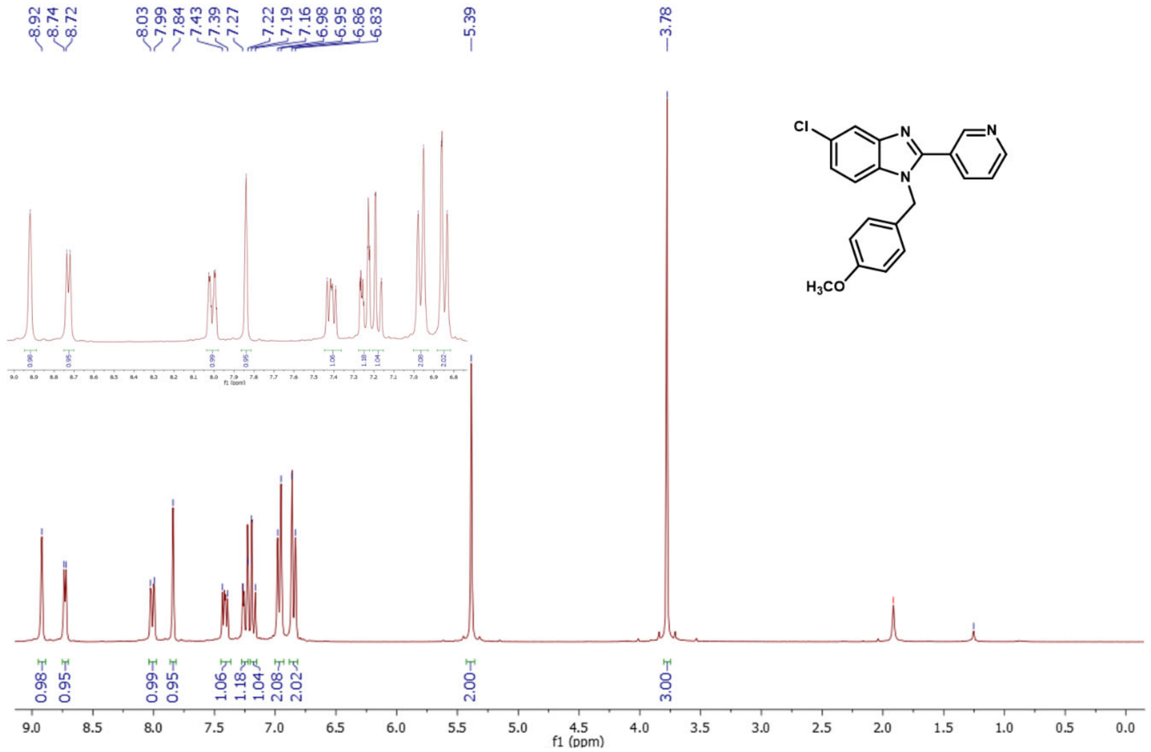

^{13}C NMR (75.5 MHz, $CDCl_3$)

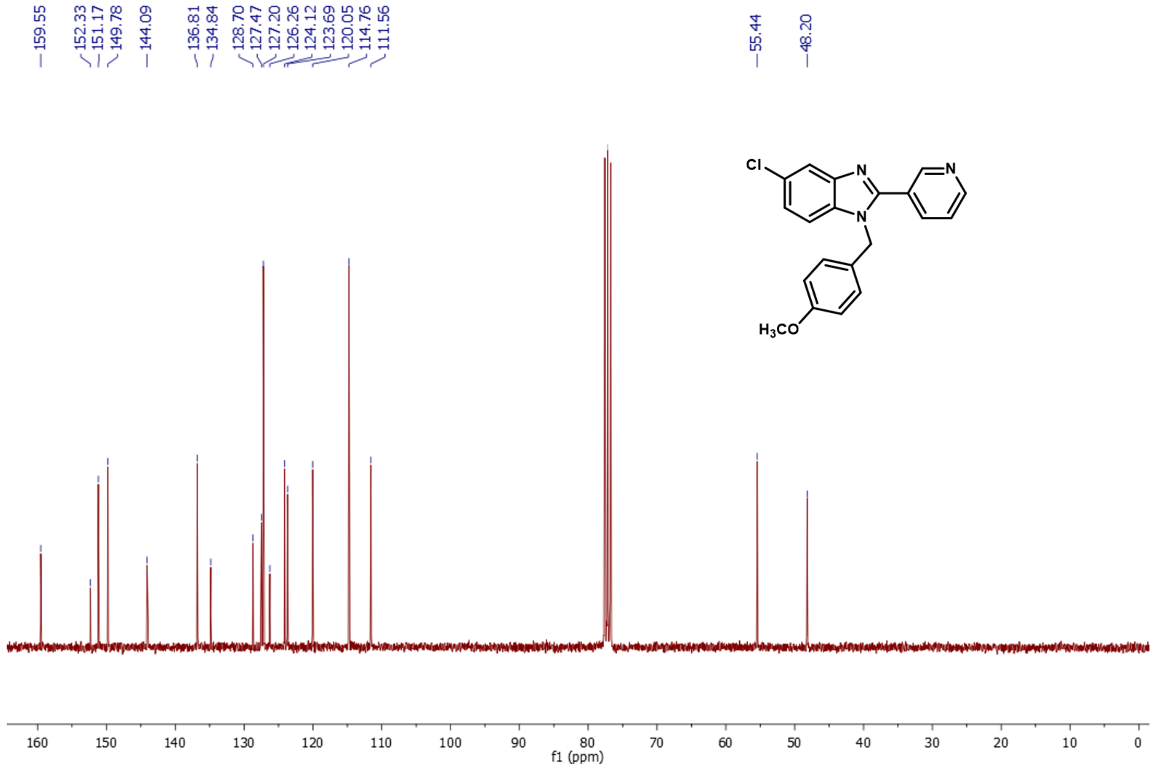

DEPT-135 – NMR

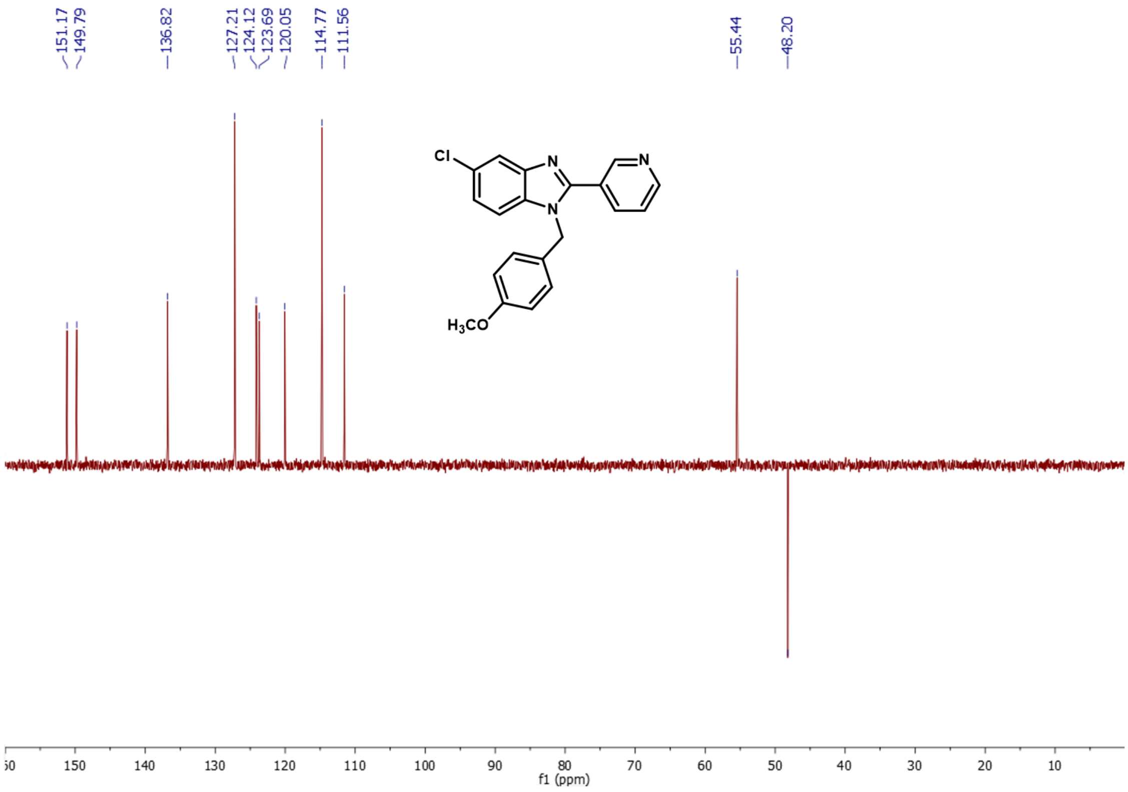

^1H–^1H – COSY NMR

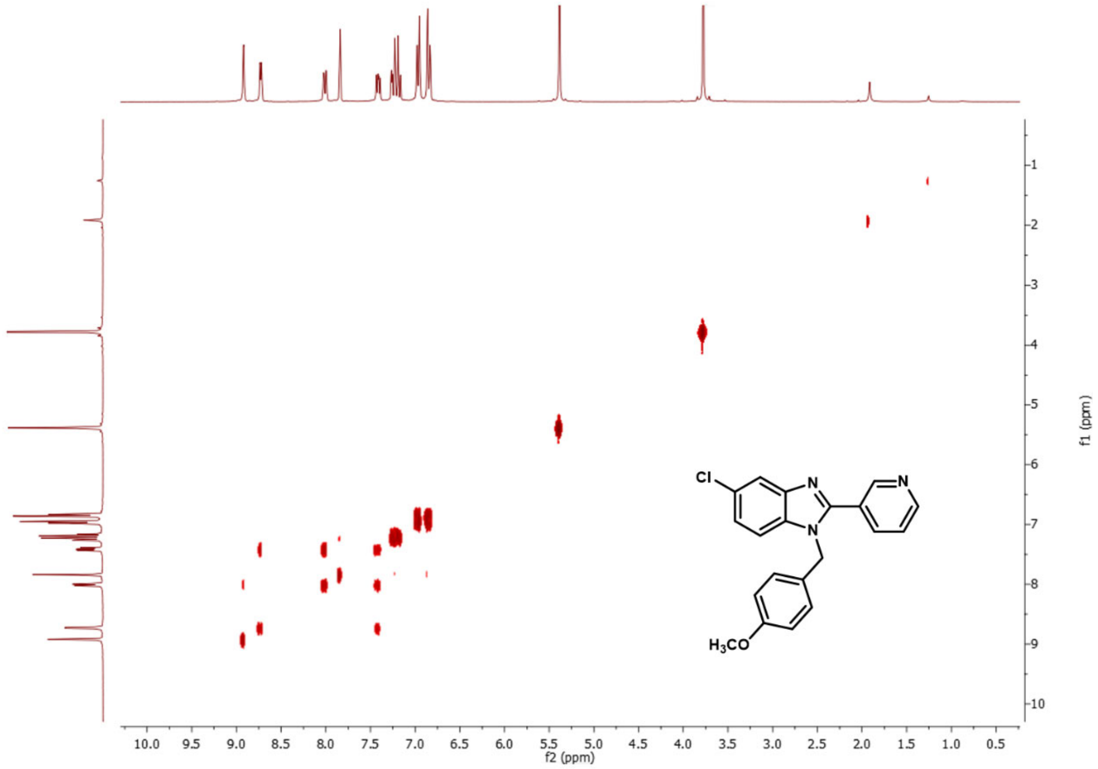

^{1}H–^{13}C – HSQC NMR

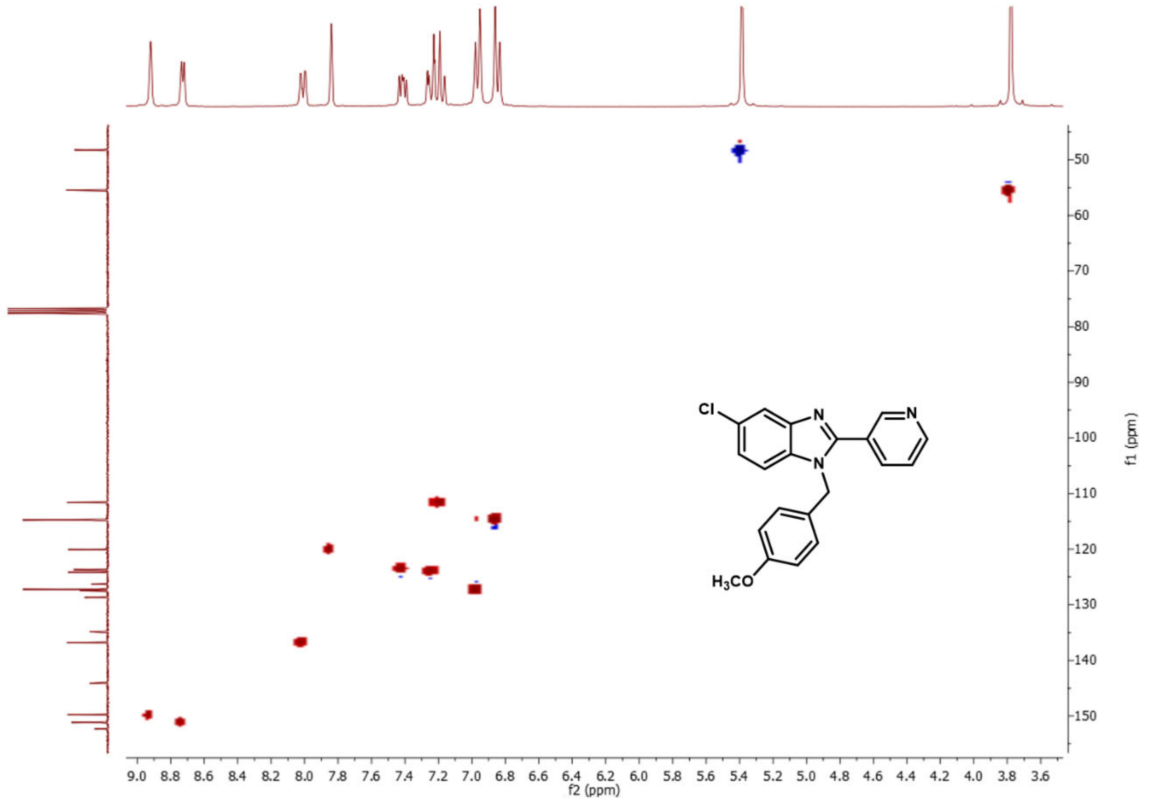

^{1}H–^{13}C – HMBC NMR

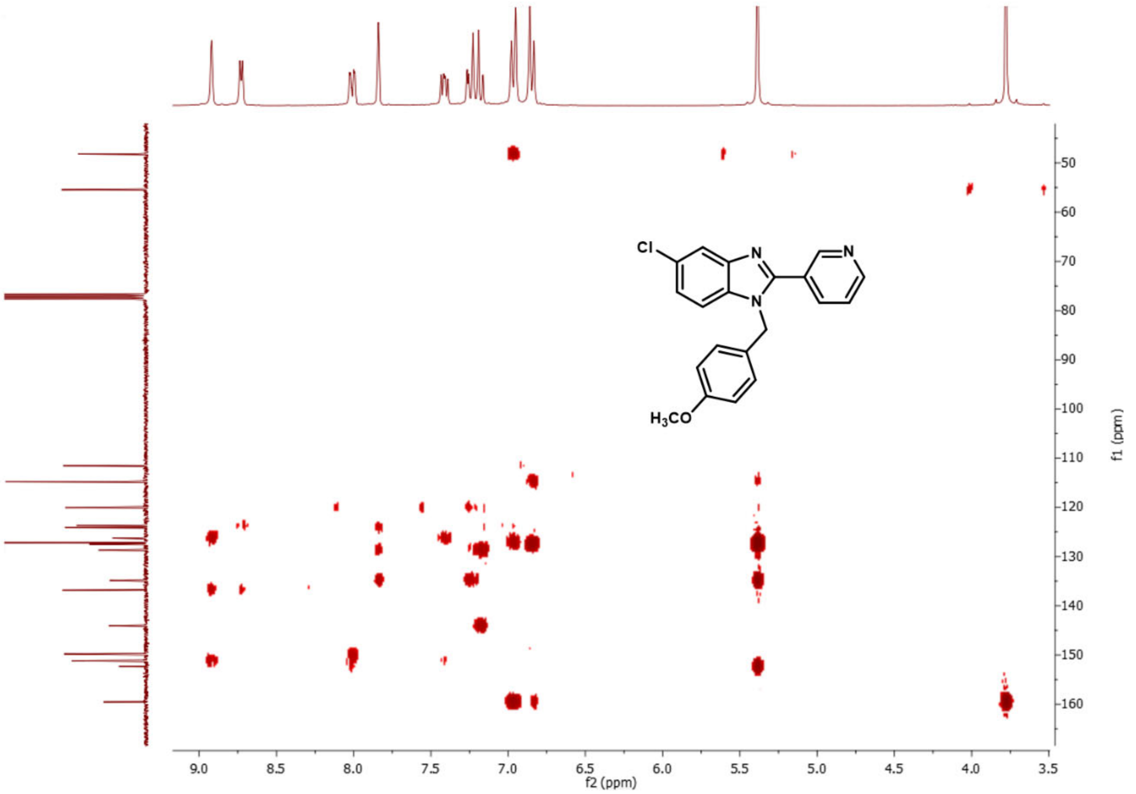

6-chloro-1-(4-methoxybenzyl)-2-(pyridin-3-yl)-1*H*-benzo[*d*]imidazole (**4b'**)

^{1}H NMR (300 MHz, $CDCl_3$)

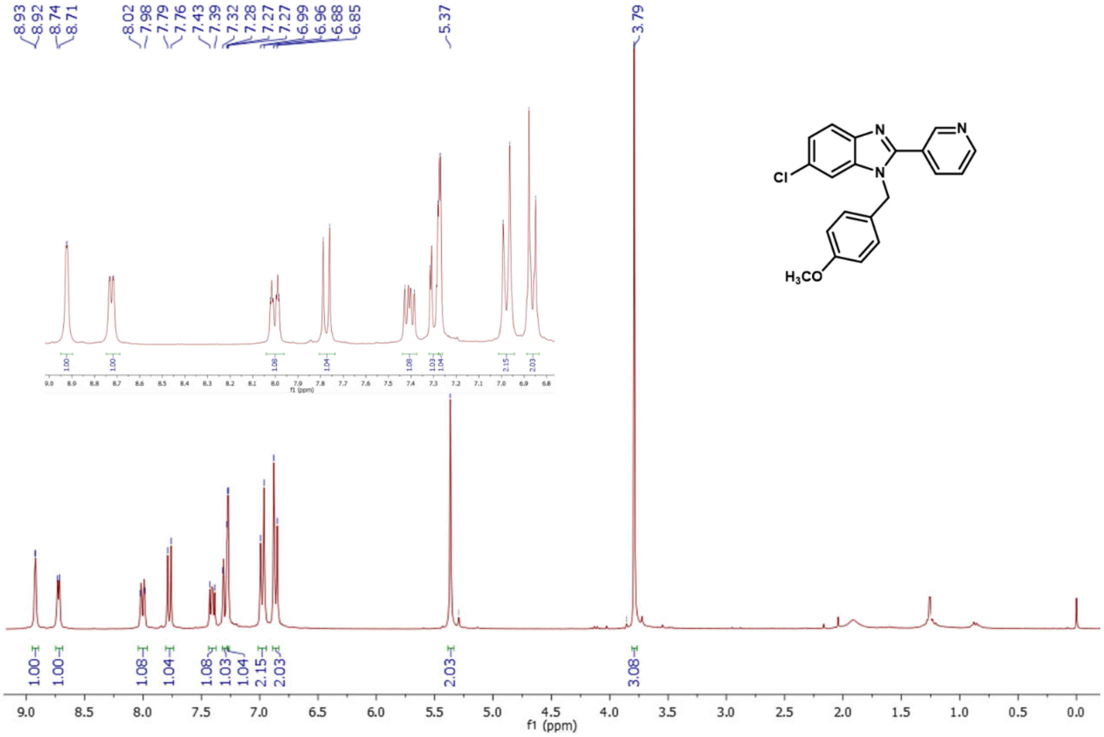

^{13}C NMR (75.5 MHz, $CDCl_3$)

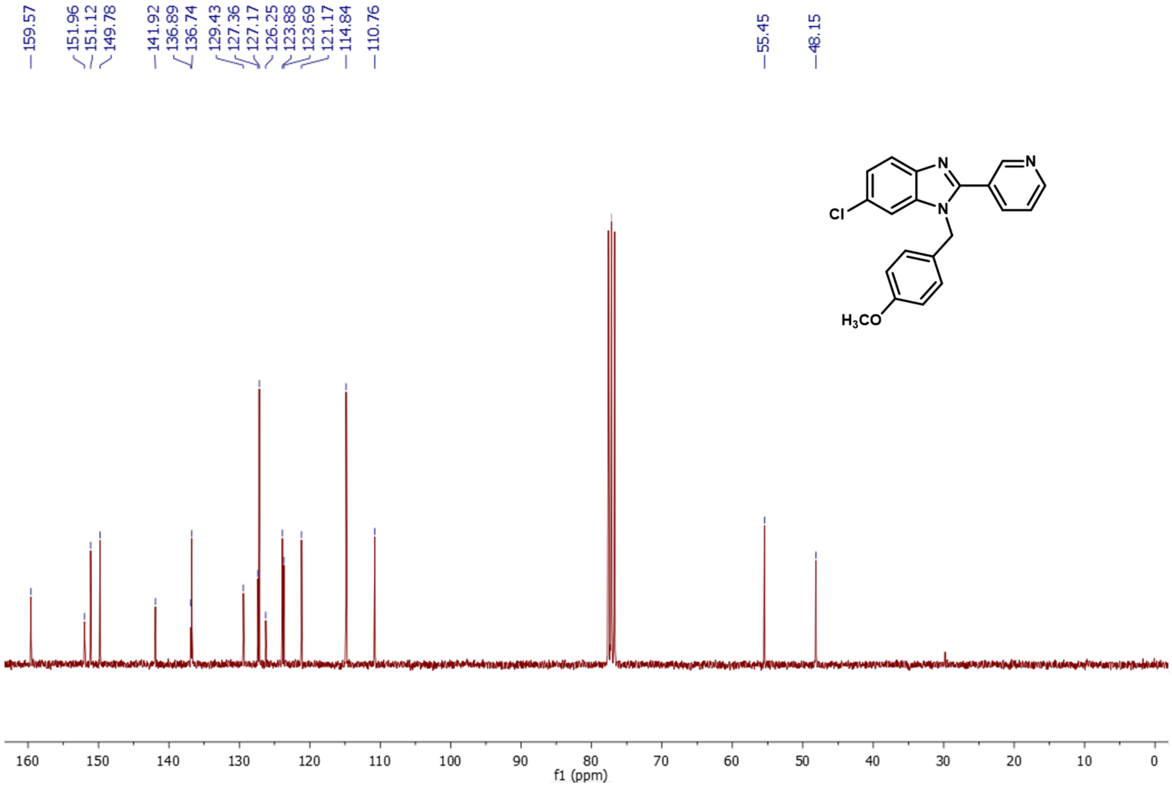

DEPT-135 – NMR

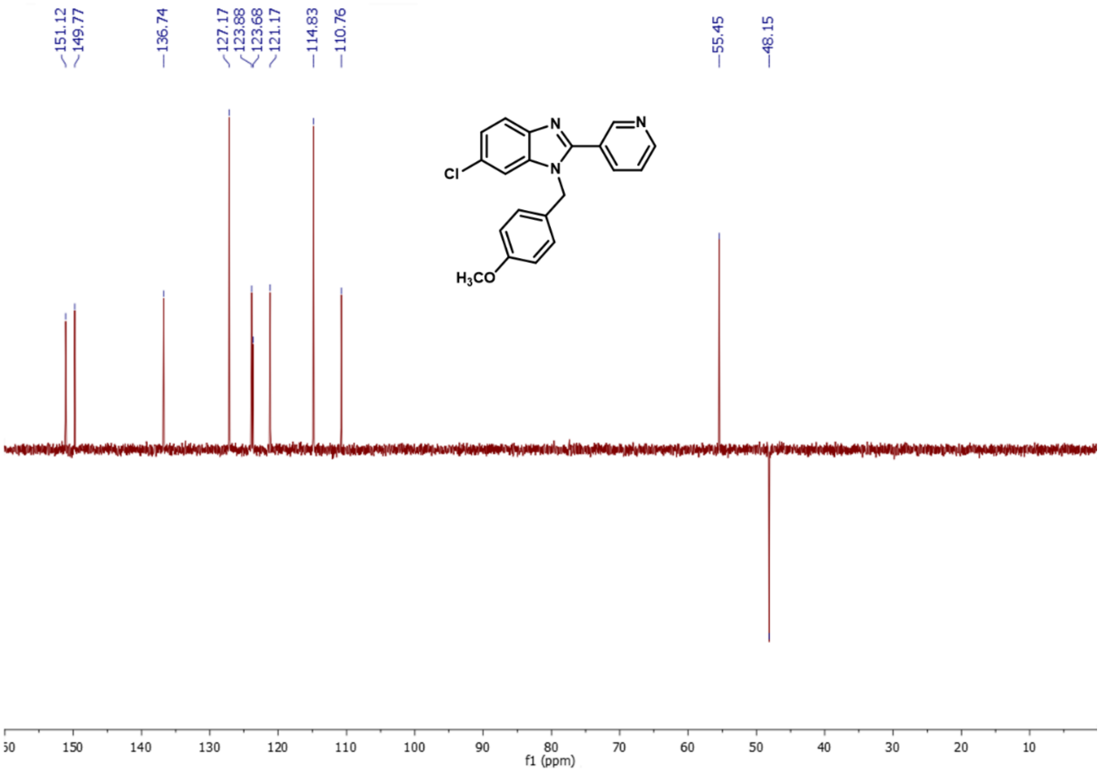

¹H–¹H – COSY NMR

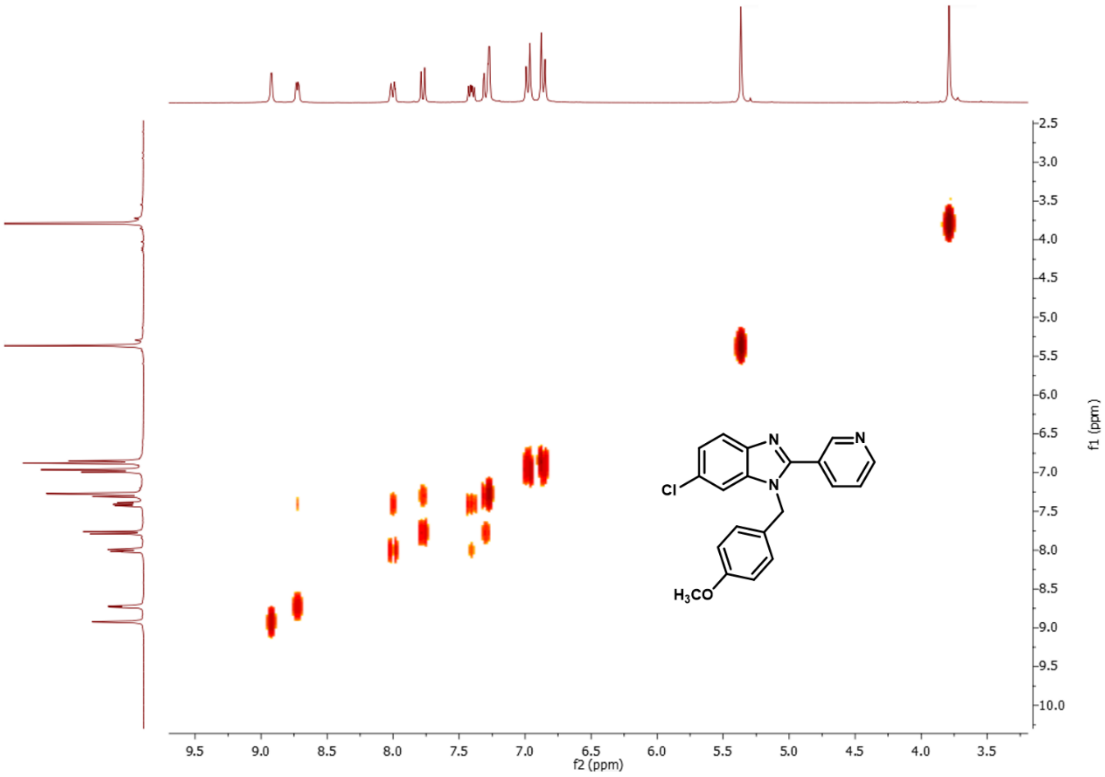

^{1}H–^{13}C – HSQC NMR

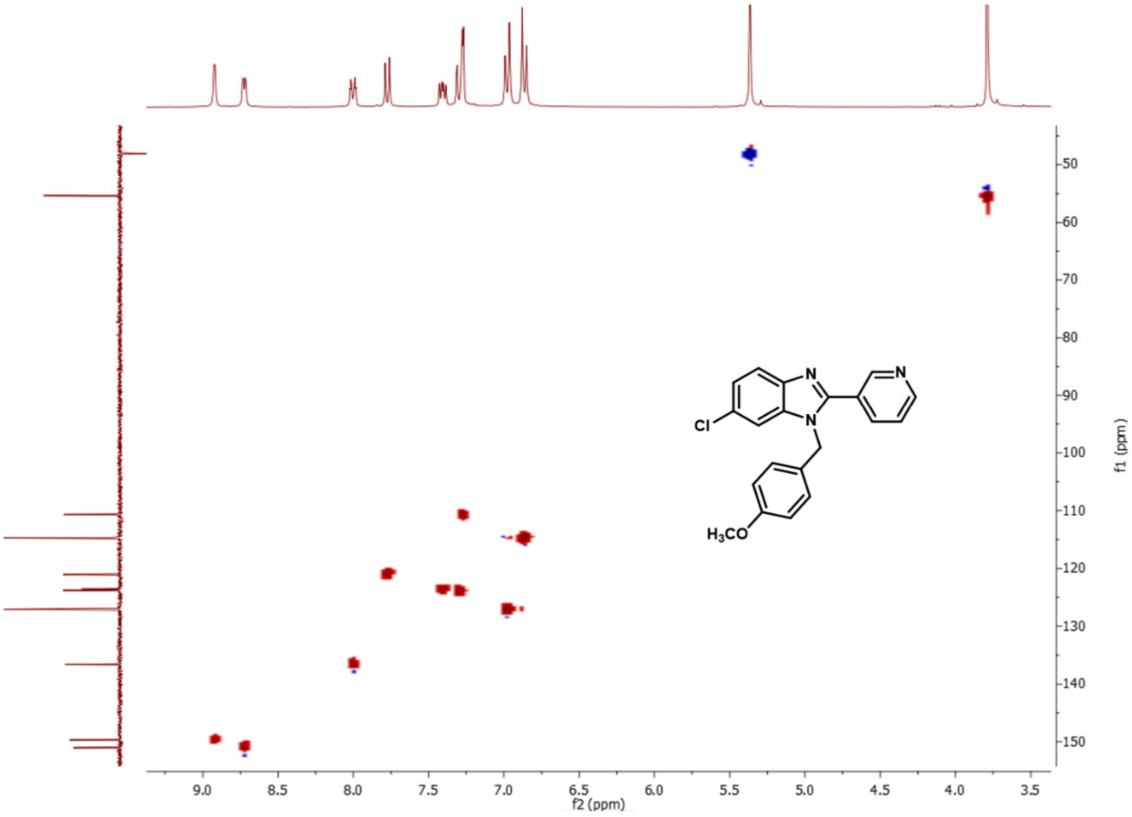

^{1}H–^{13}C – HMBC NMR

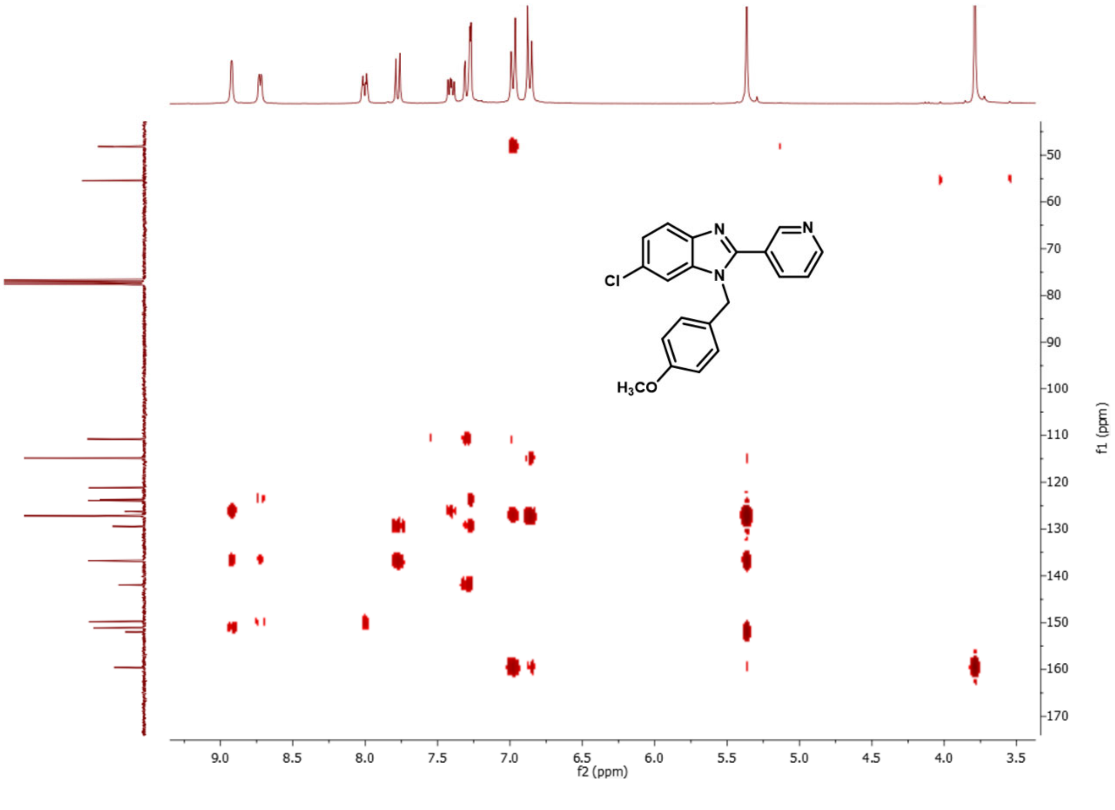

5-chloro-2-(furan-2-yl)-1-(4-methoxybenzyl)-1*H*-benzo[*d*]imidazole (**4c**)

^{1}H NMR (300 MHz, $CDCl_3$)

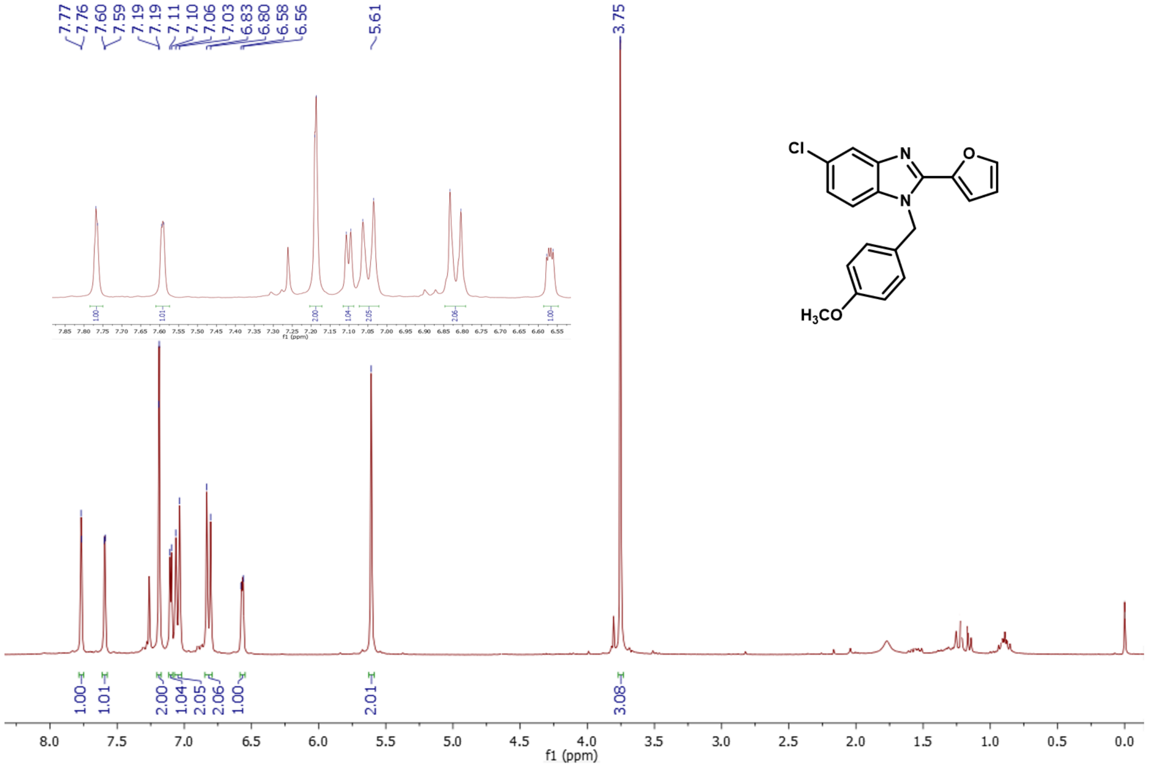

^{13}C NMR (75.5 MHz, $CDCl_3$)

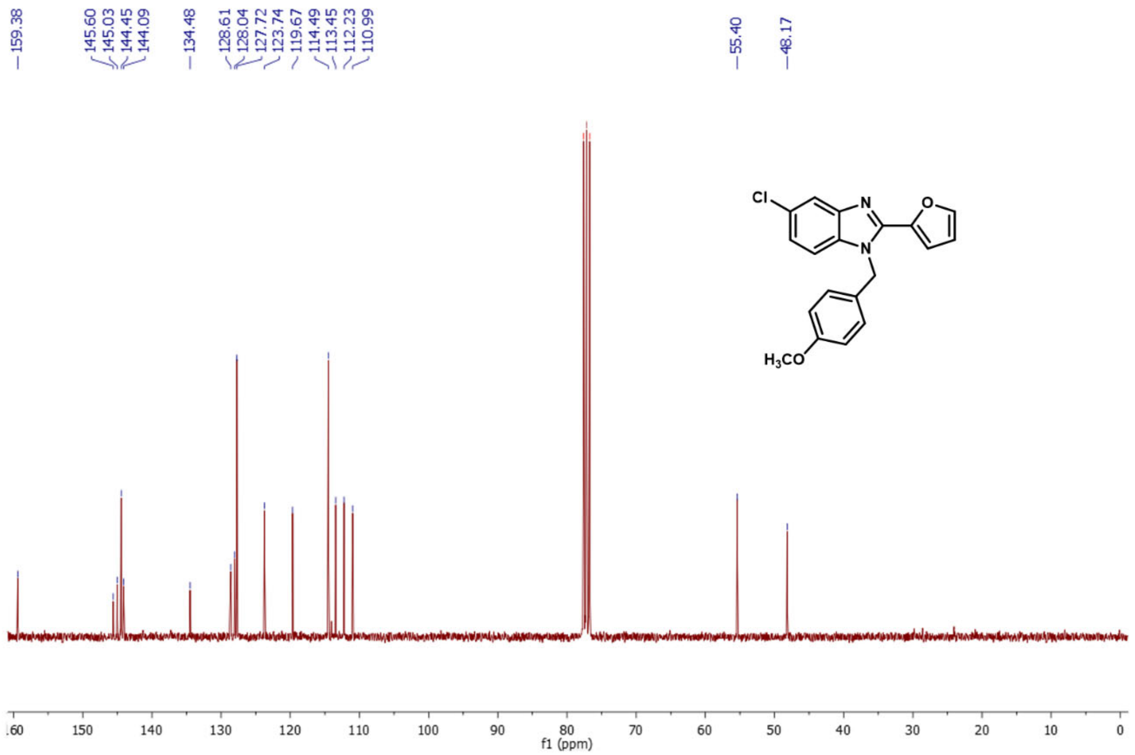

DEPT-135 – NMR

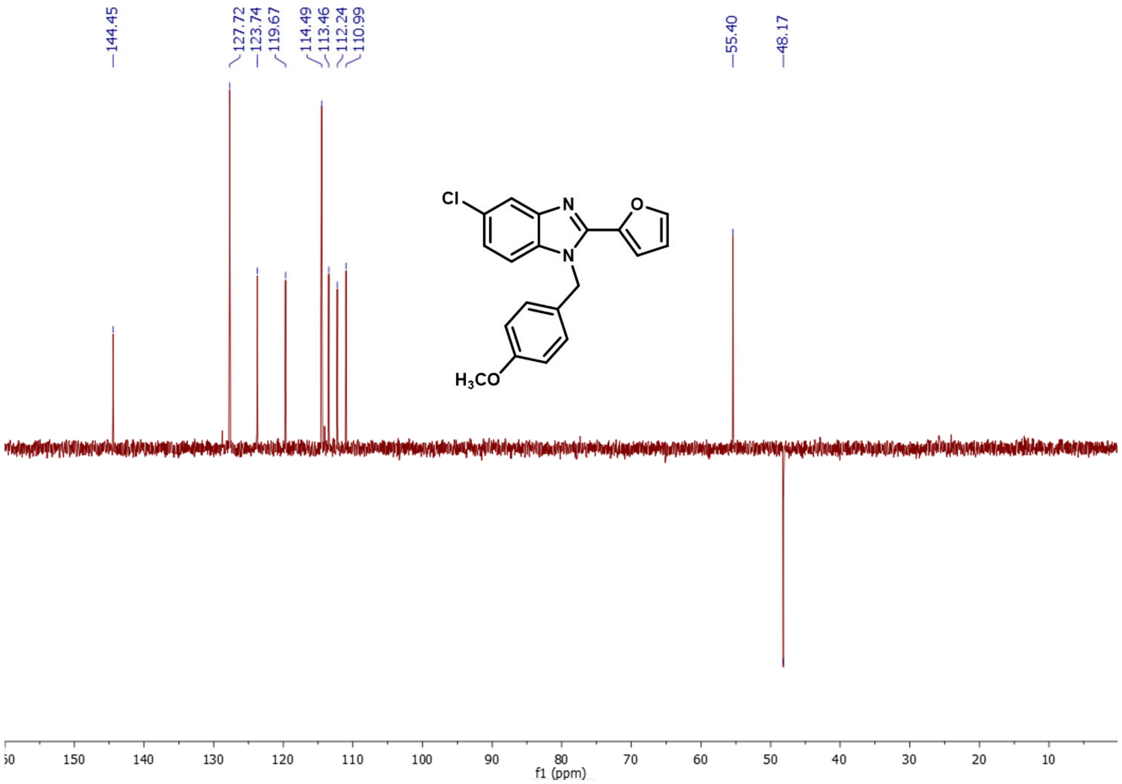

^{1}H–^{1}H – COSY NMR

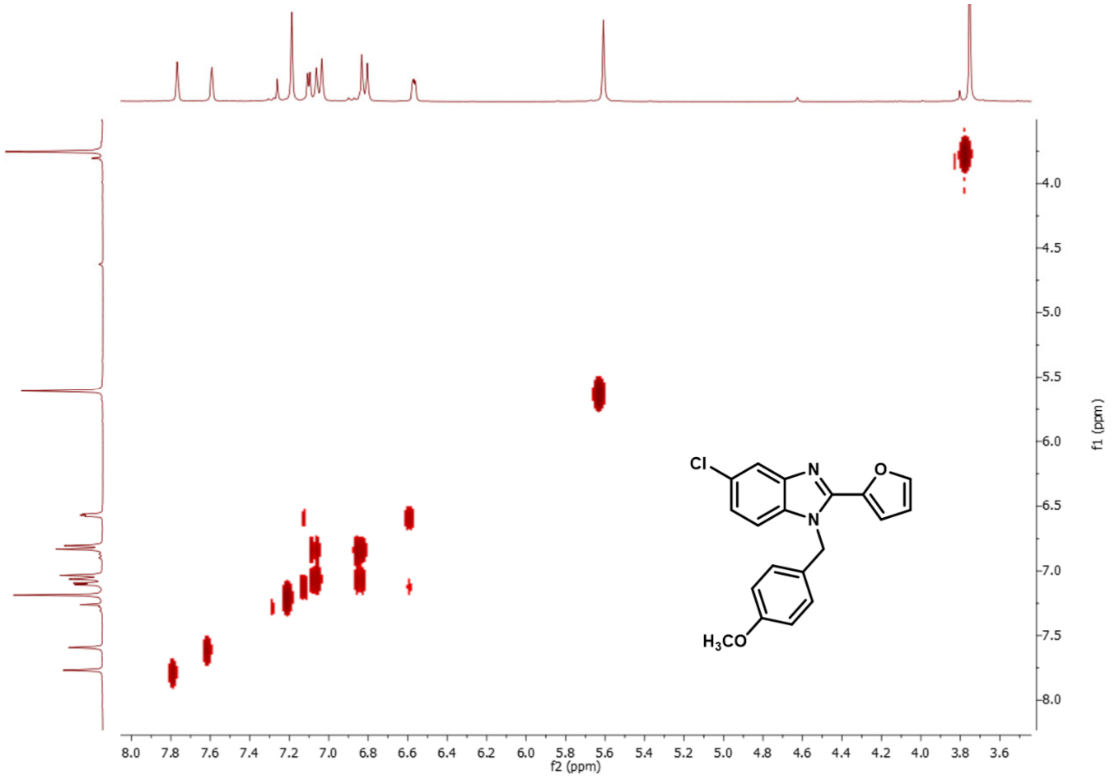

$^{1}H–^{13}C$ – HSQC NMR

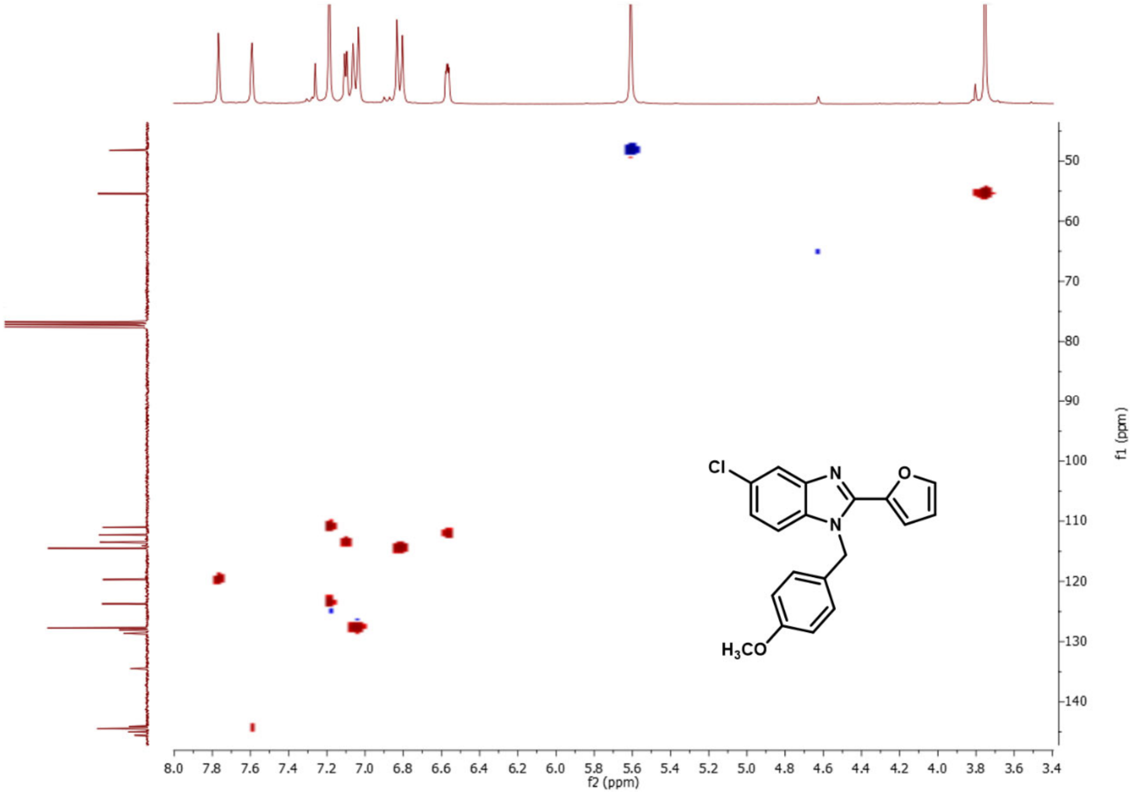

$^{1}H–^{13}C$ – HMBC NMR

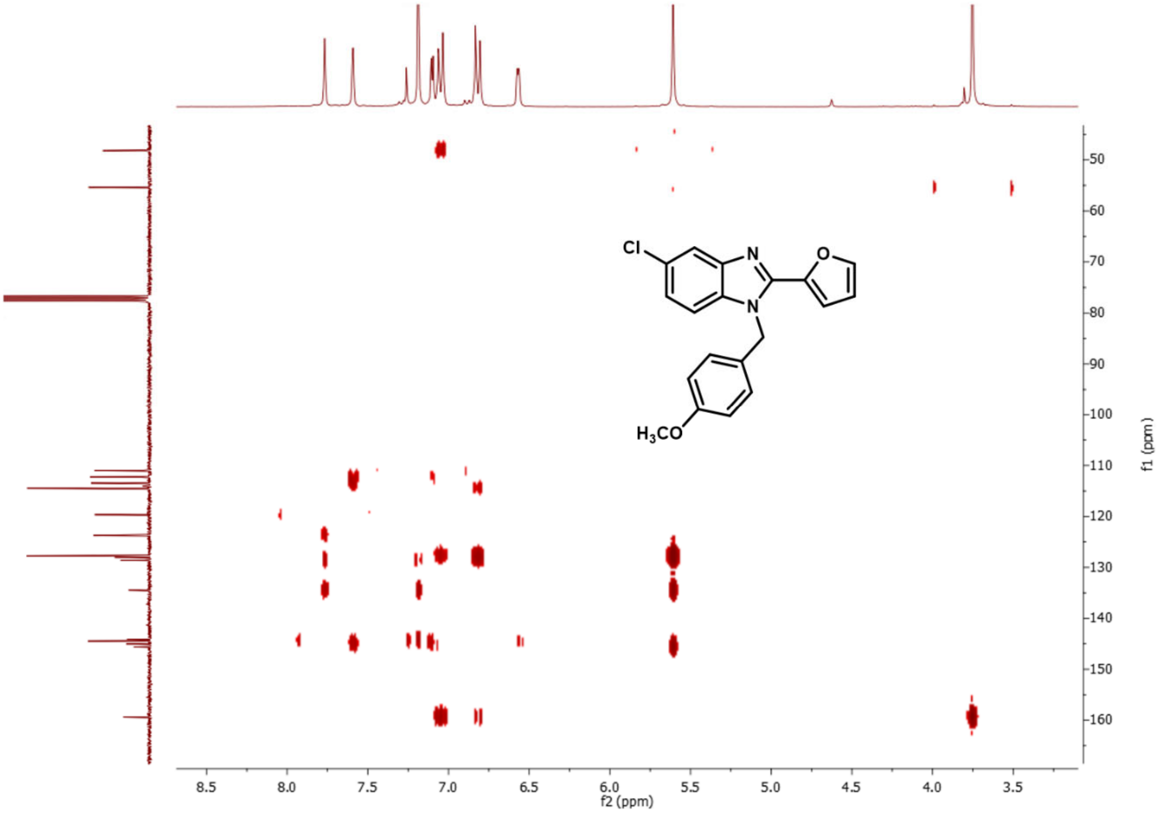

6-chloro-2-(furan-2-yl)-1-(4-methoxybenzyl)-1*H*-benzo[*d*]imidazole (**4c’**)

^{1}H NMR (300 MHz, $CDCl_3$)

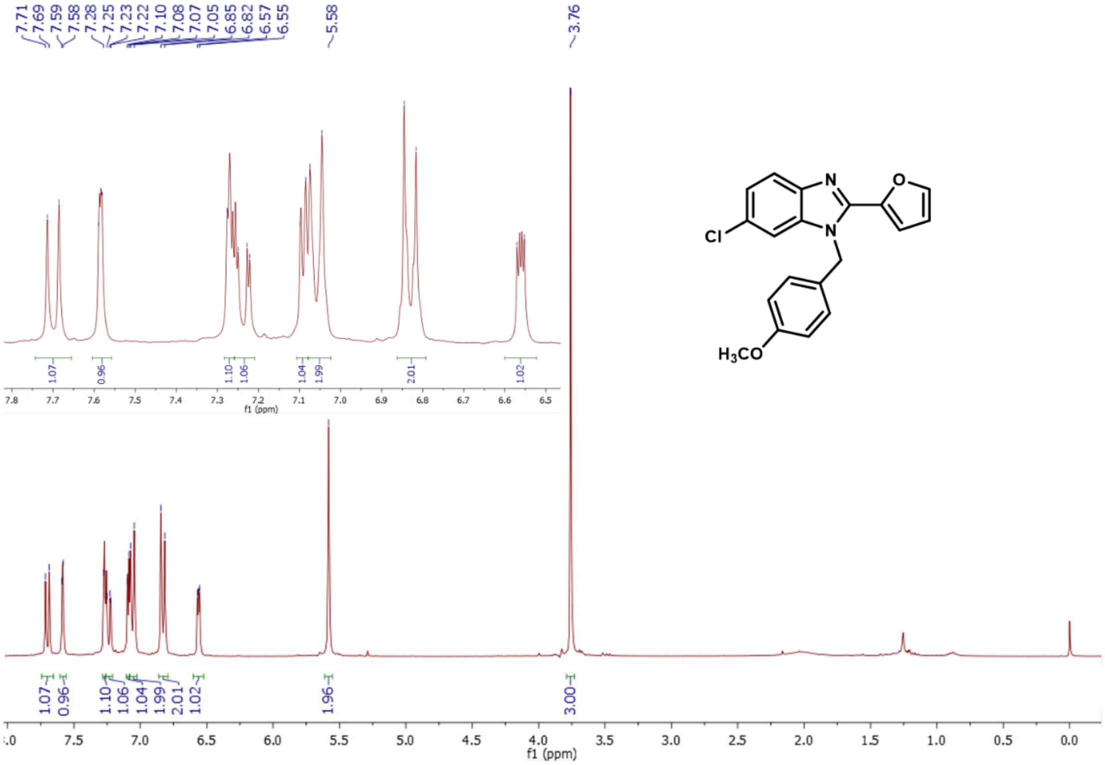

^{13}C NMR (75.5 MHz, $CDCl_3$)

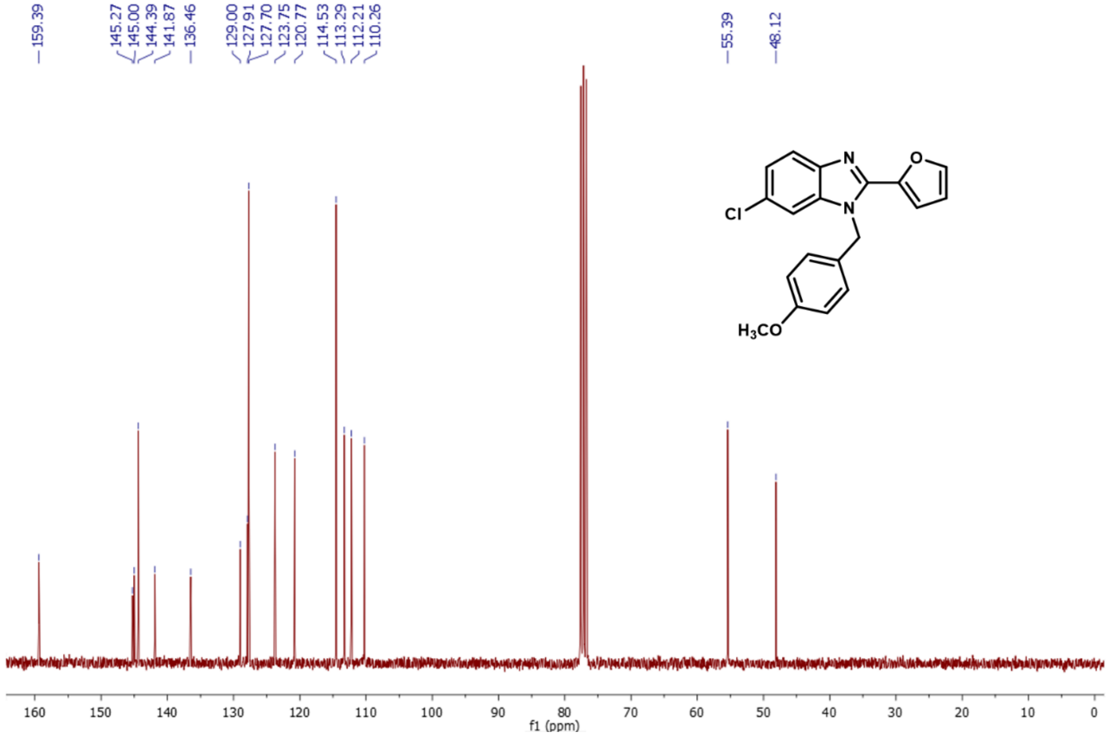

DEPT-135 – NMR

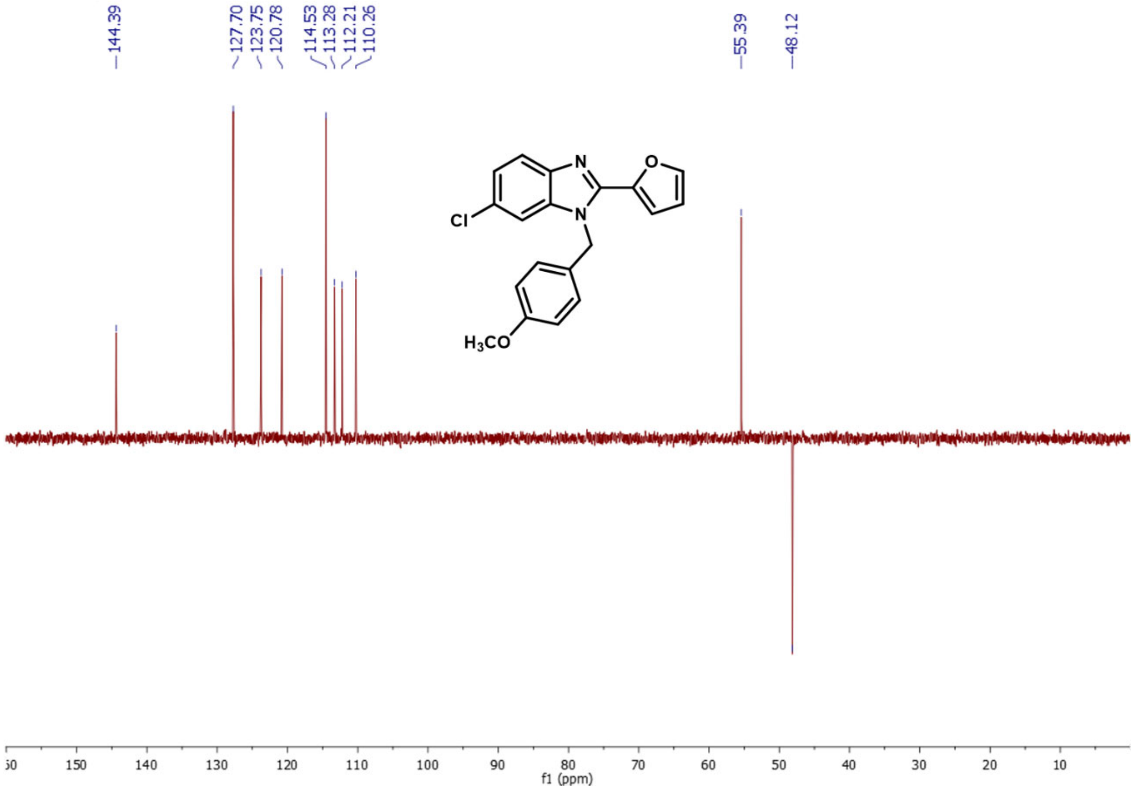

^{1}H–^{1}H – COSY NMR

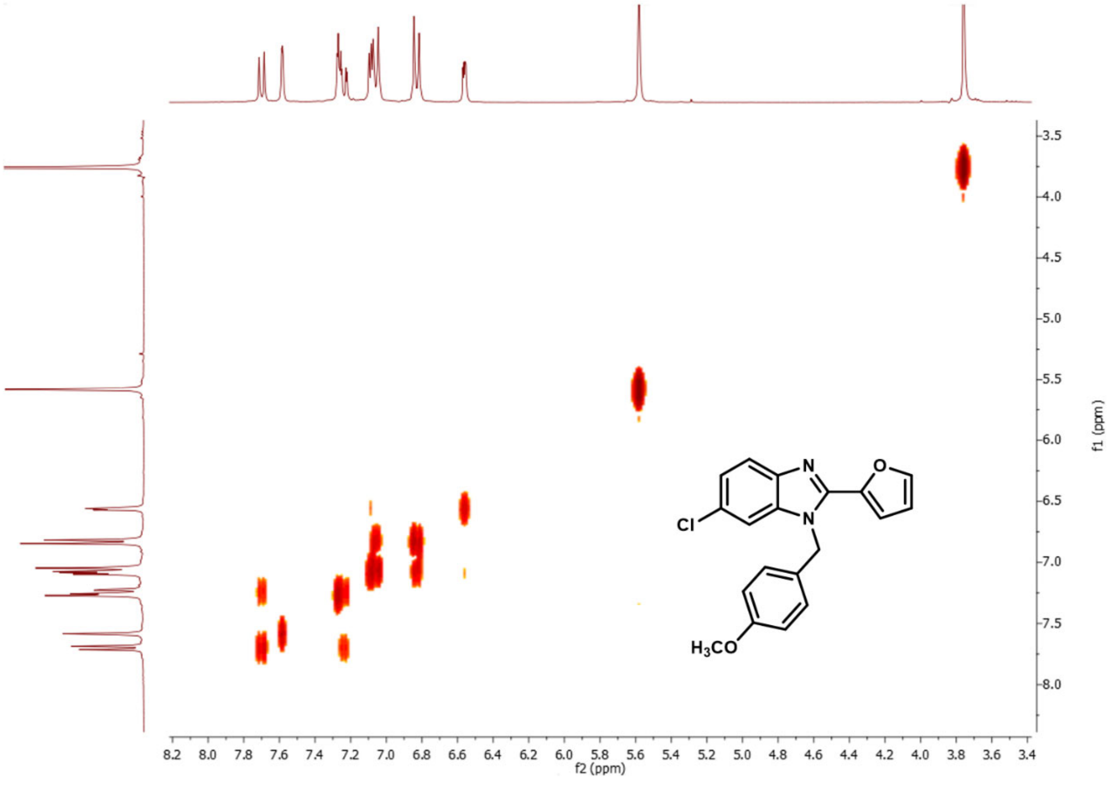

^{1}H–^{13}C – HSQC NMR

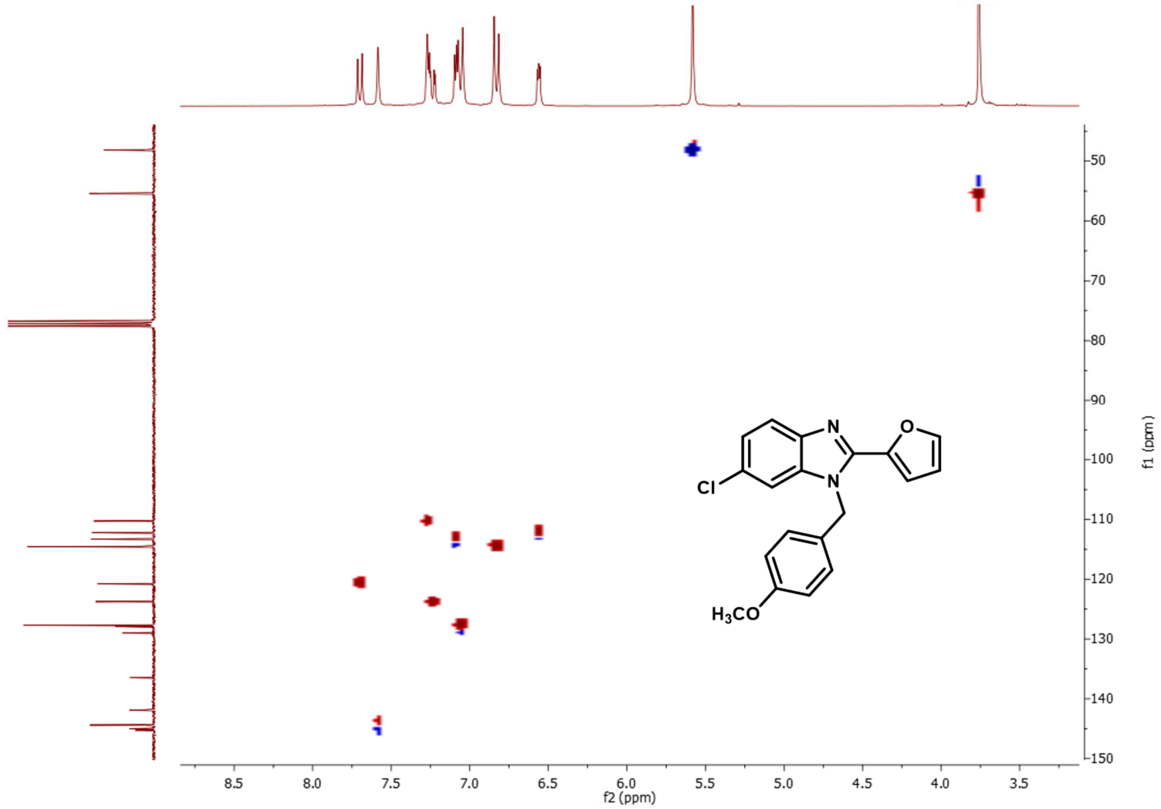

^{1}H–^{13}C – HMBC NMR

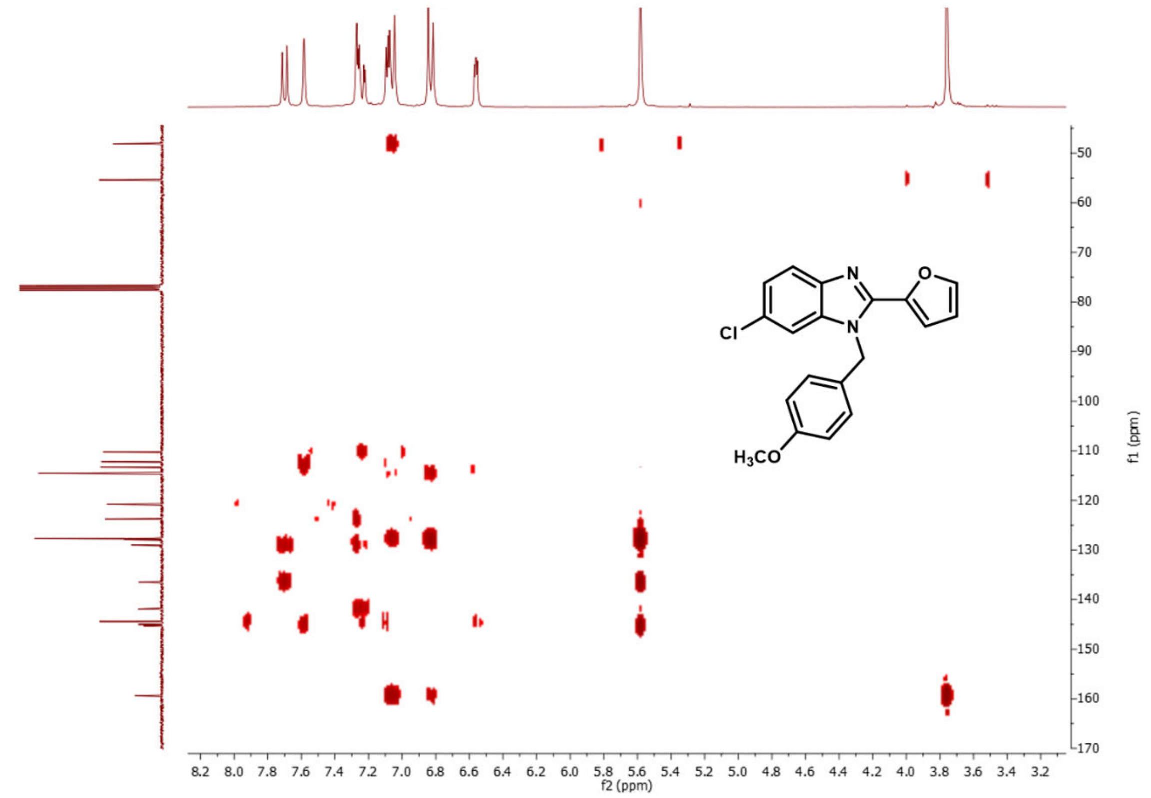

3-(5-chloro-1-(4-methoxybenzyl)-1*H*-benzo[*d*]imidazol-2-yl)isoxazole (**4d**)

^{1}H NMR (300 MHz, DMSO-d^{6})

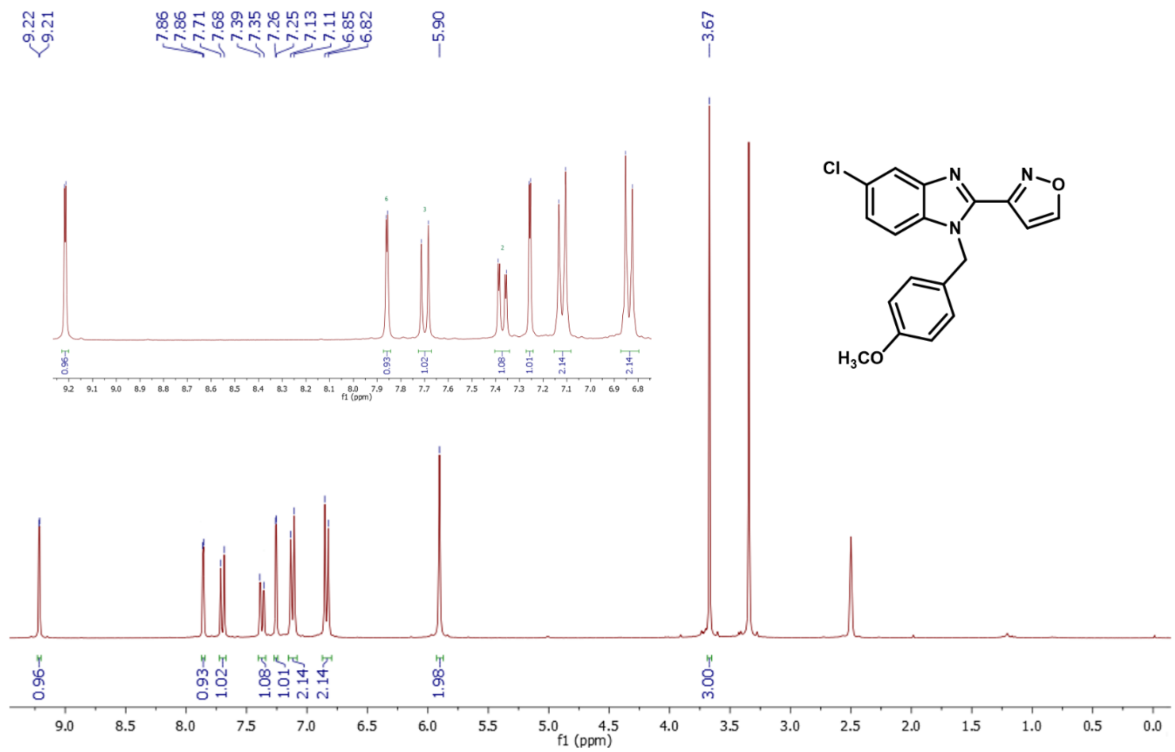

^{13}C NMR (75.5 MHz, DMSO-d^{6})

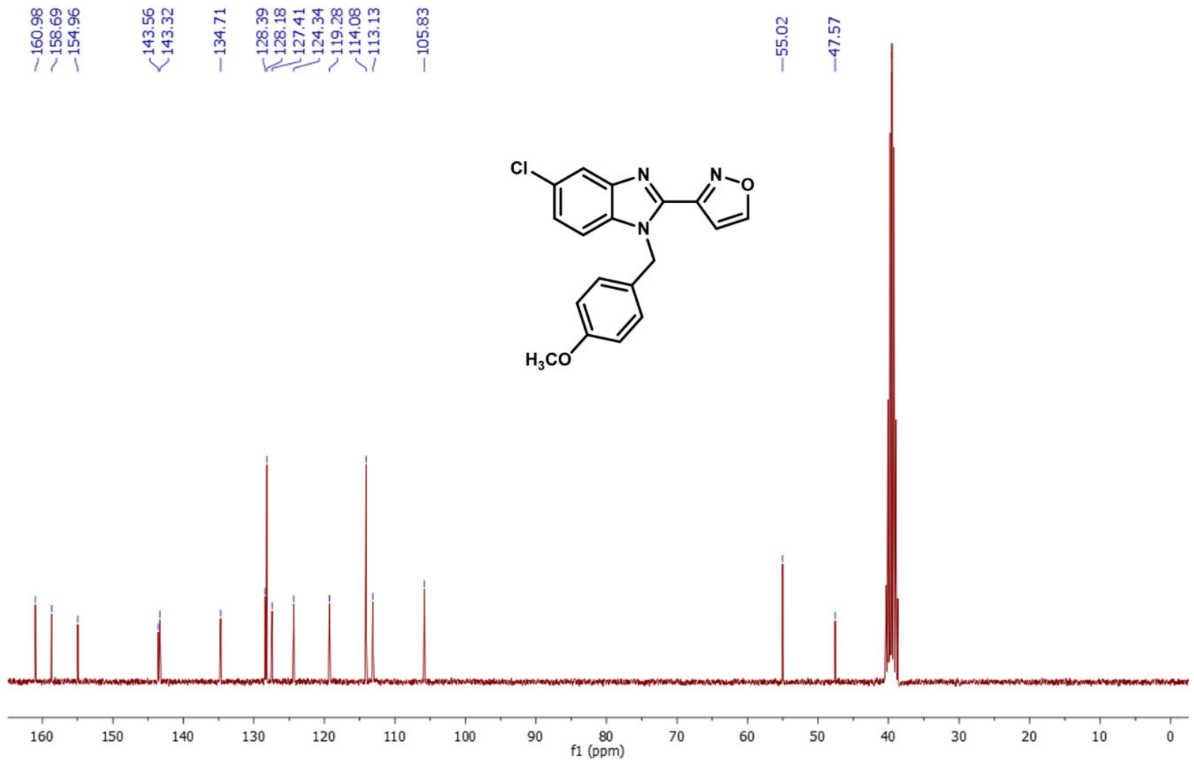

DEPT-135 – NMR

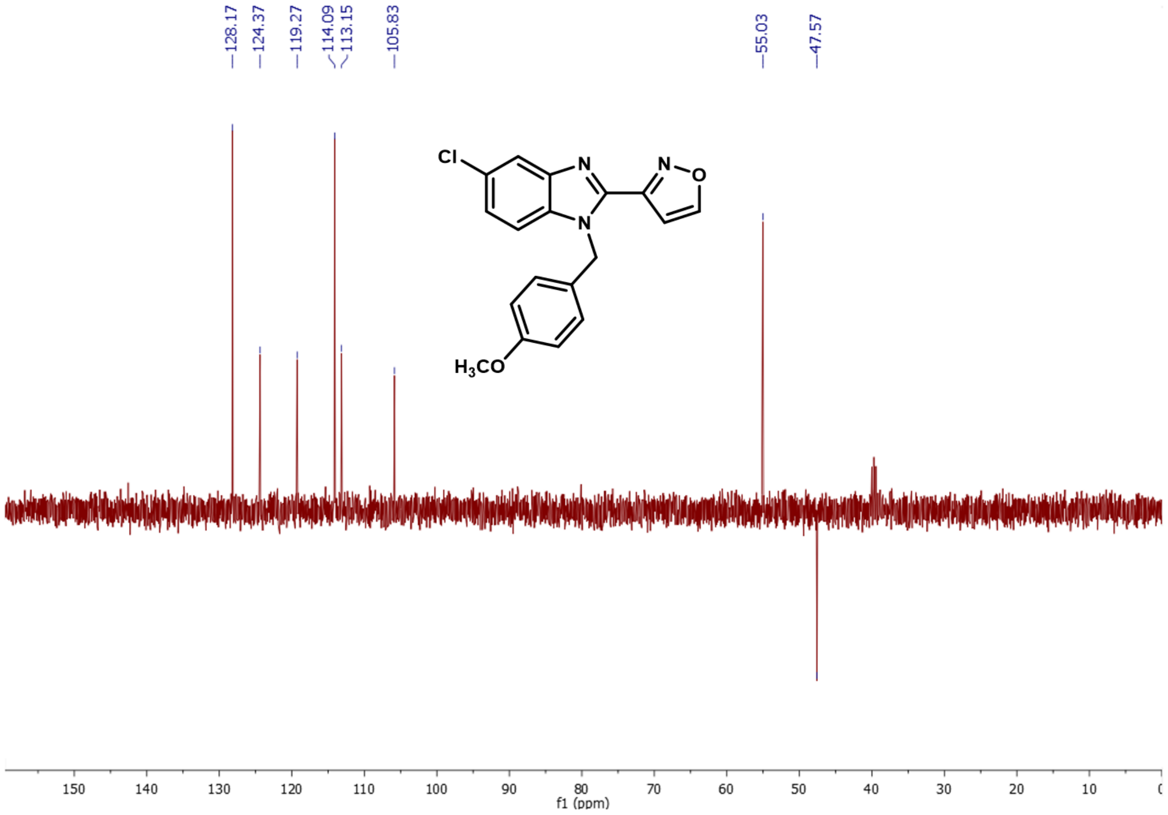

^{1}H–^{1}H – COSY NMR

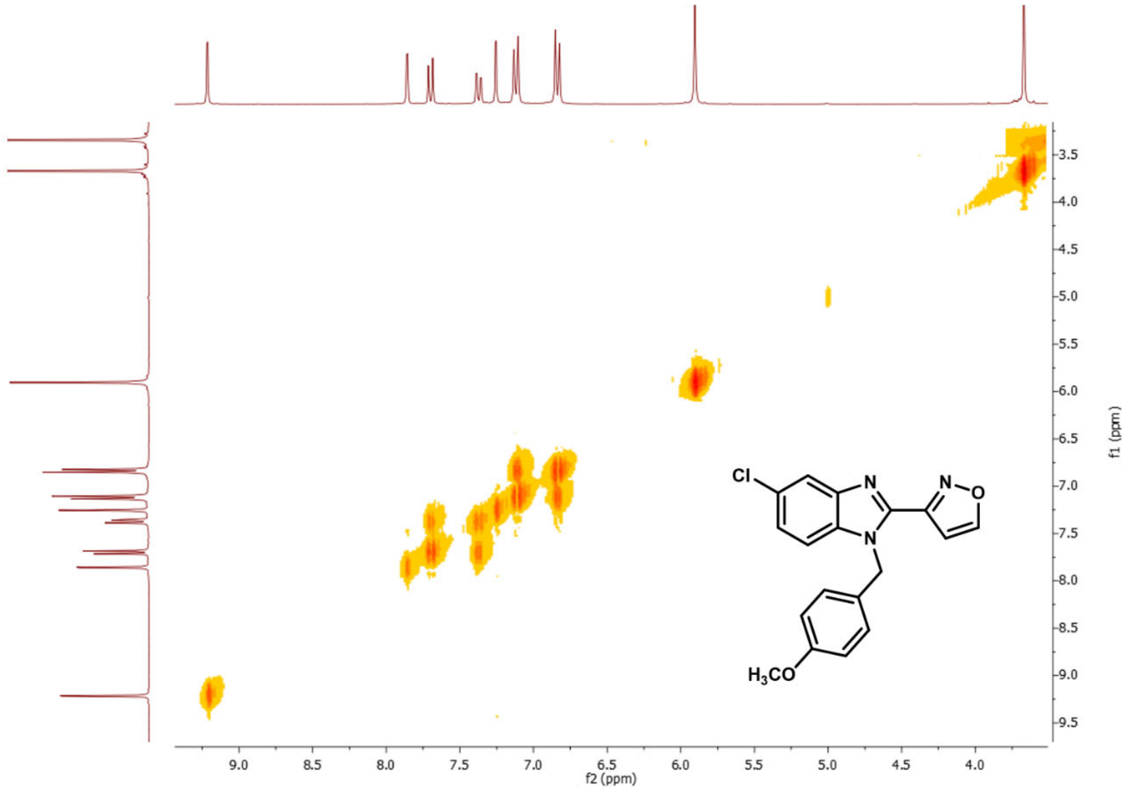

$^{1}H–^{13}C$ – HSQC NMR

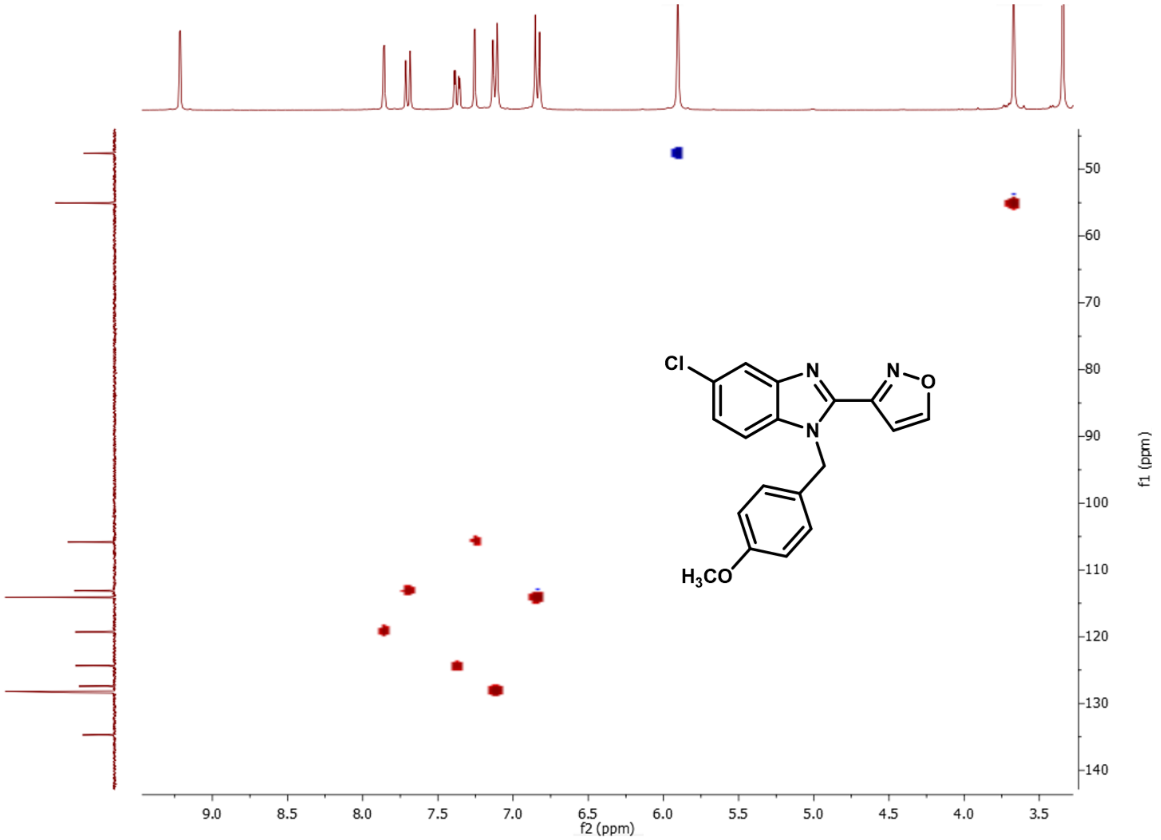

$^{1}H–^{13}C$ – HMBC NMR

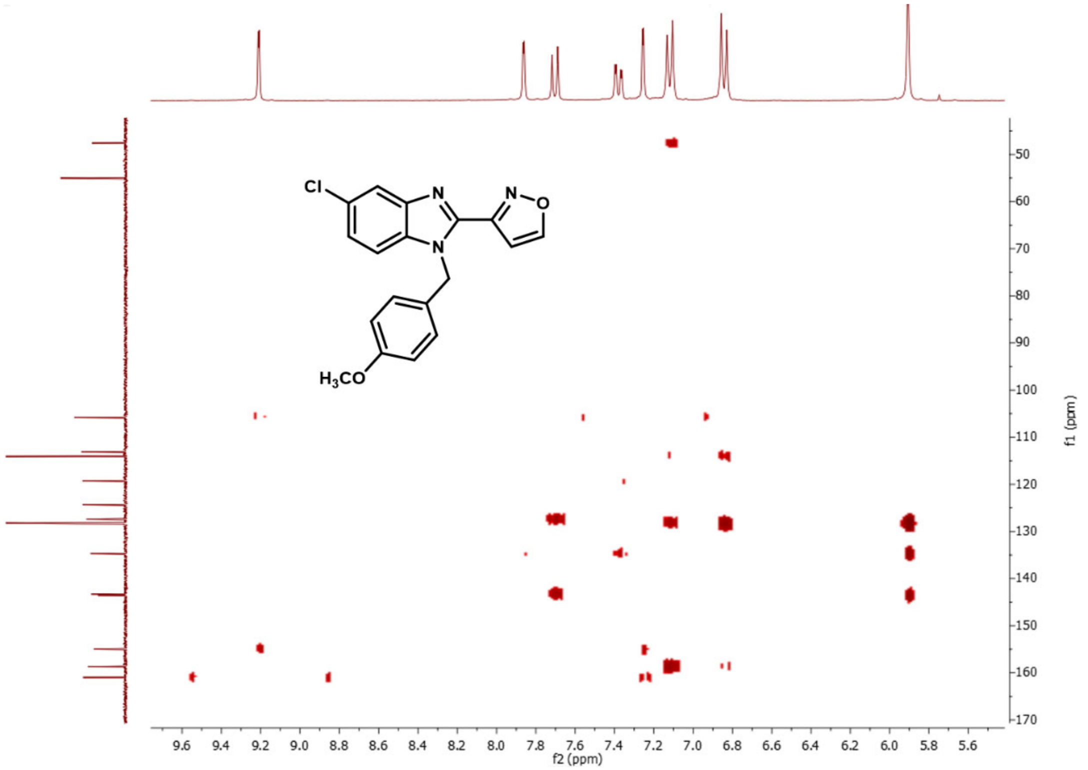

3-(6-chloro-1-(4-methoxybenzyl)-1*H*-benzo[*d*]imidazol-2-yl)isoxazole (**4d'**)

^{1}H NMR (300 MHz, DMSO-d^{6})

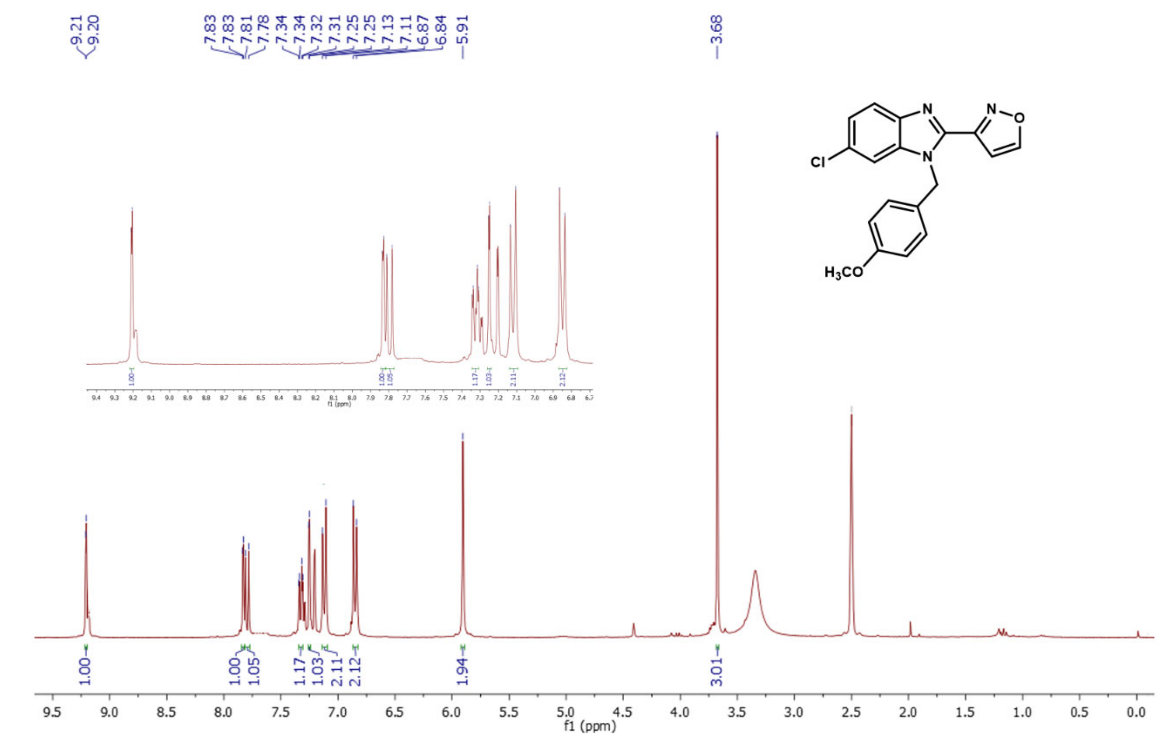

^{13}C NMR (75.5 MHz, DMSO-d^{6})

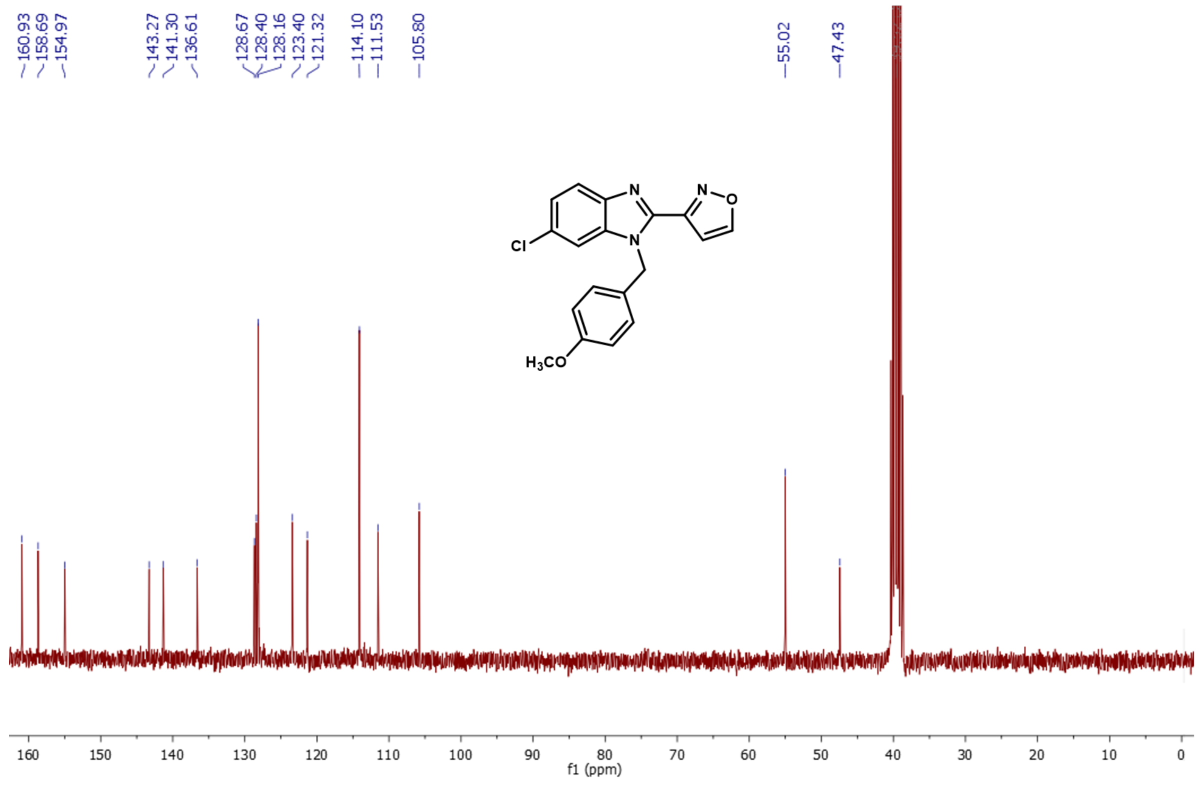

DEPT-135 – NMR

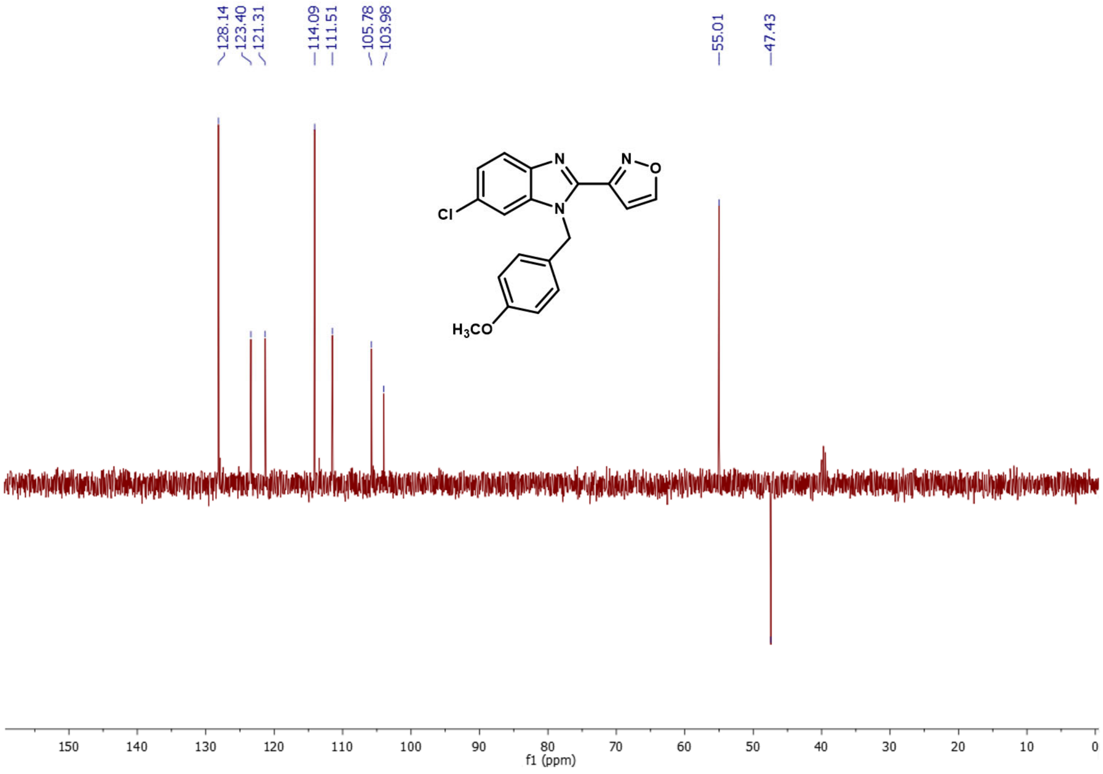

^1H–^1H – COSY NMR

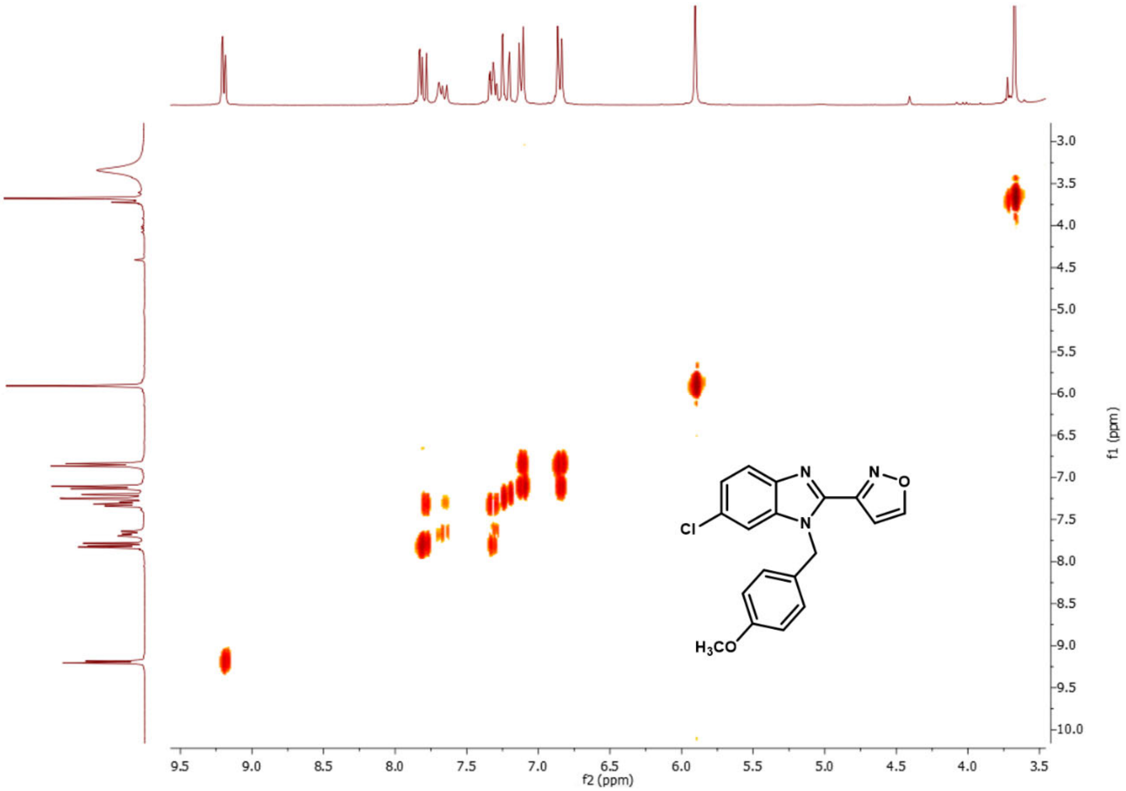

^{1}H–^{13}C – HSQC NMR

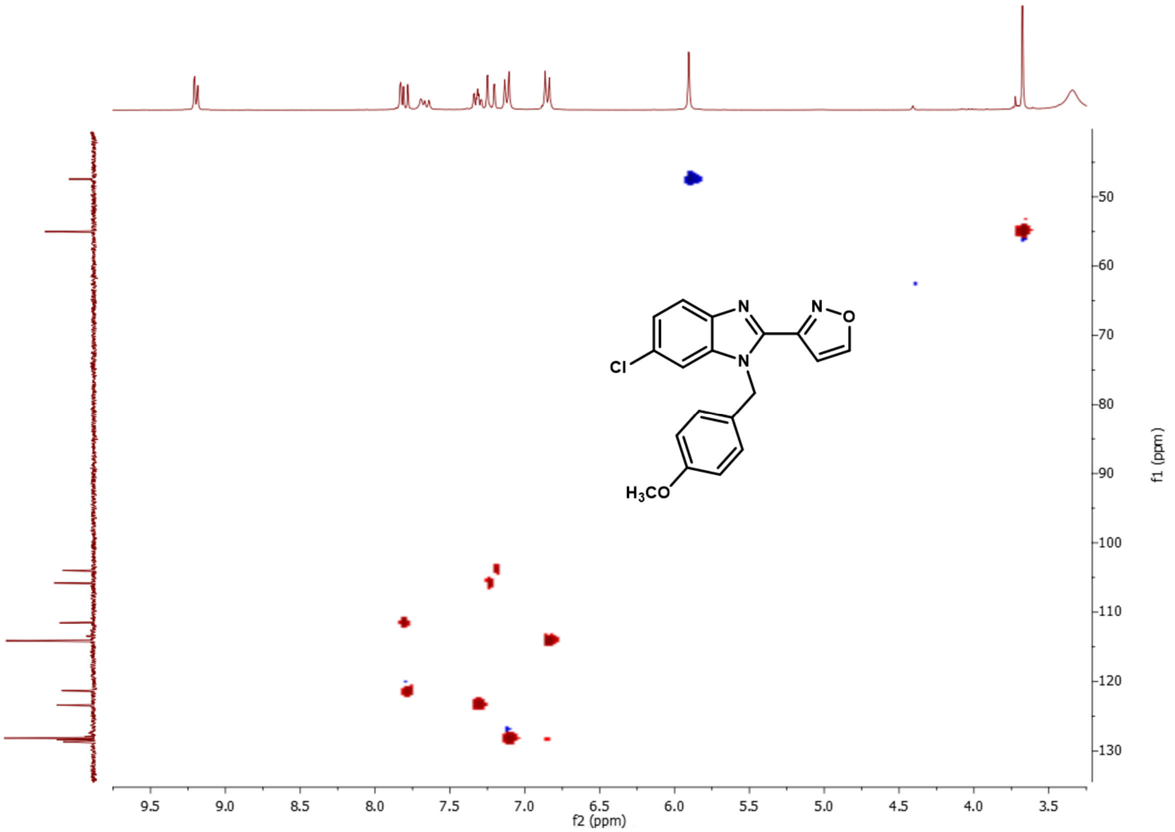

^{1}H–^{13}C – HMBC NMR

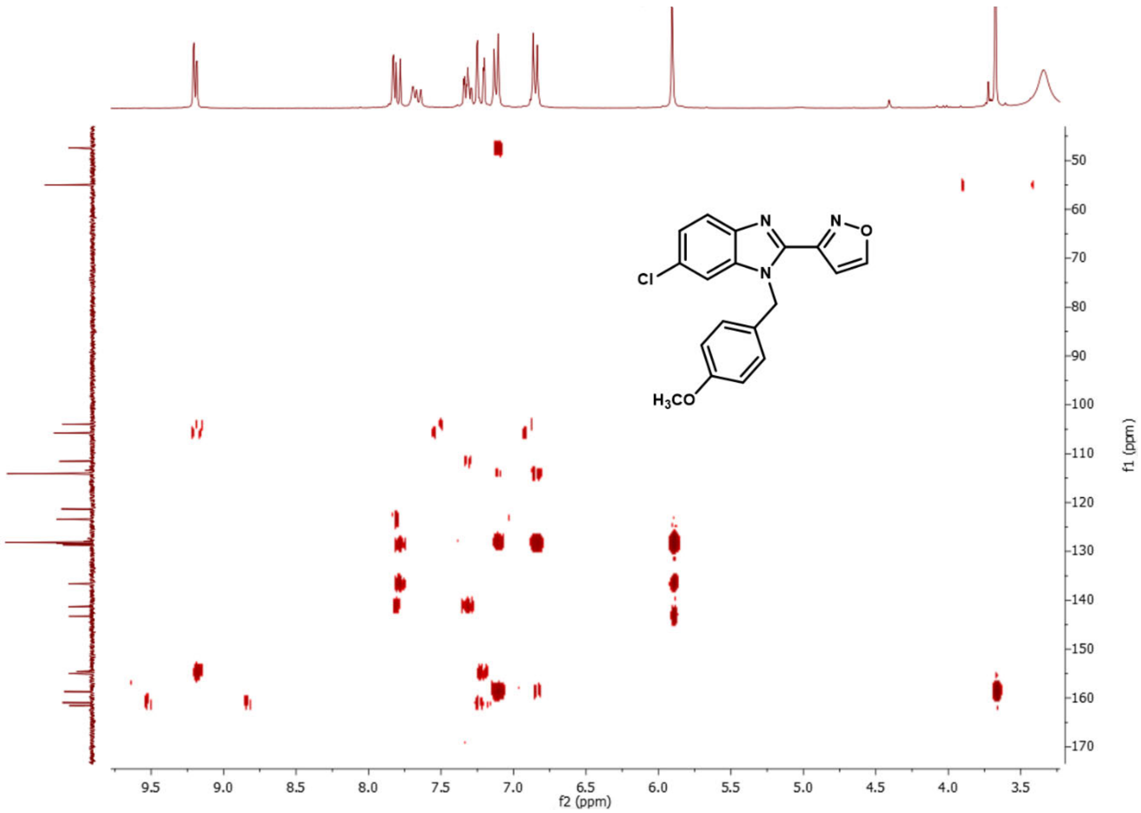

Biological experiments

Cell cultures.

Human embryonic kidney 293 (HEK293), neoplastic glioblastoma cells (U87MG), and acute promyelocytic leukemia cells (HL-60) were maintained at 5% CO_2 and 37 °C in DMEM, EMEM and RPMI medium, respectively containing 10% Fetal Bovine Serum and 100 U/mL penicillin and 100 mg/mL streptomycin.

Cell viability: ([MTT - (3-(4,5-dimethylthiazol-2-yl)-2,5-diphenyltetrazolium bromide) - formazan]

HEK293, U87MG, and HL-60 cells were first quantified to determine the exact number to be used in the MTT cell viability assays, ensuring that absorbance values remained below 1 unit in accordance with Beer-Lambert(1).

HEK293, U87MG and HL-60 cell lines were seed in a flat-bottom 96-wells plate at a density of 10,000 cells per well. Cells were then incubated with WIN-55,212-2 and AM630 at various concentrations (0.001 µM to 10 µM or 20 µM in case of HL-60 cells) in 200 µL of respective culture medium supplemented with 10% fetal bovine serum (FBS), at 37 °C for 72 hours. Subsequently, 20 µL of 5 mg/mL MTT solution was added to each well, and cells were incubated at 37 °C for 4 h. The resulting formazan crystals were then solubilized with 10% sodium dodecyl sulfate (SDS) in 0.1 mM HCl, followed by overnight incubation at 37 °C. Untreated cells were used as the control, 0.1% of dimethyl sulfoxide (DMSO) as the vehicle control, and 0.2 % of Triton X-100 as a positive control for cell death. IC_{50} toxicity value of WIN-55,212-2 in HL-60 cell line were determined.

Competition assay between WIN-55,212-2 and AM630 in HL-60 cells was conducted under the same conditions described in the previous paragraph. Therefore, WIN-55,212-2 was assayed in the presence of increasing concentrations of the CB_2 inverse agonist/antagonist AM630 (0 µM to 20 µM), while WIN-55,212-2 was kept at a fixed concentration of 2.0 µM, based on the previously determined IC_{50}. The stimulation with both CB ligands was performed simultaneously.

Cell viability assays on HEK293, U87MG and HL-60 cell lines of fourteen synthesized regioisomers were performed (table 1). All compounds were incubated at 10 µM in 200 µL of culture medium supplemented with 10% FBS, in a flat-bottom 96-well plate at a density of 10,000 cells per well, at 37°C for 72 hours. Next, 20 µL of a 5 mg/mL MTT solution was added, and the cells were incubated at 37°C for 4 hours. Then, they were solubilized with 10% sodium dodecyl sulfate (SDS) in 0.1 mM HCl and incubated overnight at 37°C. Formazan absorbance of each well was measured using the EPOCH microplate reader (Biotek, USA) at a wavelength of 570 nm.

---

[1]Mosorov, V., The Lambert-Beer law in time domain form and its application. Applied Radiation and Isotopes, 2017. 128: p. 1-5

Flow cytometry (FC) experiments

Flow cytometry analysis was carried out to determine the mechanism of cell death associated with synthesized regioisomers. 3.0 × 10^5 HL-60 cells were seeded in 24 well tissue culture plates and treated with 0.1% of dimethyl sulfoxide (DMSO) as vehicle control, WIN-55,212-2 as apoptosis positive control, the 4 selected synthesized regioisomers or untreated for 72 h. Then the cells were collected, one sample was treated with cold ethanol for 2 min. to permeabilize the membrane (positive control for necrosis) and centrifuged 300 x g for 5 min. Next, cells were labeled using the Annexin V-FITC apoptosis staining (Thermo Fisher Scientific, MA, USA) and propidium iodide (Ambeed Inc., IL, USA) according to the manufacturer's instructions. This was done by resuspending the pellets of cells in 100 µl of binding buffer with of 10 µl of Annexin V-FITC and 0.1 µ/mL of propidium iodide in the dark for 15 min at room temperature. Then 400 µl of binding buffer were added to dilute the stainig. Flow cytometry analysis was performed using a BD FACSCanto II flow cytometer within one hour.

Molecular docking experiments

Docking simulations were performed for regioisomers **3a**, **3b’**, **3c**, **4b**, WIN-55,212-2 and AM630. Energetic minimization of each molecule were carried out using the LigPrep tool in the program Maestro Schrodinger suite v.11.8 (Schrödinger, LLC)(1). Cannabinoid receptor type 2 structure obtained from cryo-electron microscopy [CB_2 receptor PDBID: 6PT0(2)], were obtained from the Protein Data Bank RCSB-PDB(3). Receptor optimization were performed using the Protein Preparation Wizard available in Maestro software. Water molecules (if applicable) were removed from the protein active site (orthosteric site). Appropriate ionization states for acid and basic amino acid residues, as well as polar hydrogen atoms were considered at physiological pH = 7.4. The enclosing box was configured as a cube with 26 Å length, and the OPLS3e force field was employed for protein energy minimization. The centroid of the selected residue was determined based on the putative orthosteric active site of the CB_2 receptor and its known catalytic amino acids, where the ortostheric ligand WIN-55,212-2 is positioned. The Glide Induced Fit Docking protocol has been used for the final couplings(4). Compounds were punctuated by the Glide scoring function in the extra-precision mode (Glide XP; Schrödinger, LLC)(5,6) and were filtered on the basis of the best scores and best RMS values (less than 1 unit as a cutting criterion), in order to obtain the potential intermolecular interactions between compounds and the receptor, as well as the binding mode and docking descriptors.

[1] Release, S., 2: Maestro, version 11.8. Schrödinger, LLC, New York. 2018.
[2] Xing, C., et al., Cryo-EM structure of the human cannabinoid receptor CB2-Gi signaling complex. Cell, 2020. 180(4): p. 645-654. e13.
[3] Westbrook, J. RCSB protein data bank: Structural biology views for basic and applied research. in ACTA CRYSTALLOGRAPHICA A FOUNDATION AND ADVANCES. 2017. INT UNION CRYSTALLOGRAPHY 2 ABBEY SQ, CHESTER, CH1 2HU, ENGLAND.
[4] Sherman, W., et al., Novel procedure for modeling ligand/receptor induced fit effects. Journal of medicinal chemistry, 2006. 49(2): p. 534-553.
[5] Friesner, R.A., et al., *Extra precision glide: Docking and scoring incorporating a model of hydrophobic enclosure for protein− ligand complexes.* Journal of medicinal chemistry, 2006. **49**(21): p. 6177-6196.
[6] DeLano, W.L., *The PyMOL molecular graphics system.* http://www.pymol.org/,2002.

Molecular dynamics (MD) experiments

Molecular dynamics (MD) simulations of compounds **3a** and **3c** were performed using the Desmond module of the Schrödinger Maestro Suite. The starting point for each simulation was the best docking pose of each compound. Simulations were carried out for 500 ns in an NPT ensemble within a POPE membrane and TIP3P solvent environment, employing the OPLS3e force field. The analyses included interaction residence times, root-mean-square deviation (RMSD), total ligand–protein contacts, and solvent-accessible surface area (SASA).
